# Metabolic engineering of *Yarrowia lipolytica* for thermoresistance and enhanced erythritol productivity

**DOI:** 10.1186/s13068-020-01815-8

**Published:** 2020-10-20

**Authors:** Nan Wang, Ping Chi, Yawen Zou, Yirong Xu, Shuo Xu, M. Bilal, Patrick Fickers, Hairong Cheng

**Affiliations:** 1 State Key Laboratory of Microbial Metabolism, and School of Life Sciences and Biotechnology, Shanghai Jiao Tong University, Shanghai, China; 2 School of Life Science and Food Engineering, Huaiyin Institute of Technology, Huaian, 223003 China; 3 Microbial Process and Interaction, TERRA Teaching and Research Centre, University of Liege – Gembloux Agro-Bio Tech, Gembloux, Belgium

**Keywords:** Erythritol, Erythritol dehydrogenase, Mannitol dehydrogenase, Thermoresistance, *Yarrowia lipolytica*

## Abstract

**Background:**

Functional sugar alcohols have been widely used in the food, medicine, and pharmaceutical industries for their unique properties. Among these, erythritol is a zero calories sweetener produced by the yeast *Yarrowia lipolytica*. However, in wild-type strains, erythritol is produced with low productivity and yield and only under high osmotic pressure together with other undesired polyols, such as mannitol or d-arabitol. The yeast is also able to catabolize erythritol in non-stressing conditions.

**Results:**

Herein, *Y. lipolytica* has been metabolically engineered to increase erythritol production titer, yield, and productivity from glucose. This consisted of the disruption of anabolic pathways for mannitol and d-arabitol together with the erythritol catabolic pathway. Genes *ZWF1* and *GND* encoding, respectively, glucose-6-phosphate dehydrogenase and 6-phosphogluconate dehydrogenase were also constitutively expressed in regenerating the NADPH_2_ consumed during erythritol synthesis. Finally, the gene *RSP5* gene from *Saccharomyces cerevisiae* encoding ubiquitin ligase was overexpressed to improve cell thermoresistance. The resulting strain HCY118 is impaired in mannitol or d-arabitol production and erythritol consumption. It can grow well up to 35 °C and retain an efficient erythritol production capacity at 33 °C. The yield, production, and productivity reached 0.63 g/g, 190 g/L, and 1.97 g/L·h in 2-L flasks, and increased to 0.65 g/g, 196 g/L, and 2.51 g/L·h in 30-m^3^ fermentor, respectively, which has economical practical importance.

**Conclusion:**

The strategy developed herein yielded an engineered *Y. lipolytica* strain with enhanced thermoresistance and NADPH supply, resulting in a higher ability to produce erythritol, but not mannitol or d-arabitol from glucose. This is of interest for process development since it will reduce the cost of bioreactor cooling and erythritol purification.

## Background

In industrialized countries, the overconsumption of sucrose and other specialties including honey, maple syrup, or high fructose corn syrup causes overweight, obesity, diabetes, and metabolic syndrome [1, 2]. As an alternative sweetener, low or non-caloric molecules, such as sugar alcohol, are more and more considered. Among them, erythritol (1,2,3,4-butanetetrol) is a four-carbon polyol with several exciting features. It has very low digestibility and does not raise the blood insulin level. It reduces lipid peroxidation, thereby protecting the damage caused by oxidative stress involved in the pathogenesis of diabetes [3]. It is not metabolized by *Streptococcus mutans,* the main causative agent of tooth decay. Erythritol has been determined as safe for human consumption even at high doses. The maximal recommended dose ranges between 0.6 and 0.8 g/kg of body weight, while it is only around 0.3 g/kg of body weight for the sweetener xylitol [4]. It can also act as an antioxidant in vivo and displays endothelium-protective effects, which may help to protect against hyperglycemia-induced vascular damage [5]. Due to endothelium-protective effects during hyperglycemia, erythritol can reduce the risk of diabetic complications [6].

Erythritol is predominantly produced from glucose by osmophilic yeast such as *Candida magnoliae*, *Y. lipolytica*, *Torula *sp., *Moniliella megachiliensis*, or *Trichosporonoides oedocephalis* [7–10]. Among these, *Y. lipolytica* is the most widely used for industrial erythritol production. Using metabolic engineering, much progress has been gained to improve the erythritol production titer in *Y. lipolytica*. For instance, Janek et al. [11] found that overexpression of the endogenous erythrose reductase gene (i.e., ER, *YALI0F18590g*) resulted in an enhanced erythritol production with the titer of 44.44 g/L, a yield of 0.44 g/g, and productivity of 0.77 g/L/h, which was 20% higher than the parental strain. Carly et al. [12] constructed the *Y. lipolytica* mutant FCY218 by overexpressing *GUT1* (encoding a glycerol kinase, *YALI0F00484g*) and *TKL1* gene (encoding a transketolase, *YALI0E06479g*) and by disrupting the *EYK1* gene (encoding erythrulose kinase, *YALI0F01606g*). The engineered strain exhibited a 75% higher erythritol production titer as compared to that of wild type (80.6 g/L). In *Y. lipolytica* strain CGMCC7326, overexpression of two erythrose reductase genes (*ER10, YALI0D07634g,* and *ER25, YALI0C13508g*) and engineering of the NADPH cofactor metabolism by overexpression of 6-phosphogluconate dehydrogenase genes (*GND1, YALI0B15598g*) and glucose-6-phosphate dehydrogenase genes (*ZWF1, YALI0E22649g*) led to a significant increase in erythritol titer and yield as compared to the wild-type strain (i.e., 190 g/L and 0.63 g/g, respectively) [13]. However, the erythritol production process still suffers from several drawbacks, such as the production of unwanted byproducts such as mannitol and d-arabitol, which renders the purification process more challenging. In these processes, the bioreactor cooling cost could not be neglected at a large scale. Therefore, the improvement of the thermotolerance of the producing strain must also be considered.

To optimize strain CGMCC7326 for erythritol production, it has been impaired for the ability to synthesize mannitol and d-arabitol and to catabolize erythritol. For that purpose, we first identify five genes, namely *AraDH1* (g1595.t1, *YALI0F02211g*), *AraDH2* (g3858.t1, *YALI0E05643g*), *MDH1* (g5130.t1, *YALI0B16192g*), *MDH2* (g2069.t1, *YALI0D18964g*), *XDH1* (g4121.t1, *YALI0E12463g*) and characterized them with d-mannitol or d-arabitol dehydrogenase activities. Then, the d-mannitol dehydrogenase gene that is mainly responsible for byproducts synthesis was deleted, together with gene *EYD1* encoding erythritol dehydrogenase (g1570.t1, *YALI0F01650g*) to disrupt the erythritol utilization pathway. Thirdly, the *rsp5* gene from *S. cerevisiae* encoding ubiquitin ligase was overexpressed, allowing the cell to grow at 35 °C. As the synthesis of erythritol is not a redox-balance reaction, *ZWF1* and *GND1* genes were overexpressed in *Y. lipolytica*, to recycle cofactor NADP. We also disrupt gene *Ku70* involved in non-homologous end-joining to facilitate gene disruption procedure.

## Results and discussion

### Construction of a chassis strain derived from *Y. lipolytica* CGMCC7326

We previously engineered the wild-type strain CGMCC7326 for erythritol production using hygromycin resistance for transformant selection [13]. However, no further genetic modifications could be operated in the resulting strain HCY108 as it is resistant to hygromycin and lacks auxotrophic markers. Therefore, we intended to construct a chassis strain allowing multiple genome editions. For that purpose, we first disrupted gene *Ku70* (*YALI0C08701g*) involved in non-homologous end-joining (NHEJ) to facilitate subsequent gene disruption. We also rendered the resulting strain auxotrophic for uracil by disrupting the *URA3* gene (U40564.1) encoding orotidine 5′-phosphate decarboxylase.

In *Y. lipolytica*, NHEJ, the dominant form of DNA repair, is mediated by the heterodimer protein complex Ku70/Ku80 [14, 15]. It has been reported that the disruption of gene encoding Ku70 resulted in the loss of NHEJ repair and an increased rate of homologous recombination (HR) [16, 17]. Therefore, *Ku70* disruption significantly increases the rate of correct targeted gene replacement to generate knock-out and knock-in mutants, and hence improve the precision of genetic edition in *Y. lipolytica*. The disruption cassette contained upstream and downstream fragments of the *Ku70* gene and the hygromycin resistance gene flanked by loxP and loxR sequences [18]. The *Ku70* disrupted strain was then cured of the hygromycin resistance gene by transient expression of the *Cre* gene encoding loxR/loxP recombinase [18]. This yielded strain HCY109 (*ery929△ku70*) (Table 1).

**Table 1:** Strains, gene cassettes, and primers used in this study

Strains/plasmids/primers	Genotype/sequences (5′ → 3′)	References/restriction sites
*E. coli* strain		
BL21(DE3)	B F^–^ *ompT* *gal* *dcm* *lon* *hsdS*_*B*_(*r*_*B*_^–^*m*_*B*_^–^) λ(DE3 [*lacI* *lacUV5*-*T7p07* *ind1* *sam7* *nin5*]) [*malB*^+^]_K-12_(λ^S^) pLysS	New England BioLabs
TOP10	F– *mcr*AΔ(*mrr*-*hsd*RMS-*mcr*BC) Φ80*lac*ZΔM15 Δ*lac*X74 *rec*A1 *ara*D139 Δ(*ara leu*) 7697 *gal*U *gal*K *rps*L *end*A1 *nup*G	Thermo Fisher Scientific
BL21/AraDH1	BL21(DE3) pET28a-AraDH1	This work
BL21/AraDH2	BL21(DE3) pET28a-AraDH2	This work
BL21/MDH1	BL21(DE3) pET28a-MDH1	This work
BL21/MDH2	BL21(DE3) pET28a-MDH2	This work
BL21/XDH1	BL21(DE3) pET28a-XDH1	This work
*Y. lipolytica* strains		
HCY107 (CGMCC7326, or ery929)	*Suc-*, *Lac-*, *Mal-*	Cheng et al., 2018
HCY109	CGMCC7326 derivative, ery919*△ku70*	This work
HCY109-2	HCY109 derivative, *△ku70△ura3*	This work
HCY110	HCY109 derivative, *△ku70△AraDH1*	This work
HCY111	HCY109 derivative, *△ku70△AraDH2*	This work
HCY112	HCY109 derivative, *△ku70△mdh1*	This work
HCY113	HCY109 derivative, *△ku70△ura3△mdh2*	This work
HCY114	HCY109 derivative, *△ku70△xdh1*	This work
HCY115	HCY113 derivative, *△ku70△ura3△mdh2△eyd1*	This work
HCY117	HCY116 derivative, *△ku70△mdh2△eyd1*::*Sc.rsp5*	This work
HCY118	HCY117derivative, *△ku70△mdh2△eyd1*::*Sc.rsp5*::*zwf1*::*gnd1*	This work
Plasmids		
pUC19	*LacZ*, Amp^R^	
pET28a	*LacI*, T7 promoter, Kan^R^	Novagen
pET28a-ylAraDH1	Containing *ylAraDH1* gene *g1595.t1* (*YALI0F02211g*)	This work
pET28a-ylAraDH2	Containing *ylAraDH2* gene *g3858.t1* (*YALI0E05643g*)	This work
pET28a-ylMDH1	Containing *ylMDH1* gene *g5130.t1* (*YALI0B16192g*)	This work
pET28a-ylMDH2	Containing *ylMDH2* gene *g2069.t1* (*YALI0D18964g*)	This work
pET28a-ylXDH1	Containing *ylXDH1* gene *g4121.t1* (*YALI0E12463g*)	This work
*The above plasmids (except pUC19 and pET28a) were used to transform BL21(DE3) and in the production of ylAraDH1, ylAraDH2, ylMDH1, ylMDH2 and ylXDH1 recombinant enzymes*		
pHB4-621XDH	pHB4-Cre derivative, *hp4d-621XDH-TT-hp4d-Cre*	This work
pWSV-Ku70-loxP-hph-loxP	*up Ku70-loxP-pLeu2-hph-TT-loxP-dw Ku70*	This work
pWSV-AraDH1-loxP-hph-loxP	*up AraDH1-loxP-pLeu2-hph-TT-loxP-dw AraDH1*	This work
pWSV-AraDH2-loxP-hph-loxP	*up AraDH2-loxP-pLeu2-hph-TT-loxP-dw AraDH2*	This work
pWSV-MDH1-loxP-hph-loxP	*up MDH1-loxP-pLeu2-hph-TT-loxP-dw MDH1*	This work
pWSV-MDH2-loxP-hph-loxP	*up MDH2-loxP-pLeu2-hph-TT-loxP-dw MDH2*	This work
pWSV-XDH1-loxP-hph-loxP	*up XDH1-loxP-pLeu2-hph-TT-loxP-dwXDH1*	This work
pWSV-EYD1-loxP-hph-loxP	*up EYD1-loxP-pLeu2-hph-TT-loxP-dw EYD1*	This work
pINA1313-RSP5	*up zeta-ura3-hp4d-RSP5-TT–dw zeta*	This work
*The above plasmids are used to delete ylAraDH1*, *ylAraDH2*, *ylMDH1*, *ylMDH2*, *ylXDH1, PGI genes and overexpress Sc.rsp5 gene for erythritol synthesis*		

Primers	Sequences (5′ → 3′)	Restriction sites
*Primers used for amplification of ylAraDH1, ylAraDH2, ylMDH1, ylMDH2 and ylXDH1 genes and cloned to pET28a by Gibson assembly*		
P_28a-ylAraDH1-F_	CTGGTGCCGCGCGGCAGCCATATGTCTCTCTTTTCACTCGCCAAGAAAAC	*Nde*I
P_28a-ylAraDH1-R_	GTGGTGGTGGTGGTGGTGCTCGAGTTACCAGATGGTGTAACCTCCATCGAC	*Xho*I
P_28a-ylAraDH2-F_	CTGGTGCCGCGCGGCAGCCATATGTCCAACTCCGCCAAAGCCGCTGTC	*Nde*I
P_28a-ylAraDH2-R_	GTGGTGGTGGTGGTGGTGCTCGAGTTAAATAATAGTGTAACCACCATCAATG	*Xho*I
P_28a-ylMDH1-F_	CTGGTGCCGCGCGGCAGCCATATGCCTGCACCAGCAACCTACGCTAC	*Nde*I
P_28a-ylMDH1-R_	GTGGTGGTGGTGGTGGTGCTCGAGCTAAGGACAACAGTAGCCGCCATCAAC	*Xho*I
P_28a-ylMDH2-F_	CTGGTGCCGCGCGGCAGCCATATGATGTCTGGACCTTCCACCCTCGCCAC	*Nde*I
P_28a-ylMDH2-R_	GTGGTGGTGGTGGTGGTGCTCGAGCTAAGGAGCGCAGTAGCCACCATCGAC	*Xho*I
P_28a-ylXDH1-F_	CTGGTGCCGCGCGGCAGCCATATGTCTTCTAACCCGTCATTTGTTCTTC	*Nde*I
P_28a-ylXDH1-R_	GTGGTGGTGGTGGTGGTGCTCGAGCTACTCCTCCTCGGGACCGTCAATGATG	*Xho*I
*The below primers are used for verification of Ku70, ylAraDH1, ylAraDH2, ylMDH1, ylMDH2, ylXDH1, ylEYD1, pgi genes knockout in Y. lipolytica*		
P_Ku70- knockout -F_	ATGGAATGGATTTCACATCTGGAGAACG	
P_Ku70- knockout -R_	TCACTTCCCATAGTACTTTTTGACCAC	
P_AraDH1- knockout -F_	ATGTCTCTCTTTTCACTCGCCAAGAAAAC	
P_AraDH1- knockout -R_	CTCTTGTGGTGCTCTCGAATGGCCTTGA	
P_AraDH2- knockout -F_	ATGTCCAACTCCGCCAAAGCCGCTGTC	
P_AraDH2- knockout -R_	TTAAATAATAGTGTAACCACCATCAATG	
P_MDH1- knockout -F_	ATGCCTGCACCAGCAACCTACGCTAC	
P_MDH1- knockout -R_	CACTGTATTACATCGAGCGAATCCA	
P_MDH2- knockout -F_	ATGTCTGGACCTTCCACCCTCGCCAC	
P_MDH2- knockout -R_	CTAAGGAGCGCAGTAGCCACCATCGAC	
P_XDH1- knockout -F_	ATG TCTTCTAACCCGTCATTTGTTCTTC	
P_XDH1- knockout -R_	CTACTCCTCCTCGGGACCGTCAATGATG	
P_EYD1- knockout -F_	CGAGACTCCCTCTGAGGAGTTCCTG	
P_EYD1- knockout -R_	TTACCAGACGTGGTGGCCACCGCAGAC	
*The below primers are used to construct URA3 gene disruption cassette and verify the corrected URA3 gene mutant*		
P_URA3-upstream-F_	CTGAAACGTTATCTTATATGAACTCC	
P_URA3-upstream-R_	TTTGGTGGTGAAGAGGAGACTGAAATAAATTTAG	
P_URA3-downstream-F_	CTAAATTTATTTCAGTCTCCTCTTCACCACCAAATATGTAATTTAACTGTGTATATAG	
P_URA3-downstream-R_	CAGGCCAGTCCCGCCTCTCCTTTC	
P_URA3-verify-F_	GTGTGCATGATCAAGACCCATATC	
P_URA3-verify-R_	AGGTCGGTTCTGGGCAATGAAGCC	
*Primers used to detect the mRNA levels of ylAraDH1, ylAraDH2, ylMDH1, ylMDH2, ylXDH1 knockout mutants and mRNA levels of Sc.rsp5, ylTKL1 at 30–35 °C*		
P_AraDH1-qPCR-F_	GGTATCGCAGCTGCCAAGCAGCTTC	
P_AraDH1-qPCR-R_	GATTCTGTTTCTGTTTCAGAAAC	
P_AraDH2-qPCR-F_	CGAGCCAACGCCGGATCCAAGGAAG	
P_AraDH2-qPCR -R_	CTCAATGGCATCCAGACCCAGGTC	
P_MDH1-qPCR -F_	GTATCCAAGAACATCATGGAGCG	
P_MDH1-qPCR-R_	CATTTGTAAGCCTTAGATCGGACTCC	
P_MDH2-qPCR -F_	TTCCCCACCAACATCATGGACCG	
P_MDH2-qPCR-R_	CTTGTAGGCCTTGGCCTTGACGCC	
P_XDH1-qPCR-F_	TGCGGCTCGGATGTCCACTACTATC	
P_XDH1-qPCR-R_	GTTGTATCGTCCCTCCTTGTACTC	
P_rsp5-qPCR-F_	TCCGCAGCGAAGAAAACGTTAAATC	
P_rsp5-qPCR -R_	GTCTACCACTCGATGTAGCAGTATC	
P_tkl-qPCR-F_	AAGACTCCCGGCCACCCCGAGGCTG	
P_tkl-qPCR-R_	CTCCGAAGATGCAGTAAGTGTAGTT	
P_*β*-actin-up_	ACTCTGGTGATGGTGTCACCCACG	
P_*β*-actin-down_	TCGGACGATTTCTCGCTCGGCGGAG	

**Fig. 1:**
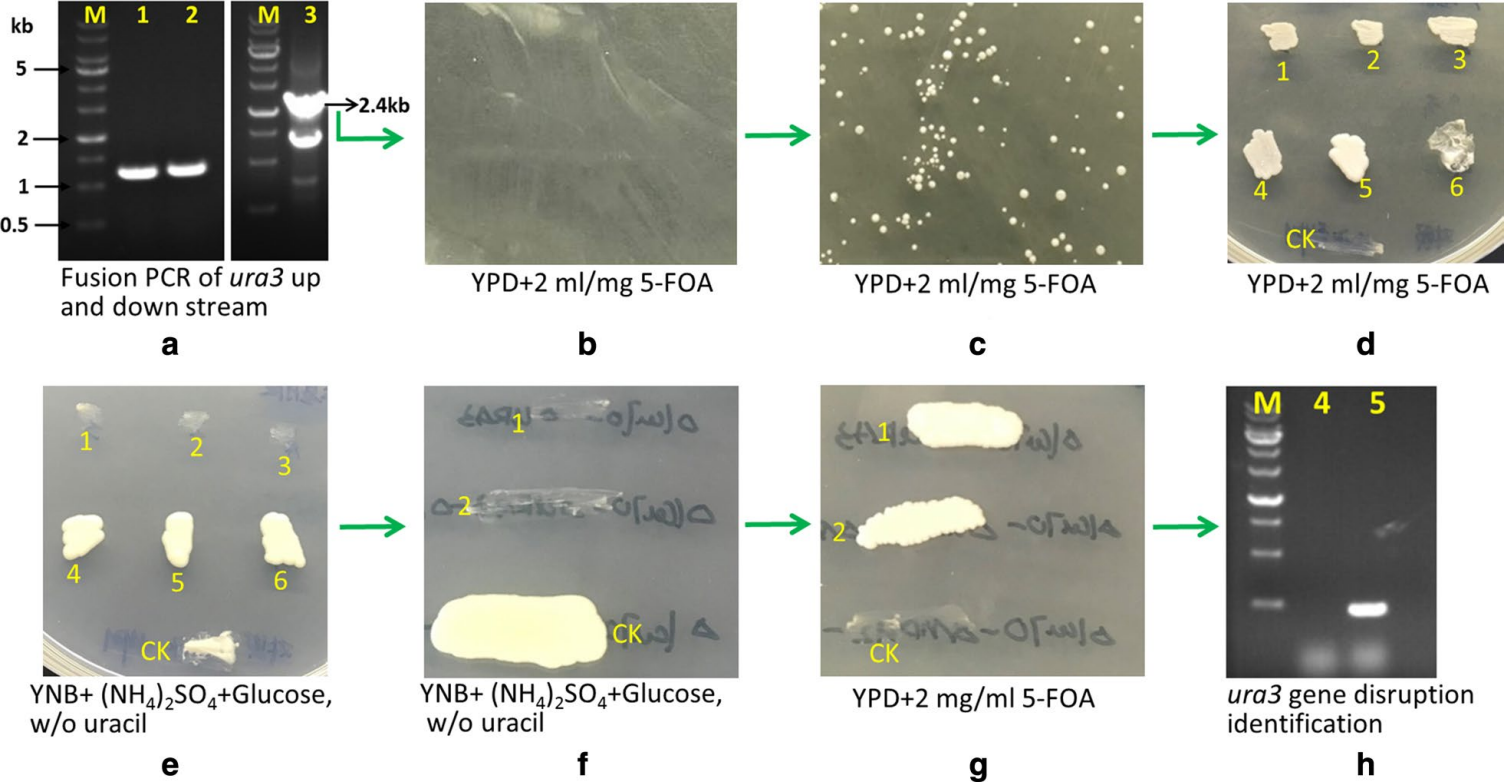
The schematic flow to obtain authentic *URA3* disruption mutant. The 1.2 kb upstream (lane 1, **a**) and downstream (lane 2, **a**) of the *ura3* gene was amplified separately, then the 2.4-kb fragment (lane 3, **a**) was obtained by fusion PCR to merge 1.2 kb up and downstream. Then the 2.4-kb fragment was transformed into HCY109 (ery929△*Ku70*), plated on YPDF medium (10 g/L yeast extract, 5 g/L tryptone, 10 g/L glucose, 2 mg/ml 5-FOA, 15 g/L agar powder, pH 6.5) and cultivated at 30 °C for 5 days (**b**). Colonies were grown (**c**) and transferred to a new YPDF plate cultivated at 30 °C for 5 days (**d**). Then the colonies were transferred to synthetic complex medium without uracil (SC-U, 10 g/L yeast nitrogen base, 5 g/L ammonia sulfate, 10 g/L glucose, without uracil) (**e**). If the *ura3* gene was authentically disrupted, the mutants cannot grow on the SC-U medium (1, 2, 3 in **e**), or they are not authentic *ura3* gene disruption (4, 5, 6 in **e**). Then two putative *ura3*-deficient mutants were streaked on SC-U plate (**f**) and YPDF plate (**g**), they showed opposite growth. A *ura3*-deficient mutant was further to be identified by PCR to amplify the 480-bp fragment in *ura3* gene, and no 480-bp fragment can be amplified (lane 4 in **h**), but the 480-bp fragment can be amplified for the control strain (HCY109, ery929△*Ku70*) (lane 5 in **h**). The No. 1 colony in **g** designated as HCY109-2 was used as the host to conduct metabolic engineering

In *Y. lipolytica* and other yeasts, the *URA3* gene encoding orotidine 5′-monophosphate decarboxylase (OMP) involved in uracil synthesis has been used as a sensitive and versatile auxotrophic marker [18]. Therefore, *URA3* was disrupted in strain HCY109 by gene replacement using a defective allele and a selection medium containing 5-fluoroorotic acid (5-FOA). The resulting chassis strain was designated HCY109-2 (*ery929△ku70△ura3*) (Table 1). Figure 1 shows the schematic flow to obtain the *URA3* disruption mutant.

### Identification of genes encoding enzymes with mannitol/d-arabitol dehydrogenase activities in *Y. lipolytica* CGMCC7326

Although *Y. lipolytica* is one of the best erythritol producers, it also presents some drawbacks such as the synthesis of byproducts including mannitol and d-arabitol. This negatively impacts the yield from glucose and complexifies the purification steps [9, 13, 19–22]. Therefore, we thought of constructing a strain unable to produce these byproducts.

In *Ascomycetes* fungi, the mannitol cycle consists of two pathways [23]. In the first one, fructose-6P from glycolysis is converted into mannitol-1P by a NAD(H)-dependent mannitol dehydrogenase (Mpd). It is then dephosphorylated into mannitol by a mannitol-1P phosphatase. In the second pathway, fructose is oxidized into mannitol by a reversible NADP(H)-dependent mannitol 2-dehydrogenase (Mdh). In *Y. lipolytica*, the mannitol dehydrogenase encoded by gene *YALI0B16192g* has been characterized as an Mdh [24]. Indeed, its disruption impairs mannitol production from fructose, but not from glycerol or glucose. By contrast, its overexpression slightly improves mannitol production from fructose [23]. These findings pointed out that other pathways may exist to produce mannitol from glucose through fructose-6P, such as an Mpd–Mpp like pathway [23]. However, genes involved in that pathway remain unknown in *Y. lipolytica*. d-Arabitol is produced by an arabitol dehydrogenase(s) from ribulose, which is obtained by dephosphorylation of ribulose-5P, an intermediate of the pentose phosphate pathway.

Before disrupting these biosynthetic pathways, the first step was to identify those genes involving mannitol and/or d-arabitol biosynthesis. Genome mining for mannitol dehydrogenase by Dulermo et al. [23] led to the identification of two additional genes, namely Y*ALI0D18964g* and *YALI0B16192g* that were not characterized so far. Further blast analysis, using gene *XP_001387287* from *Scheffersomyces stipitis CBS6054* encoding mannitol dehydrogenase (MDH)-like, classical (c) SDRs (short-chain dehydrogenases/reductases) did not lead to the identification of additional candidate genes. To identify arabitol dehydrogenase encoding gene, the d-arabinitol 2-dehydrogenase gene from *Scheffersomyces stipitis CBS6054* (XP_001385035) and *Wallemia ichthyophaga* (XM_009272203) was used as a query to blast the genome of *Y. lipolytica* CGMCC7326. Gene g1595.t1 (*YALI0F02211g*) was found with 54.73% identity to gene XP_001385035 while gene g3858.t1 (*YALI0E05643g)* showed 54.9% identity to gene XM_009272203. The identity of g1595.t1 and g3858.t1 genes is 44.39%.

Rodriguez et al. [25] revealed the xylose utilization pathway in *Y. lipolytica* and found the gene *YALI0E12463g* (GenBank no. XM_503864) has xylitol dehydrogenase (XDH) with NAD as a cofactor in vitro, when expressed in *E. coli*. When overexpressed in *Y. lipolytica,* Polf along with *XKS* (xylulose kinase gene) resulted in improved growth on xylitol. However, no other polyol substrates were tested with purified *ylXDH* in this article, so it is necessary to determine whether the *ylXDH* has mannitol or d-arabitol dehydrogenase activity and is involved in mannitol or arabitol synthesis or not. The counterpart gene in CGMCC 7326 was g4121.t1, with a 100% identity to *YALI0E12463g*.

Therefore, five genes, which encode putative enzymes with mannitol and/or d-arabitol dehydrogenase were identified, namely *YlAraDH1* (g1595.t1, *YALI0F02211g*), *YlAraDH2* (g3858.t1, *YALI0E05643g*), *YlMDH1* (g5130.t1, *YALI0B16192g*), *YlMDH2* (g2069.t1, *YALI0D18964g*), and *YlXDH* genes (g4121.t1, *YALI0E12463g*) for further biochemical characterization.

### Characterization of purified YlAraDH1, YlAraDH2, YlMDH1, YlMDH2, YlXDH enzymes

**Fig. 2:**
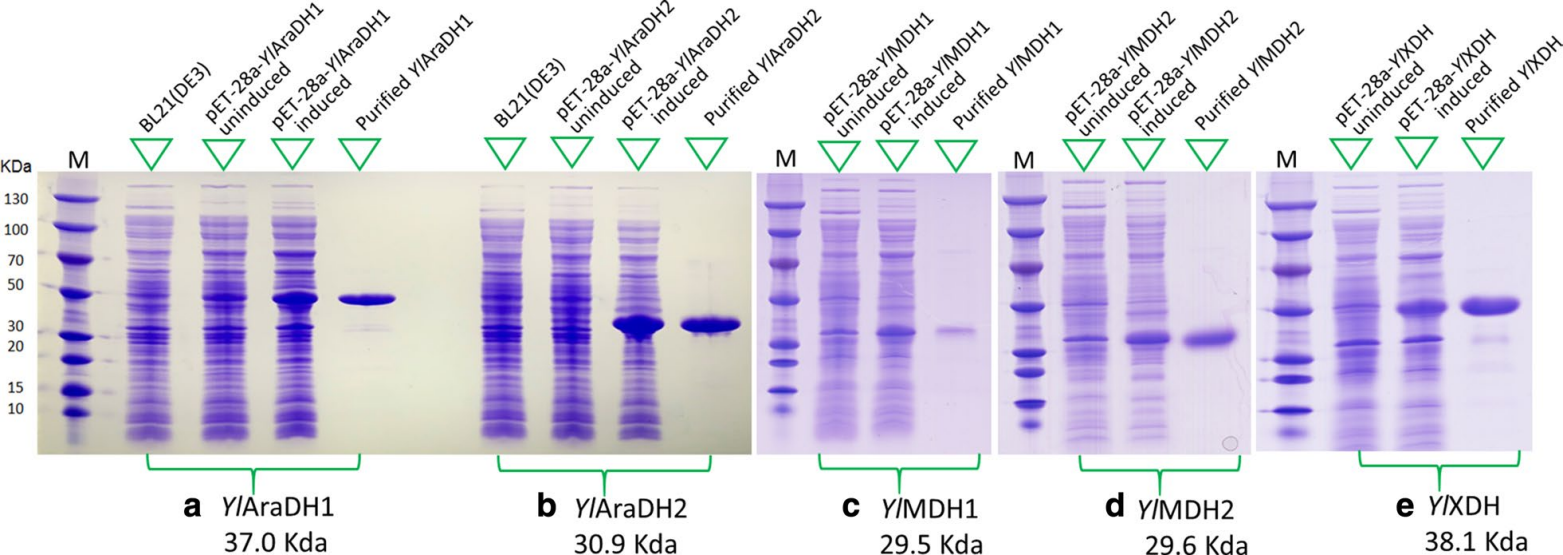
*YlAraDHl*, *YlAraDH2*, *YlMDH1*, *YlMDH2*, *YlXDH* gene expression in *E. coli*

The *YlAraDH1, YlAraDH2, YlMDH1, YlMDH2, YlXDH* encoding genes were expressed in *E. coli,* and the corresponding proteins were purified by Ni–NTA chromatography with the purity of above 95% (Fig. 2). For each purified enzyme, the enzymatic activity, both oxidation, and reduction, was measured for different substrates (Table 2). All enzymes except xylitol dehydrogenase (YlXDH) were able to oxidize mannitol, d-arabitol, xylitol, d-sorbitol. Among these dehydrogenases, YlAraDH1 has the most robust activity towards mannitol, d-arabitol, xylitol, and d-sorbitol, whereas YlMDH1 exhibited the weakest activity toward those four polyols. YlXDH has the strongest xylitol dehydrogenase activity. We also tested other polyols such as ribitol, erythritol, and glycerol as well as primary alcohols (*n*-butanol, isobutanol, ethanol) as substrates. However, no activities were observed for all five enzymes (data not shown). The purified enzymes were also tested for their ability to reduce substrates such as fructose, l-sorbose, or d-xylulose. As shown in Table 2, all tested enzymes except YlXDH were able to reduce these substrates. For substrates such as ribose, mannose, xylose, glucose, and fructose-6P, no activity could be detected (data not shown). According to Table 2, all enzymes have mannitol dehydrogenase activity, and YlAraDH1, YlAraDH2, YlMDH1, YlMDH2 have d-arabitol dehydrogenase activity. Regarding cofactor specificity, YlAraDH1, YlAraDH2, and YlXDH were defined as NAD(H)-dependent, while YlMDH1, YlMDH2 are strictly NADP(H)-dependent. For YlMDH1, these results are in accordance with Dulermo et al. [23] and Napora et al. [24].

**Table 2:** Substrates/cofactors specificity of YlAraDH1, YlAraDH2, YlMDH1, YlMDH2, YlXDH

Substrates (pH^a^)	YlAraDH1^b^ (YALI0F02211p)	YlAraDH2^b^ (YALI0E05643p)	YlMDH1^c^ (YALI0B16192p)	YlMDH2^c^ (YALI0D18964p)	YlXDH^b^ (YALI0E12463p)
	Product (activity^d^)	Product (activity)	Product (activity)	Product (activity)	Product (activity)
Mannitol (pH 8.0)	Fructose (10.3)	Fructose (6.5)	Fructose (2.7)	Fructose (8.4)	Fructose (0.12)
d-Arabitol (pH 8.0)	d-Xylulose (5.3)	d-Xylulose (6.3)	d-Xylulose (0.12)	d-Xylulose (0.83)	-
Xylitol (pH 8.0)	d-Xylulose (3.6)	d-Xylulose (4.6)	d-Xylulose (0.23)	d-Xylulose (1.4)	d-Xylulose (4.35)
d-Sorbitol (pH 8.0)	l-Sorbose (7.5)	l-Sorbose (5.2)	l-Sorbose (0.35)	l-Sorbose (0.86)	Fructose (2.23)
Fructose (pH 6.0)	Mannitol (2.6)	Mannitol (1.7)	Mannitol (0.82)	Mannitol (5.6)	Mannitol (0.04)
l-Sorbose (pH 6.0)	Sorbitol (2.4)	Sorbitol (2.6)	Sorbitol (0.15)	Sorbitol (0.23)	-
d-Xylulose (pH 6.0)	Arabitol (1.4)	Arabitol (2.2)	Arabitol (0.12)	Arabitol (0.54)	Xylitol (2.34)

^a^Reacted at appropriate pH. ^b,c^ With NAD(H) or NADP(H) as cofactor separately. ^d^ U/mg protein. “-”, undetected activity

**Fig. 3:**
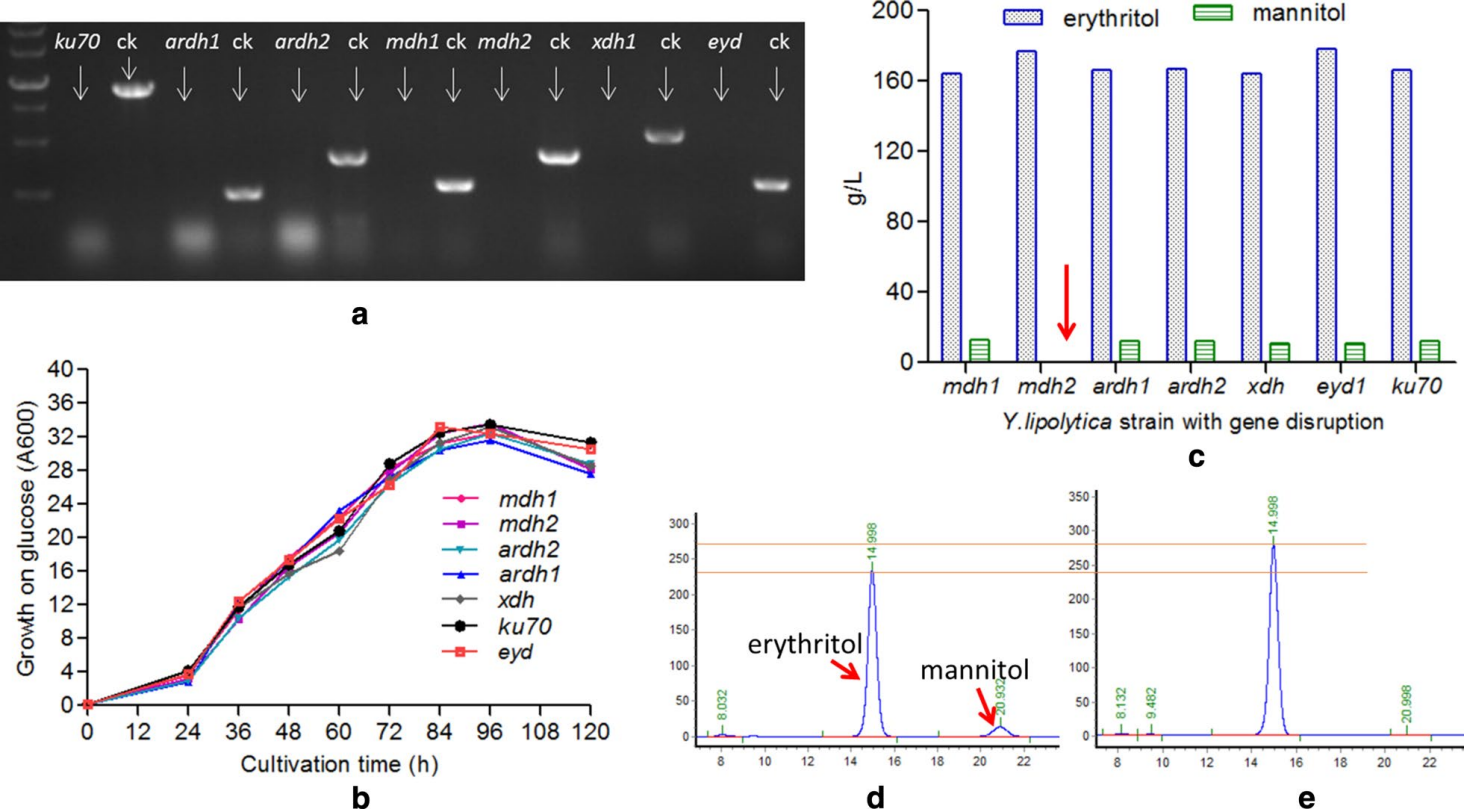
Effects of gene disruption on cell growth, mannitol, and erythritol synthesis. **a** Molecular identification of 7 genes disruption (*Ku70*, *YlAraDH1*, *YlAraDH2*, *YlMDH1*, *YlMDH2*, *YlXDH*, *YlEYD1* genes), no corresponding band can be amplified from the disruptants, but the band can be amplified for wild-type control strain (*Y. lipolytica* CGMCC7326, ery929), indicating *Ku70*, *YlAraDH1*, *YlAraDH2*, *YlMDH1*, *YlMDH2*, *YlXDH*, or *YlEYD1* genes were disrupted individually. **b** Growth curves of different disruptants on liquid medium containing 300 g/L glucose. **c** Effects of various gene disruption on erythritol and mannitol biosynthesis, only *mdh2* gene disruption can result in no mannitol synthesis. **d**, **e** The HPLC chromatography of wild-type strain and *mdh2* gene disruption, showing no mannitol when the *mdh2* gene was disrupted

In order to verify if these enzymes are involved in the synthesis of mannitol and d-arabitol from glucose, the corresponding encoding genes were disrupted in *Y. lipolytica* HCY109. The correctness of the mutant genotype was verified by analytical PCR with primers listed in Table 1. The lack of PCR amplification in mutant strains as compared to the parental strain confirmed the correct gene disruption, namely *YlAraDH1*, *YlAraDH2*, *YlMDH1*, *YlMDH2*, and *YlXDH* (Fig. 3a). The disrupted strains were then grown in a shake flask for 120 h in the YPD_300_ medium_._ As shown in Fig. 3, no significant differences could be observed in both cell growth and polyols synthesis for all the tested disrupted strains as compared to the parental strain (ery929Δ*ku70*Δ*ura3*), except strain HCY113 disrupted for gene *YlMDH2* (*YALI0D18964g*). For this latter, production of both mannitol and d-arabitol was impaired while erythritol production was slightly improved by 7.9%, reached 177 ± 3 g/L from 164 ± 3 g/L (Fig. 3c–e). The above results demonstrate that mannitol is produced from the enzymatic activity of *YlMDH2* as disruption of *YlAraDH1*, *YlAraDH2*, *YlMDH1*, *YlXDH* had no effects on mannitol synthesis. This was further evidenced by the synthesis of mannitol in the *∆YlMDH2* mutant complemented with the *YlMDH2* gene (data not shown). Finally, it is worth mentioning that although YlMDH1 and YlMDH2 shared 74.8% similarity in amino acid, they present different catalytic activities.

**Fig. 4:**
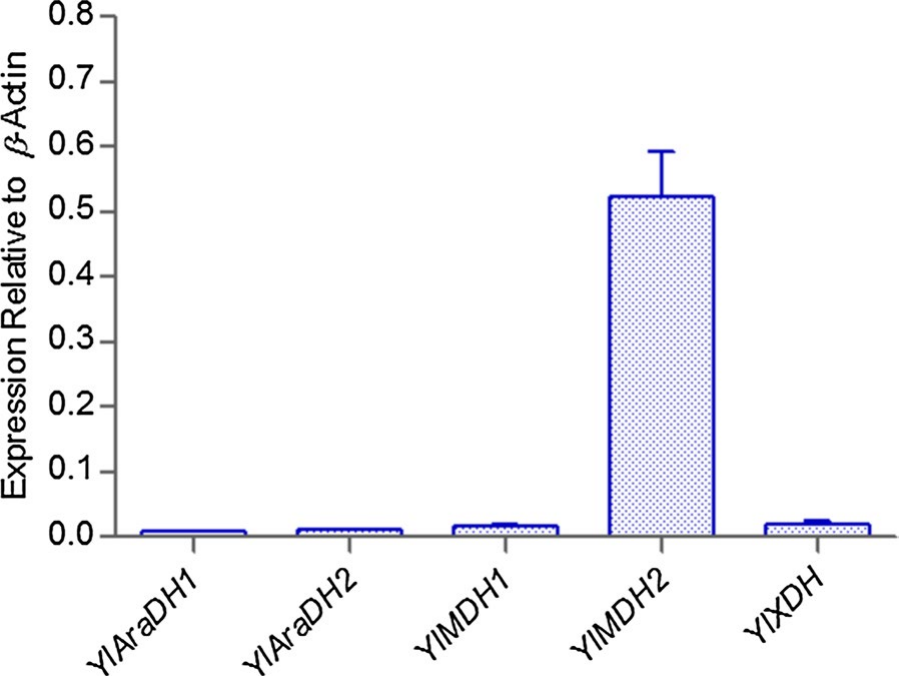
Comparison of *YlAraDH1*, *YlAraDH2*, *YlMDH1*, *YlMDH2*, *YlXDH* expression levels relative to *β*-actin. *YlAraDH1*, *YlAraDH2*, *YlMDH1*, *YlMDH2*, *YlXDH gene* mRNA quantification of wild-type *Y. lipolytica* (CGMCC7326) grown on 300 g/L glucose, mRNA levels are represented as copy number relative to the copy number of *β*-actin

To explain the discrepancy between the enzymatic activity of the purified enzyme in vitro and the phenotype of disruption mutants, we speculated that genes *YlAraDH1*, *YlAraDH2*, *YlMDH1*, *YlXDH* were not or poorly expressed in those experimental conditions (i.e., glucose medium). Therefore, gene expressions were quantified during the growth of wild-type strain CGMCC7326 in the YPD_300_ medium. As shown in Fig. 4, the expression level of gene Yl*MDH2* was high as compared to that of genes *YlAraDH1*, *YlAraDH2*, *YlMDH1*, *YlXDH*.

Dulermo et al. [23] speculated that similarly to other *Ascomycetes,* two pathways may exist in *Y. lipolytica* for mannitol synthesis. Different genes with putative mannitol and/or arabitol dehydrogenase activity have been identified, and the corresponding enzymes were purified. Most of them were found able to reduce fructose into mannitol and xylulose into arabitol in vitro. However, mutant disrupted for gene *YALI0D18964* (Yl*MDH2*) was the only one unable to produce mannitol from glucose, suggesting that Yl*MDH2* is the only active mannitol dehydrogenase inside the cell. This was confirmed by quantification of the gene expression level, which was significantly higher for *YALI0D18964* than for the other tested gene. From the substrate specificity, it may be deduced that Yl*MDH2* is instead a mannitol dehydrogenase (Mdh) than a mannitol-1P dehydrogenase (Mpd). Indeed, it could not reduce fructose-6P, suggesting thus that it is not involved in an Mpd–Mpp-like pathway.

### Disruption of *YlEYD1*

**Fig. 5:**
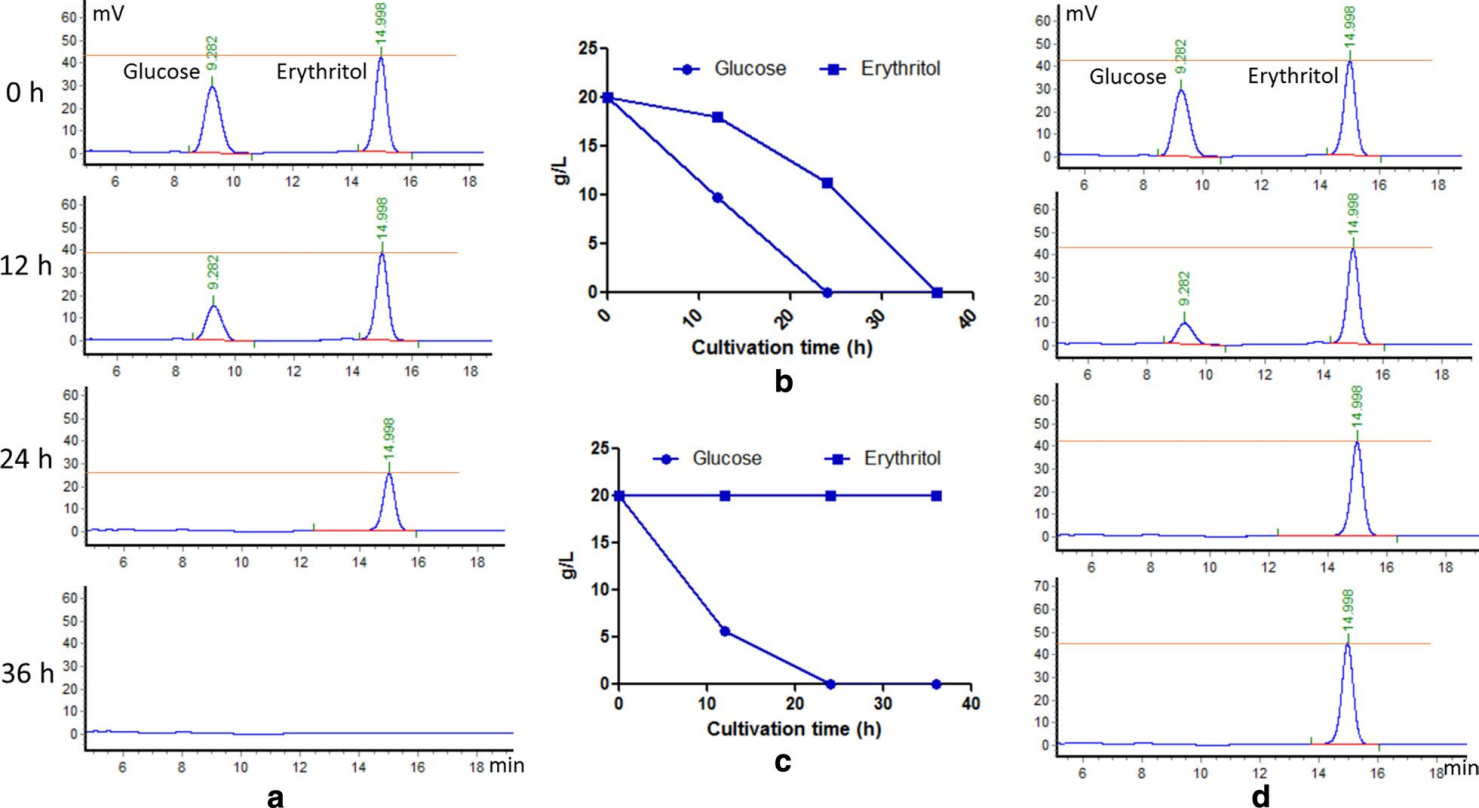
Effect of disruption of *YlEYD1* gene on glucose and erythritol utilization. **a** HPLC profile of glucose and erythritol utilization by wild-type strain harboring the *EYD1* gene. **b** Glucose and erythritol utilization rate by wild-type strain harboring the *EYD1* gene. **c** Glucose and erythritol utilization rate by the *EYD1* gene disruption strain. **d** HPLC profile of glucose and erythritol utilization by the *EYD1* gene disruption strain

Although *Y. lipolytica* can synthesize erythritol in response to osmotic stress, it also has the ability to reconsume it in isotonic conditions. In this catabolic pathway, erythritol is first converted into erythrulose by an erythritol dehydrogenase encoded by gene *EYD1* [26]. Therefore, the gene was disrupted in strains HCY109-2 (*△ku70△ura3*) and HCY113 (*△ku70△ura3△mdh2*). Gene disruption in the chassis strain HCY109-2 yielded an 8% increase in erythritol titer (178 ± 3 g/L, Fig. 3C) as compared to the parental strain (HCY113). Glucose and erythritol consumption rates were determined during the culture of strains HCY113 (*△ku70△ura3△mdh2*) and HCY115 (*△ku70△ura3△mdh2△eyd1*) in a medium containing 20 g/L glucose and 20 g/L erythritol. As shown in Fig. 5, strain HCY115 was unable to catabolize erythritol while it showed a fivefold increased glucose uptake rate (0.83 g/L h) in the first 12 h of growth. At the industrial scale, the erythritol production process is operated at high glucose concentration (up to 300 g/L). In these conditions, no erythritol is reconsumed by the cell due to the high osmotic pressure. However, at the end of the production process, glucose must be depleted in the culture medium for ease of erythritol purification. It is known that at low glucose concentration (around 20 g/L and below), erythritol starts to be reconsumed by the cell together with the remaining glucose. To avoid this erythritol consumption, the erythritol catabolic pathway could be disrupted. Carly et al. [27] disrupted *EYK1* encoding erythrulose kinase, the second enzyme of the pathway. Although this strategy led to mutant strain impaired in erythritol catabolism, the disrupted strain was still able to convert erythritol into erythrulose leading to a mixture of erythritol and erythrulose. To avoid this, we disrupted the first gene of the pathway, *EYD1*.

### Improving thermoresistance by expression of *Sc.rsp5* gene in *Y. lipolytica*

**Fig. 6:**
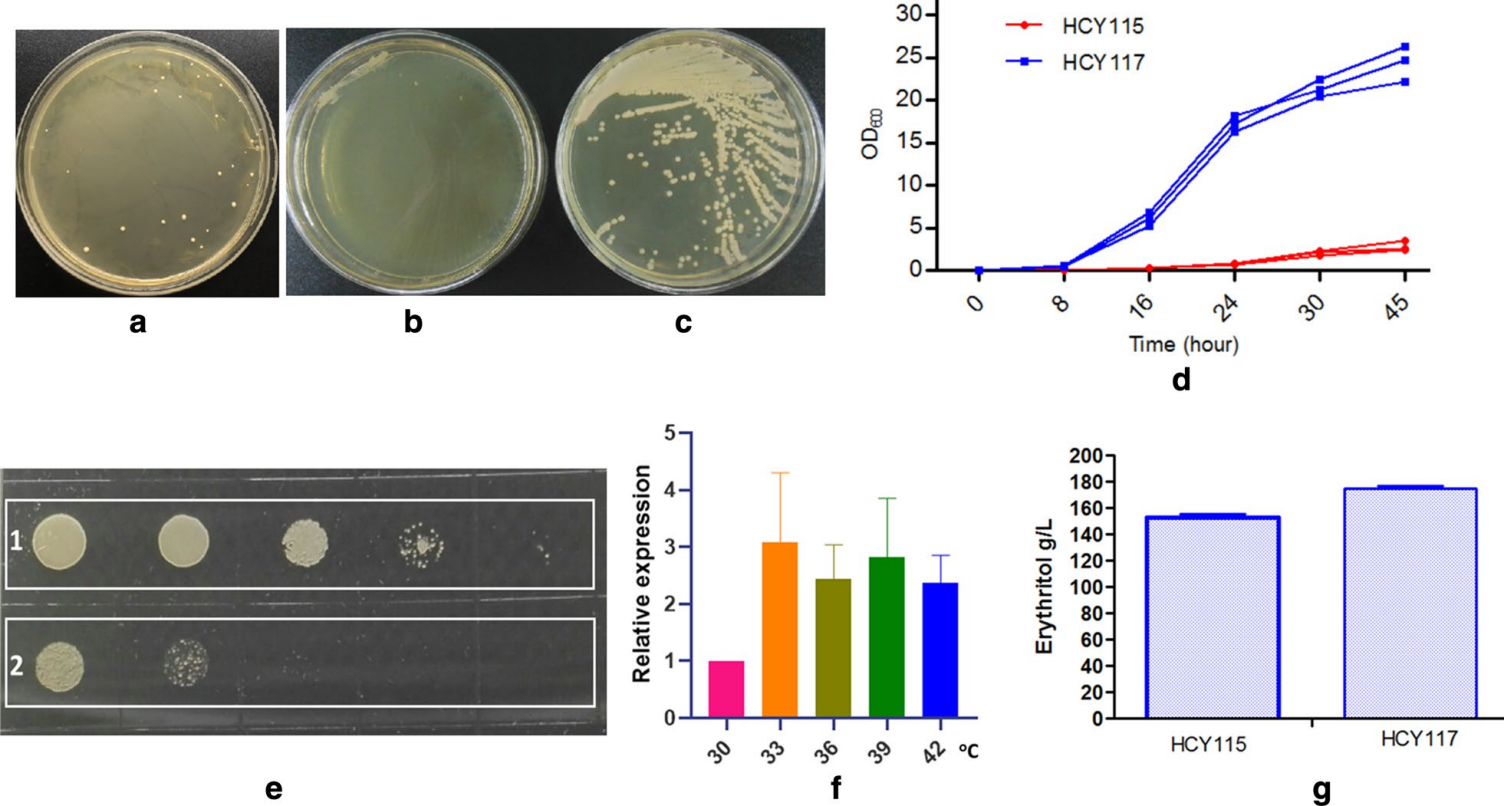
Characterization of HCY117 overexpressing *S. cerevisiae rsp5* gene. **a** Transformants containing *Sc*.*rsp5* gene can grow at 34 °C; **b** parental strain HCY115 cannot grow at 34 °C; **c** HCY117 strain can grow well at 34 °C; **d** growth profiles of HCY115 and HCY117; **e** HCY117 can grow well after heat-shocked at 45 °C for 60 min (1), whereas the parental strain HCY115 grow poorly (2); **f** *Sc.rsp5* gene relative expression at different temperatures; **g** erythritol production of HCY115 and HCY117

The optimal growth temperature of *Y. lipolytica* ranges between 28 and 30 °C [13, 28, 29], and at higher temperature, cell growth ability decreases drastically. Yeast metabolism could be considered as an exothermic process for instance; glucose catabolism has a specific heat production of 1798 kJ/mol [30]. Since *Y. lipolytica* is known to be able to grow at high cell density and to sustain high glucose concentration, the energy, and thus the cost, requested for bioreactor cooling is to take into account, especially at industrial scale. Therefore, increasing the thermotolerance of strain HCY115 (*Δku70Δura3Δmdh2Δeyd1*) for several degrees is of interest to develop an efficient erythritol production process. Several strategies have been used for that purpose in yeast, such as overexpression of heat shock proteins (HSP) or transcription factors [31–34]. In *Saccharomyces cerevisiae*, overexpression of gene *RSP5* encoding ubiquitin ligase allowed the cell to grow up to 41 °C [35]. Therefore, the *S. cerevisiae RSP5* gene was expressed in strain HCY115 (*Δku70Δura3Δmdh2Δeyd*) to yield strain (HCY117, *Δku70Δura3Δmdh2Δeyd-php4d-rsp5*, *php4d* hereinafter is *hp4d* promoter). The HCY117 strain can grow well at 34 °C (Fig. 6) and efficiently produce erythritol at 33 °C.

Transform *Y. lipolytica* HCY115 with *Sc.rsp5* gene expression cassette (pINA1313-Sc.rsp5, cut with *Not*I) were spread on YPD plate and allowed to cultivate at 34 °C. Colonies can grow at 34 °C 7 days later (Fig. 6a). Then one colony grew at 34 °C from the plate (Fig. 6a), and the control strain (HCY115, without *Sc.rsp5* gene) were picked up, cultivated on new plates at 34 °C. The control strain grew poorly at 34 °C (Fig. 6b), but the HCY117 strain can boost well (Fig. 6c). When grown in a liquid medium, the growth is the same as that on solid plate medium (data not shown). The control strain HCY115 grows poorly at 34 °C, but HCY117 can grow well with a cell density of 24.4 ± 2.2 at 45 h (Fig. 6d). To investigate the thermoresistance, the control strain HCY115 and *Sc.rsp5* harboring strain HCY117 were heat-shocked at 45 °C for 60 min, then diluted by tenfold and cultivated on the YPD plate at 30 °C for 48 h. The *Sc.rsp5* harboring strain HCY117 can grow well after heat-shocked at 45 °C for 60 min (Fig. 6e, 1), whereas the control strain HCY115 grows poorly (Fig. 6e, 2).

In order to evaluate whether the *Sc.rsp5* gene in HCY117 was induced under higher temperature, the cells of *Sc.rsp5* harboring strain HCY117 was heat treated at 30, 33, 36, 39, and 42 °C for 4 h, and total RNA were extracted. *Sc.rsp5* mRNA quantification indicates that the *Sc.rsp5* gene can be induced at a higher temperature, the mRNA level is two to threefold than that of at 30 °C (Fig. 6f).

HCY 117 (*Δku70Δmdh2Δeyd-php4d-rsp5*) can produce more erythritol at 300 g/L glucose at 33 °C than that of HCY115 (*Δku70Δura3Δmdh2Δeyd*), with 175 ± 5 g/L erythritol in 96 h via 154 ± 5 g/L in 120 h at 33 °C (Fig. 6g), indicating that harboring *rsp5* gene of *S. cerevisiae* renders thermoresistance for *Y. lipolytica*.

**Fig. 7:**
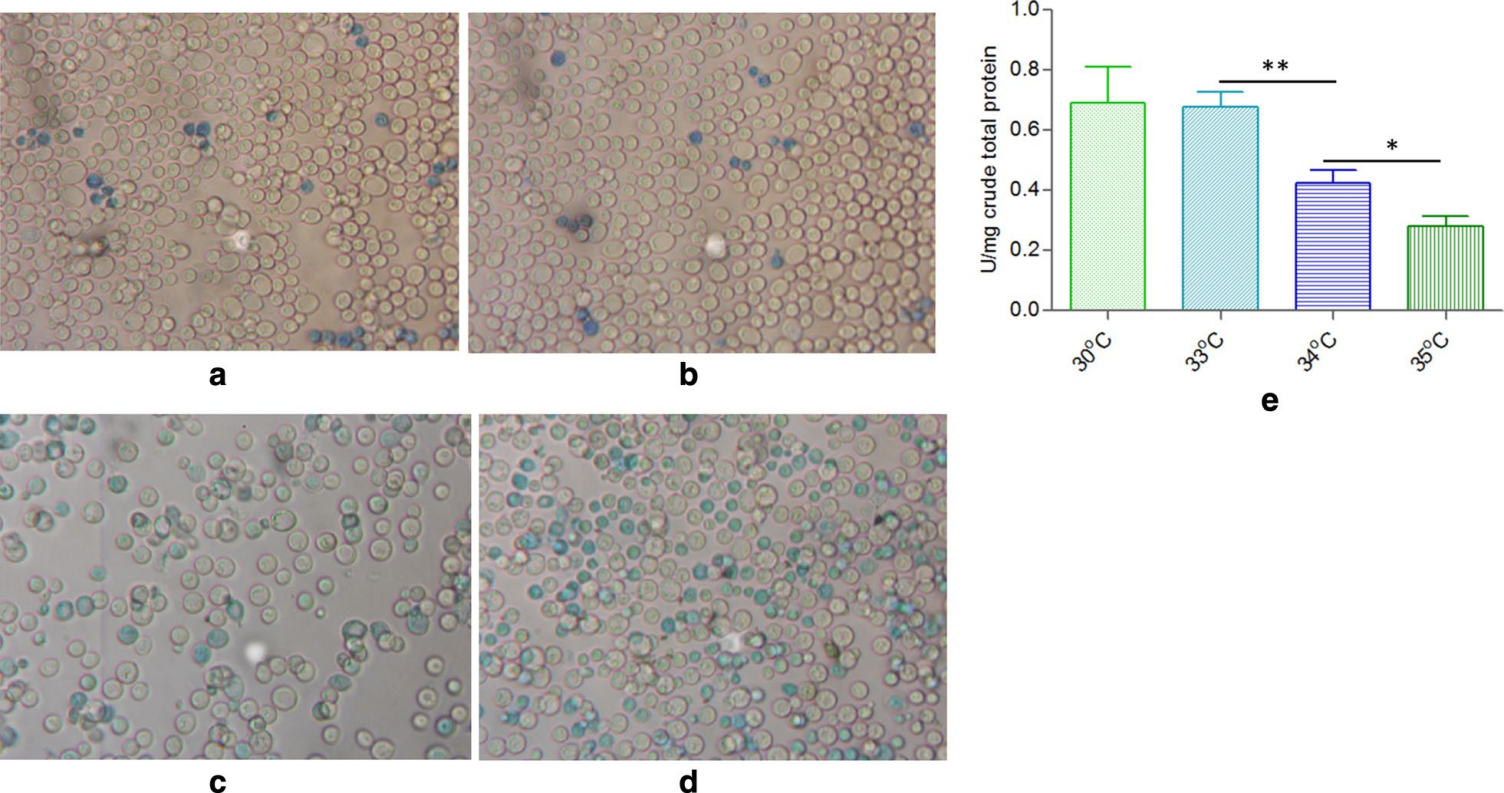
Cell viability and TKL1 activity of HCY118 at different temperatures. **a** Cell viability stain at 30 °C; **b**: at 33 °C; **c**: at 34 °C; **d**: at 35 °C; E: TKL1 activity at 30, 33, 34 and 35 °C. Blue stained cells indicated that these cells have low or no viability

As to the acidic and alkaline conditions are the stress factors for *Y. lipolytica*, heat shock was also the stress factor for *Y. lipolytica*, which may cause an increase in antioxidant protection [36, 37]. This process cannot be prevented by adaptive induction of the enzymes responsible for antioxidant protection under high temperature, which results in cell damage and decreases survival rate and causes the decreased enzyme activities involved in PPP, which is responsible for erythritol synthesis. A quick method for estimating the percentage of viable cells in a yeast population is to use alkaline methylene blue staining, which is very simple and highly sensitive [38]. When cells were stained with alkaline methylene blue solution with a pH of 10.6 at 25 °C for 5 min, the percentage of unstained cells was consistent with viability measurements made using the slide culture method. Using this method, the cell viability or survival rate of HCY117 at 34 °C and 35 °C is lower than that of at 33 °C and 30 °C. At 30–33 °C, the stained blue cells (low or no viability cells) account for 5–7% of total cells (Fig. 7a, b), while at 34 °C and 35 °C, the ratio increased to 35% and 50% (Fig. 7c, d). The transketolase activity at 30 °C, 33 °C, 34 °C and 35 °C was 0.69 ± 0.12, 0.67 ± 0.06, 0.43 ± 0.05, and 0.28 ± 0.04 U/mg total crude protein, respectively (Fig. 7e), indicating that the higher temperature can impair transketolase activity thus lead to less erythritol synthesis from glucose (Table 3). Under stress conditions such as at higher temperature, both reactive oxygen species (ROS) generation and the contribution of antioxidant components to the cell viability is higher. It was shown that mannitol plays the role of a potent scavenger of hydroxyl radicals (HO·) and ROS both in vitro and in vivo [36, 37, 39]. The involvement of mannitol in antioxidant protection of fungal cells was also revealed in *S. cerevisiae* [40]. As for the HCY117 strain, from which no mannitol was synthesized again since the *YlMDH2* gene was disrupted, the cell viability may be lower under stress conditions such as at higher temperatures, for example, at above 33 °C, though the *Sc.rsp5* gene can be well expressed.

**Table 3:** Summary of fermentation profiles of engineered strain HCY118 and wild-type control strain at 30, 33, 34, 35 °C

Strains	Temp	Erythritol (titer: g/L)	Mannitol (titer: g/L)	Q_ery_ (g/L h)	Y_ery_ (g/g glucose)	T (h)	R_glu_ (g/L h)	OD_600_	DCW (g/L)	CP_ery_ (g/g dcw h)
CGMCC7326 (wild-type control)	30 °C	166 ± 5.5	7.5 ± 0.5	1.73	0.55	96	3.13	32.4 ± 1.5	8.1	0.22
	33 °C	145 ± 5.5	12.4 ± 0.5	1.21	0.48	120	2.5	28.8 ± 1.5	7.2	0.17
HCY118	30 °C	189 ± 6	0	1.97	0.63	96	3.13	30.5 ± 1.5	7.6	0.26
	33 °C	190 ± 6	0	1.97	0.63	96	3.13	32.6 ± 1.5	8.2	0.24
	34 °C	162 ± 5.5	0	1.2	0.54	135	2.22	24.4 ± 1.5	6.1	0.16
	35 °C	132 ± 4	0	0.78	0.44	170	1.76	20.6 ± 1.5	5.3	0.11

Q_ery_: productivity; Y_ery_: erythritol yield; R_glu_: glucose consumption rate; DCW: dry cell weight; CP_ery_: cell production rate

### Improve the NADPH supply in the strain HCY118 and erythritol production

As described by Cheng et al. [13], the reduction of erythrose by erythrose reductases needs NADPH as a cofactor. Therefore, regenerating this cofactor from NADP is a key factor in increasing erythritol productivity in strain HCY117. The first reaction of the phosphate pentose pathway (PPP) pathway, catalyzed by the glucose-6P dehydrogenase (G6PD or ZWF), generates NADPH from glucose-6P and NADP^+^. In yeast, it has been suggested that G6PD has a major role in NADPH production [41]. The 6-phosphogluconate dehydrogenase (GND), which catalyzes the third reaction of the PPP pathway, also generates NADPH using 6-phosphogluconate as a substrate. In *Y. lipolytica*, these two enzymes are encoded by gene *ZWF1* (*YALI0E22649g*) and *GND1* (*YALI0B15598g*), respectively [42]. Therefore, overexpressing these two genes in the strain HCY117 (*Δku70Δura3Δmdh2Δeyd-php4d-rsp5*) will regenerate the NADPH consumed by d-erythrose reductase. Moreover, the flux of carbon (glucose-6P from glycolysis) will be redirected toward the PPP pathway and produce more d-erythrose, the intermediate of erythritol. Therefore, the *ZWF1* and *GND1* were constitutively expressed in strain HCY117 by transforming expression cassette *26S rDNA-zwf1-hph-gnd1-26S rDNA* (sequence 9 in Additional file 1) into HCY117 (*Δku70Δmdh2Δeyd::rsp5*), yielding the strain HCY118 (*Δku70Δmdh2Δeyd::rsp5::zwf1::gnd1*).

**Fig. 8:**
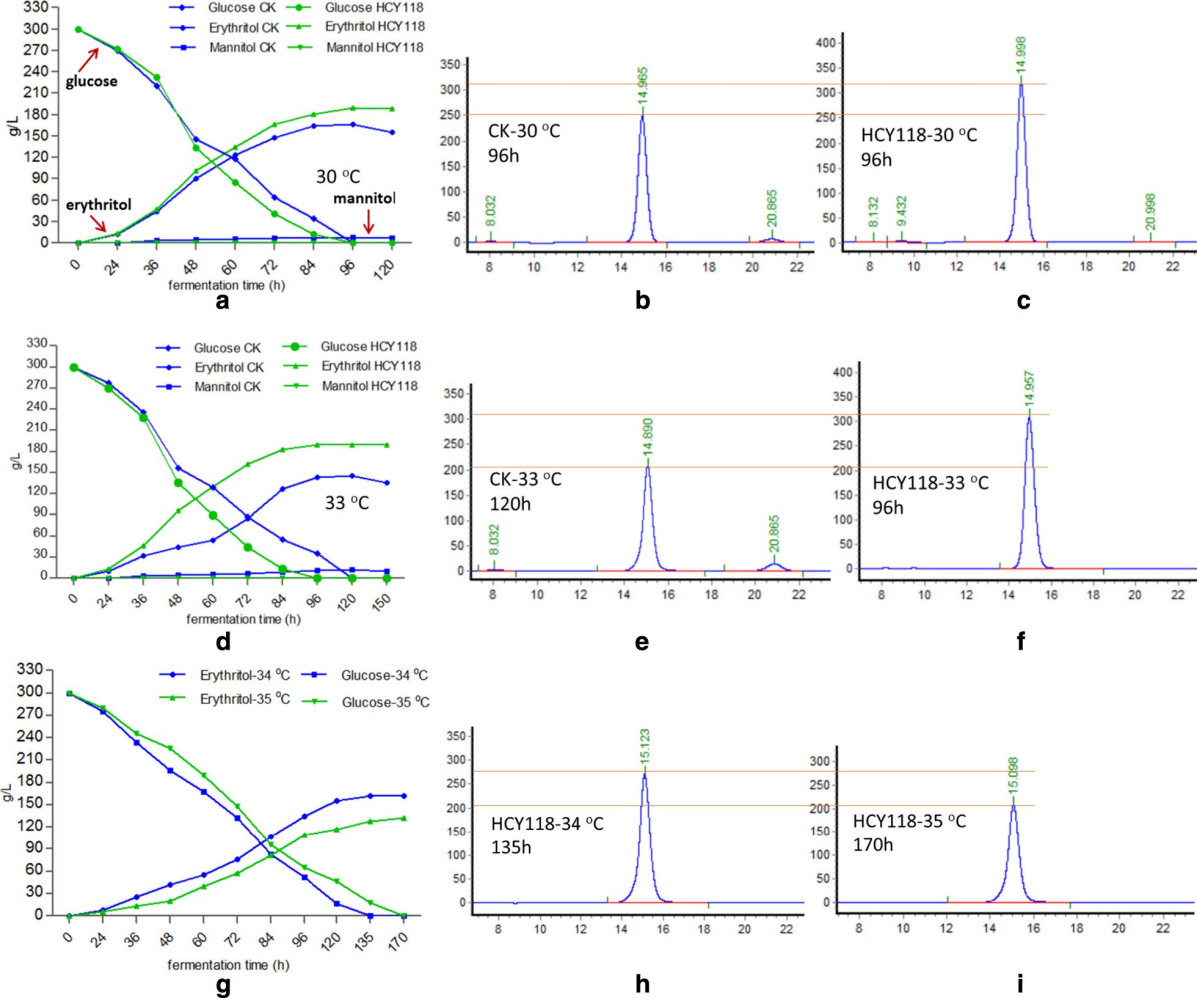
Erythritol production by *Y. lipolytica* strain HCY118 at different temperatures. The strain HCY118 and wild-type strain were cultured in rich medium containing 300 g/L glucose at 30 °C (**a**), 33 °C (**d**), 34 °C and 35 °C (**g**). **b**, **c** HPLC profiles of wild type and HCY118 at 30 °C. **e**, **f** HPLC profiles of wild type and HCY118 at 33 °C. **h**, **i** HPLC profiles of HCY118 at 34 and 35 °C

Then the erythritol production capacity of the engineered strain HCY118 was evaluated during shake-flask culture at 30, 33, 34, and 35 °C, and compared to that of the wild-type strain CGMCC7326. At 30 °C, strain CGMCC7326 produced erythritol with a titer of 166 ± 5.6 g/L erythritol, yield, and productivity of 0.55 g erythritol/g glucose and 1.73 g/L h, respectively (Fig. 8a, b, Table 3). By contrast, for HCY118 erythritol titer, yield and productivity were equal to 189 ± 6 g/L, 0.63 g erythritol/g glucose, and 1.97 g/L h, respectively (Fig. 8a, c, Table 3). This represents an increase of 13.8%, 14.5%, and 13.9% for production titer, yield, and productivity, respectively, as compared to the control strain CGMCC7326. No significant differences were observed for cell growth and glucose consumption rate between HCY118 and parental strain at 30 °C (Table 3). However, for that latter, erythritol content decreased from 166 to 155 g/L after 120 h. This was not the case for strain HCY118, which is unable to reconsume erythritol even after 120 h of culture (Fig. 8a). Furthermore, HCY118 did not produce mannitol (Fig. 8c), while the control produces 7.5 g/L byproduct mannitol (Fig. 8a, b, and Table 3).

At 33 °C, for HCY118, the production, yield, productivity, cell biomass, glucose consumption rate, cell production rate, and fermentation time were almost the same to those at 30 °C (Fig. 8d, f, Table 3). But for the control strain CGMCC7326, the production, yield, productivity, cell biomass, glucose consumption rate, the cell production rate was decreased compared to those at 30 °C, and mannitol amount and fermentation time increased (Fig. 8d, e, Table 3). The production titer, yield, productivity, and cell production rate for HCY118 was improved by 31%, 31.3%, 62.8%, 41.15%, respectively, as compared to that of strain CGMCC7326 at 33 °C.

Since the production remarkably decreased for the control strain at 33 °C, we did not conduct the fermentation process above 33 °C for the strain CGMCC7326. Then the erythritol fermentation was conducted only for HCY118 at 34 and 35 °C to evaluate whether it retains the high production or not above 33 °C. Results indicated that the production, yield, productivity, cell biomass, cell production rate, and glucose consumption were decreased at 34 and 35 °C when the fermentation time increased to 135 and 170 h at 34 and 35 °C for HCY118 (*Δku70Δmdh2Δeyd::rsp5::zwf1::gnd1*) (Fig. 8g–i, Table 3). At 34 °C/35 °C, the production, yield, productivity, and cell production rate were reduced by 14.7%/30.5%, 14.3%/30.2%, 39.1%/60.4%, 29.2%/50% than those of at 33 °C, respectively. Though strain HCY118 can grow well at 34 °C and 35 °C with a high cell density of 24.4 and 20.6 after 135 and 170 h fermentation, respectively, its production, yield, productivity, and cell production rate decreased sharply with increasing temperature (Table 3), even though from 33 increased to 34 °C, only 1 °C degree was improved, indicating that erythritol synthesis is very sensitive to high temperature. Therefore, 33 °C was the temperature limit at which the HCY118 strain can retain the maximum erythritol production.

To verify the results achieving from 2-L baffled flasks, the pilot and large-scale industrial experiments were conducted separately in 150-L and 30-cubic meter fermentors containing 300 ± 10 g/L glucose at 33 °C, compared to that of wild-type strain CGMCC7326. The results are shown in Table 4. After 85 ± 2 h fermentation, HCY118 production reached 188 ± 5 g/L erythritol, with yield and productivity of 0.617 g erythritol/g glucose and 2.18 g/L h in a 150-L fermentor, slightly higher than that of in 2-L baffled flasks (1.97 g/L h). Interestingly, in 30-cubic meter fermentors the fermentation time was reduced to 78 ± 2 h, the erythritol titer increased to 196 ± 2 g/L, with a yield and productivity of 0.653 g erythritol/g glucose and 2.51 g/L h, much higher than that in 2-L baffled flasks. The higher titer, yield, and productivity obtained in 150-L and 30-cubic meter fermenters might be due to better aeration since erythritol synthesis in *Y. lipolytica* is a process of strictly aerobic respiration, and oxygen supply has a huge impact on erythritol synthesis by *Y. lipolytica*. Oxygen concentration has an effect not only on the mitochondrial electron transport chain, but also on the cytochrome content in cells, which determines the level of energy supply for various metabolic processes, including erythritol and citric acid synthesis in *Y. lipolytica* [42–44].

**Table 4:** The fermentation profiles of engineered strain HCY118 at 33 °C in 150-L and 30-cubic meter fermentors

Strains	Fermentor volume	Erythritol (g/L)	Mannitol (g/L)	Q_ery_ (g/L h)	Y_ery_ (g/g glucose)	T (hour)	R_glu_ (g/L h)	OD_600_	DCW (g/L)	CP_ery_ (g/g dcw h)
CGMCC7326 (wild type)	150 L	156 ± 5	6.8 ± 0.5	1.3	0.52	120	2.5	27.6 ± 1.5	6.9	0.19
	30 m^3^	165 ± 5	7.2 ± 0.5	1.57	0.55	105	2.86	31.8 ± 1.5	8.0	0.20
HCY118	150 L	188 ± 5	0	2.21	0.627	85 ± 2	3.53	34.5 ± 1.5	8.6	0.26
	30 m^3^	196 ± 2	0	2.51	0.653	78 ± 2	3.85	36.8 ± 1.5	9.2	0.27

Q_ery_: productivity; Y_ery_: erythritol yield; R_glu_: glucose consumption rate; DCW: dry cell weight; CP_ery_: cell production rate

## Conclusions

In summary, we first identified five putative genes encoding enzymes that have mannitol/ d-arabitol activity in *Y. lipolytica* and found only when disruption of *YlMDH2* can result in no byproduct synthesis. By disruption of the *YlMDH2* and *YlEYD* genes, the strain lost its ability to synthesize mannitol, resulting in no byproduct and easy to purify erythritol from the fermentation medium. It cannot utilize erythritol again, leading to an increase in erythritol titer. Then *Sc.rsp5*, *zwf1,* and *gnd1* genes were expressed in acquiring the thermoresistance to grow up to 35 °C and synthesize maximum erythritol production at 33 °C, 3 °C higher than that of control strain, which would reduce the cooling cost. Combining such factors, the production, yield, productivity, and cell production rate for HCY118 was improved by 31%, 31.3%, 62.8%, and 41.15%, respectively, compared to control strain CGMCC7326 at 33 °C. The yield, production, and productivity reached 0.63 g/g, 190 g/L, and 1.97 g/L·h in 2-L flasks and increased to 0.65, 196 g/L, and 2.51 g/L h in 30-m^3^ fermentor, respectively, which has economically practical importance.

## Future perspectives

**Fig. 9:**
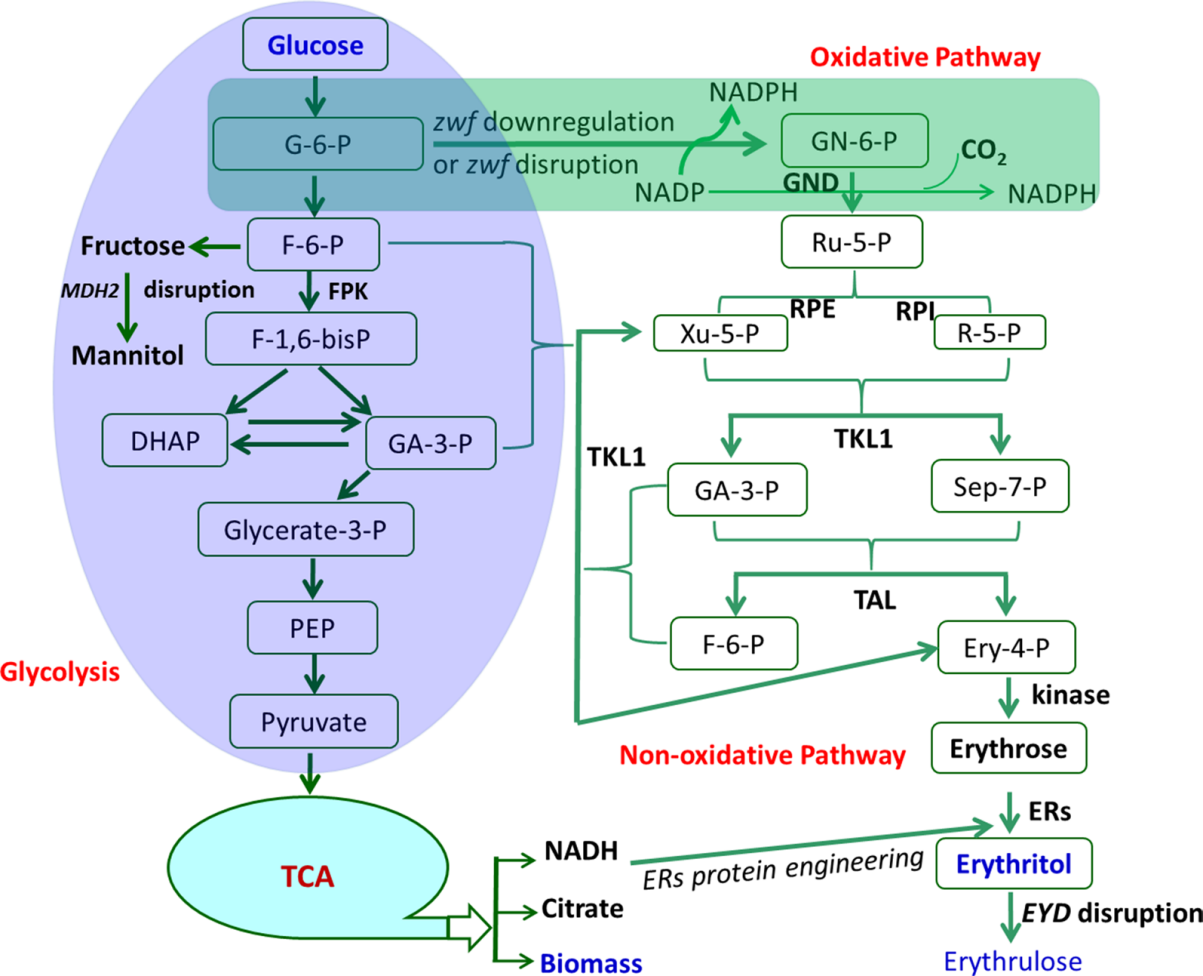
The putative chart for erythritol biosynthesis from the non-oxidative pathway. In this pathway, the oxidative phosphate pentose pathway (in the green box) was blocked by disrupted *zwf* gene or downregulated by inhibition of *zwf* gene expression by replacing its native promoter with a weak or inducible promoter. *TKL1* gene was upregulated by overexpression and fructose 6-P and glyceraldehyde 3-P were converted to xylulose 5-P and erythrose 4-P, which is the intermediate of erythritol. Xylulose 5-P can be reversibly converted into ribulose 5-P by RPE, then ribulose 5-P can be converted to ribose 5-P by RPI. Ribose 5-P and xylulose 5-P were converted to fructose 6-P and erythrose 4-P by TKL1 and TAL. The non-oxidative pathway (in pink box) process involves no carbon loss and the carbon atom economy can be improved. NADPH-dependent ERs can be modified by protein engineering to accept NADH as a cofactor, which is generated from the TCA cycle. Since no carbon atom or reduced carbon atom was lost via the oxidative pathway, erythritol production or yield could be further improved

Erythritol was mainly synthesized from glucose via PPP, which includes oxidative and non-oxidative pathways. The oxidative pathway regenerates two molecular NADPH by the successive oxidation process, and one carbon atom of 6-phosphate gluconate was released as CO_2._ The 6-phosphate gluconate was converted to 5-phosphate ribulose, which was fluxed into a non-oxidative pathway, and finally converted into d-erythrose, which then reduced to erythritol by NADPH-dependent erythrose reductases (ERs). Based on the oxidative and non-oxidative pathways, the highest erythritol production reported so far is hard to exceed 200 g/L erythritol from 300 g/L glucose; at least 100 glucose was exhausted via aerobic respiration to produce biomass, citric acid, and CO_2_. However, cell biomass (DCW) produced was not more than 9 g/L (this study), and citric acid was less than 12 g/L [13], so about 80 g/L glucose was exhausted to produce CO_2_, part of which was produced by oxidative PPP. Therefore, downregulating the oxidative PPP would provide another strategy to further increase erythritol production from glucose by which more glucose was channeled into non-oxidative pathway by transketolase, which converts glycolysis intermediates 6-phosphate fructose and 3-phosphate glyceraldehyde to 5-phosphate xylulose and 4-phosphate erythrose, which then convert to d-erythrose by dephosphorylation, then reduced to erythritol by strict NADPH-dependent ERs. Strict NADPH-dependent ERs can be modified to be both NADH and NADPH-preferred by protein engineering. Through the non-oxidative pathway and ERs cofactor engineering, the carbon atom economy of glucose can be improved, and erythritol production and yield are further enhanced. As an alternative, the phosphoketolase gene, which catalyzes 6-P fructose to 4-P erythrose and acetyl-P (AcP) could be introduced into *Y. lipolytica* to increase the amount of intermediate 4-P erythrose [45], to increase erythritol production. The chart of erythritol synthesis from the non-oxidative pathway is illustrated in Fig. 9. This research is being undertaken in our group.

## Materials and methods

### Chemicals and biological reagents

d-mannitol, d-sorbitol, d-arabitol, xylitol, ribitol, erythritol, glycerol, d-fructose-6-P, and d-fructose were obtained from Sigma-Aldrich (St. Louis, United States). Coomassie brilliant blue R-250, cofactors NADP, NAD, NADH, NADPH, and antibiotics were purchased from Sangon Biotech (Shanghai, China). New England Biolabs or Takara supplied all restriction enzymes. High-fidelity KOD-Fx, 2 × ready-to-use qTaq mixture for PCR were purchased from Toyobo (Japan). RNA extraction kit, reverse transcription kit, and qPCR kit were obtained from Vazyme Biotech Co., Ltd (Nanjing, China). All other chemicals were of analytical grade.

### Strains, media, and culture conditions

The *Escherichia coli* and *Y. lipolytica* strains used in this study are listed in Table 1. The *E. coli* strains were grown at 37 °C in Luria–Bertani (LB) medium supplemented with ampicillin (100 mg/L) or kanamycin sulfate (50 mg/L). The *Y. lipolytica* strains were grown at 30 °C in YPD (10 g/L yeast extract, 5 g/L tryptone, and 10 g/L dextrose) or YNB medium (10 g/L yeast nitrogen base without amino acids, 5 g/L ammonia sulfate) supplemented with glucose (20 g/L, YNBS), or xylitol (20 g/L, YNBX). Hygromycin was added to the YPD when necessary to screen transformants. For erythritol fermentation, the enriched medium (YPD_300_ medium) was employed (per L): 300 g anhydrous glucose, 10 g yeast extract, 5 g peptone, 3.5 g ammonium citrate, 3.0 g (NH_4_)_2_HPO_4_, 0.1 g/L MgSO_4_·7H_2_O, 0.01 g MnSO_4_·H_2_O, 0.01 g ZnSO_4_·7H_2_O and 0.005 g CuSO_4_·5H_2_O, 0.1 mL antifoaming agent (organic silicon defoamer), initial pH 6.5. For solid media, agar (15 g/L) was added. The carbon and nitrogen sources were sterilized separately at 121 °C for 20 min to avoid the Maillard reaction.

Shake-flask cultures for erythritol production were performed in triplicate using 2-L baffled flasks, each containing 300 mL enriched medium, at 30 °C, 33 °C, 34 °C, 35 °C and 220 rpm. Cultures were performed until glucose was depleted. All the baffled Erlenmeyer flasks that contain enriched medium were weighed at the start of fermentation and then before taking sample aliquots for analysis. At the time of sampling, the resulting reduced weight was replenished by the addition of the same weight of sterilized water.

The pilot and large-scale experiment were conducted in 150-L and 30-m^3^ fermentors, which contained 100 L and 22.5 m^3^ of the above-enriched medium and fermented at 33 °C at an agitation of 300 rpm for 150-L fermentor and 100 rpm for 30-m^3^ fermentor, with initial pH 5.80–6.10 without adjustment during fermentation. The aeration rate maintains between 0.3 to 0.5 vvm during fermentation. At the end of fermentation, erythritol was analyzed by HPLC. Biomass (cell dry weight, CDW) was measured as the optical density of the cultures at 600 nm absorbance, based on the measurement of 1 mL medium with 1 OD_600_ unit being equal to 0.25 mg/mL cell dry weight.

### Screening of genes encoding enzymes with d-mannitol/d-arabitol dehydrogenase activities in *Y. lipolytica* CGMCC7326

Blocking the synthesis pathway of byproducts has critical value in the erythritol industry. To stop the pathway, it is necessary first to identify genes responsible for d-mannitol and d-arabitol synthesis. We used mannitol dehydrogenase (MDH)-like, classical (c) SDRs of non-conventional yeast *Scheffersomyces stipitis CBS6054* (*XP_001387287*) as a query to search the *Y. lipolytica* CGMCC7326 genome for acquiring the putative mannitol dehydrogenase enzyme gene. We also used the d-arabinitol 2-dehydrogenase gene of *Scheffersomyces stipitis CBS6054* (*XP_001385035*) and *Wallemia ichthyophaga*, the most halophilic fungus [46] as queries to blast the genome of *Y. lipolytica* CGMCC7326 for putative d-arabitol dehydrogenase genes.

### Cloning of the putative mannitol or arabitol dehydrogenase genes in *E. coli* expression vector

The putative mannitol or arabitol dehydrogenase genes were PCR amplified from the genomic DNA of strain CGMCC7326 using primers listed in Table 1. Forward (F) and reverse (R) primers (name according to gene nomenclature) were designed to introduce *Nde*I and *Xho*I sites in the resulting amplicons, respectively. The corresponding PCR fragments were cloned into pET28a expression vector at the *Nde*I and *Xho*I sites by Gibson assembly, yielding five plasmids pET28a-ylAraDH1, pET28a-ylAraDH2, pET28a-ylMDH1, pET28a-ylMDH2, pET28a-ylXDH1. Strain *E. coli* BL21(DE3) was then transformed with the different constructs containing *ylAraDH1*, *ylAraDH2*, *ylMDH1*, *ylMDH2,* or *ylXDH1* genes.

### Enzyme purification

A single colony of recombinant *E. coli* BL21 (DE3) was grown in 5 mL of LB medium with 100-μg mL^−1^ kanamycin in 30-mL universal tubes at 37 °C for 3 h with shaking at 220 rpm. Afterward, 5 mL of cells were transferred to 245 mL of LB medium with 100 μg mL^−1^ kanamycin in a 2-L Erlenmeyer flask and incubated at 37 °C (at 220 rpm) until the OD_600_ reached 1.0. After the addition of isopropyl-*β*- d-thiogalactopyranoside (IPTG) at a final concentration of 1 mM, the cultures were further incubated at 16 °C for 24 h. Appropriately, 250 mL of cells were harvested by centrifugation (at 12,000*g* and 4 °C for 10 min), washed with 100 mL binding buffer (20 mM Tris–HCl pH 8.0, 200 mM NaCl, 1 mM PMSF, and 2 mM β-mercaptoethanol and resuspended in 100 mL of the same buffer. The cells were disrupted through a continuous high-pressure cell disrupter, and cell debris was eliminated by centrifugation at 10,000*g* for 10 min at 4 °C. The cell extract was filtered through a 0.45-μm membrane filter (Millipore).

Recombinant enzymes from cell lysates were purified using Ni-nitrilotriacetate agarose (Ni–NTA) according to the manufacturer’s recommendation. Proteins were eluted with a buffer containing 25 mM NaH_2_PO_4_–150 mM NaCl–200 mM imidazole (pH 8.0). The Ni–NTA-purified enzymes were further applied to gel filtration on Sephadex G-25 column equilibrated with 100 mM Tris–Cl (pH 7.5) containing 1 mM DTT. Protein concentrations were determined spectrophotometrically by the Bradford method with bovine serum albumin as a standard [47]. The purity of prepared enzymes was evaluated by denaturing SDS-PAGE using an acrylamide concentration of 12% (wt/vol) for the separating gel and 4.5% (wt/vol) for the stacking gel. Gels were subsequently stained for proteins with Coomassie blue R250 stain and decolored with methanol and acetate.

### Determination of enzyme activities

The determination of enzymes with mannitol dehydrogenase (MDH) activity was carried out as reported earlier [48, 49] with some modifications. MDH activity was measured by monitoring the oxidation of NADH or NADPH (or reduction of NAD or NADP) at 340 nm. The reduction of fructose or fructose 6-P was assayed in 100 mM sodium acetate buffer (pH 6.0) containing 2 mM NADPH or NADH, purified enzyme, and 100 mM d-fructose or fructose 6-P in a total volume of 1.5 mL. The reactions were initiated by adding d-fructose or fructose 6-P in the mixtures. The oxidation of mannitol was assayed in 100 mM Tris–Cl (pH 8.0) containing 2 mM NADP or NAD, purified enzyme, and 100 mM d-mannitol in a total volume of 1.5 mL. One unit of activity was defined as the amount of enzyme which catalyzes the oxidation of 1 μmol of NADPH or NADH per min. For cofactor specificity, enzyme activity for mannitol (100 mM) was determined as described above using cofactor (NAD or NADP) at a concentration of 2 mM. To study the substrate specificity, other polyols such as d-sorbitol, d-arabitol, xylitol, ribitol, erythritol, glycerol were also tested. The reaction mixture contained (1.5 mL final volume) 50 mM substrate, 2 mM NADP or NAD, 50 μL of purified enzymes, and 10 mM Tris–Cl buffer (pH 8.0). Specific activity was expressed as U/mg of protein.

Transketolase (TKL) activities were assayed by measuring the decrease in NADH concentration at 340 nm [50]. Briefly, TKL activity was measured at 30 °C in a reaction mixture with the following composition: 84 mM triethanolamine buffer (pH 7.6), 0.9 mM xylulose 5-phosphate, 1.2 mM ribose 5-phosphate, 0.3 mM thiamine pyrophosphate, 0.3 mM NADH, 0.34 U of GDH, and 1 U of TIM. In this assay, xylulose 5-phosphate and ribose 5-phosphate are used as substrates for TKL, and the enzymatic formation of glyceraldehyde 3-phosphate in a coupled reaction with GDH and TIM is measured. One unit of enzyme activity was defined as the amount of enzyme that oxidizes 1 μmol NADH per min. These enzyme activities were determined using a UV/VIS-2450 spectrophotometer (Agilent).

### Construction of the *Ku70* deletion mutant and marker excision

The *Ku70* gene of erythritol-producing strain *Y. lipolytica* CGMCC7326 was 100% similar to that of *Y. lipolytica* CLIB122 with gene tag *YALI0C08701g*. However, the identities of 1.2 kb upstream sequence of *Ku70* in CGMCC7326 and CLIB122 was 90%, the identity of 1.2 kb downstream sequences of *Ku70* in CGMCC7326 and CLIB122 was only 41%, these differences indicating the genome of the two strains are somewhat different. The *Ku70* deletion cassette sequence is shown in Additional file 1 (sequence 1), containing the marker gene hygromycin resistance (*hph*) gene. The full-length *Ku70* deletion cassette (4.3 kb) was synthesized in vitro and cloned into the pUC19 derivative vector to yield the *Ku70* gene knockout vector pSWV-*ΔKu70-hph*, in which two loxP sites were located at the flanking sequence of *hph* sequence. Then the *Ku70* deletion cassette was cut with *EcoR*I and *Not*I to release the 4.3 kb fragment, which was transformed into *Y. lipolytica* CGMCC7326 by lithium acetate transformation [13]. The cells were plated on YPD-hph solid medium containing 400 μg/mL hygromycin B. Transformants were transferred to a new YPD-hph medium, cultivated for 4 days, and repeated to obtain pure clones. Then total genomes of transformants were extracted according to Cheng [51]. Primers P_Ku70-knockout-F_ and P_Ku70-knockout-R_ listed in Table 1 were used to verify the *Ku70* gene disruption. *Hph* gene marker was rescued from *Ku70::hph* by transformation with the replicative plasmid pUB4-XDH, in which the *Gluconobacter oxydans* 621H xylitol dehydrogenase gene (*XDH*) was used to replace the *hph* gene of pUB4-Cre [18]. The fragment *EcoR*I-hp4d-621XDH-TT-hp4d-Cre-*Sal*I (sequence 2 in Additional file 1) was synthesized and used to replace the 4050 bp *EcoR*I-*Sal*I fragment of pUB4-Cre yielding the rescuing plasmid pUB4-XDH. After the plasmid pUB4-XDH rescued the *hph* gene, the transformant was cultivated again in YPD liquid medium without hygromycin B and xylitol to lose the episomal plasmid pUB4-XDH, giving rise to the strain ery929*△Ku70* (HCY109 in Table 3), which was used as chassis to delete or express other genes, for example, the *URA3* and *EYD1* genes.

### Construction of the URA3 deletion mutant based on *ery929△Ku70* (HCY109)

To disrupt the *ylURA3* gene in *ery929△Ku70* (HCY109), *ylURA3* disruption cassette was constructed by overlap PCR. The 1.2 kb upstream of the *ylURA3* gene (GenBank accession no. YLU40564, 861 bp) was amplified using primers P_URA3-upstream-F_ and P_URA3-upstream-R_, the 1.2 kb downstream of the *ylURA3* gene was amplified using primers P_URA3-downstream-F_ and P_URA3-downstream-R_. Then the 1.2 kb upstream and 1.2 kb downstream of *ylURA3* fragments were ligated by overlapping PCR with primers P_URA3-upstream-F_ and P_URA3-downstream-R_, yielding the 2.4-kb *ylURA3* gene disruption cassette and was verified by DNA sequencing. The 2.4-kb fragment was used to transform HCY109 and plated on a solid YPD plate containing 2 mg/mL fluoroorotic acid (5-FOA) to negatively select *URA3* gene-deleted strains. The transformants grown on 5-FOA containing YPD were transferred to the SD medium without uracil (SD-U) to select stable *URA3*-deficient mutants. The transformants that were unable to grow on the SD-U plate were verified by PCR with primers P_URA3-verify-F_ and P_URA3-verify-R_ to amplify the fragment of 480 bp in the *URA3* gene. The authentic *URA3* disruption strain was designated as HCY109-2 (*ery929△Ku70△URA3*).

### Deletion of genes encoding mannitol dehydrogenases and/or d-arabitol dehydrogenases

Genes encoding the mannitol and d-arabitol dehydrogenases were deleted to verify whether mannitol and d-arabitol can still be synthesized in erythritol-producing *Y. lipolytica* CGMCC7326 (hereinafter as ery929). Five genes were identified to have both mannitol and d-arabitol dehydrogenase activities in ery929 genome: (1) d-arabitol dehydrogenase gene 1 (AraDH1, gene locus was g1595.t1 in ery929 and its counterpart is YALI0F02211g in *Y. lipolytica* CLIB122 CLIB122), (2) d-arabitol dehydrogenase gene 2 (AraDH2, gene locus was g3858.t1 in ery929, and its counterpart is YALI0E05643g in CLIB122), (3) d-mannitol dehydrogenase gene 1 (MDH1, gene locus was g5130.t1 in ery929, its counterpart is YALI0B16192g in CLIB122), (4) d-mannitol dehydrogenase gene 2 (MDH2, gene locus was g2069.t1 in ery929, its counterpart is YALI0D18964g in CLIB122), and (5) xylitol dehydrogenase gene (XDH1, gene locus was g4121.t1 in ery929, its counterpart is YALI0E12463g in CLIB122).

The sequences of the five genes deletion cassettes are shown in the Additional file 1. All genes contained the marker gene hygromycin resistance (*hph*) gene, full-length synthesized in vitro, and cloned into the pUC19 derivative vector to yield the corresponding gene knockout vector, in which two *loxP* sites were located at the flanking sequence of *hph* sequence. For instance, *AraDH1* gene knockout cassette (1.5 kb upstream of AraDH1-loxP-hph-loxP-1.5 kb downstream) (total 4.4 kb) was synthesized and cloned into pUC19-derived vector to yield *AraDH1* gene knockout vector pSWV-*ΔAraDH1-hph* (sequence 3 in Additional file 1). Similarly, the other four knockout vectors were constructed in the same strategy. Then the knockout vectors were linearized with *EcoR*I and *Not*I to release the knockout cassettes and were transformed into HCY109 (*Y. lipolytica* ery929*ΔKu70*), respectively. The identification of deletion mutants was verified according to the above method using the primers listed in Table 1. The identified *AraDH1*, *AraDH2*, *MDH1*, *MDH2*, *XDH1* gene-deleted mutants were designated as ery929*ΔKu70Δura3ΔAraDH1* (HCY110), ery929*ΔKu70Δura3ΔAraDH2* (HCY111), ery929*ΔKu70Δura3ΔMDH1* (HCY112), ery929*ΔKu70Δura3ΔMDH2* (HCY113), ery929*ΔKu70Δura3ΔXDH1* (HCY114).

### Functional identification of mutants deficient in *ylAraDH1*, *ylAraDH2*, *ylMDH1*, *ylMDH2* or *ylXDH1* genes

To verify whether *ylAraDH1*, *ylAraDH1*, *ylMDH1*, *ylMDH2,* and *ylXDH1* gene deletion can block byproducts mannitol or d-arabitol synthesis during the erythritol fermentation, the wild-type *Y. lipolytica* CGMCC7326 and mutant strains (HCY110, HCY111, HCY112, HCY113, and HCY114) were inoculated in YPD_300_ medium for erythritol synthesis. Shake-flask cultures for erythritol production were performed in triplicate using 2-L baffled flasks containing 250 mL rich medium (YPD_300_), at 30 °C and 220 rpm. Cultures were performed until glucose was depleted.

### Construction of *EYD1* gene-deletion mutants and marker excision

The *EYD1* gene knockout cassette was also synthesized and contained the marker hygromycin resistance gene (*hph*) gene, in which two loxP sites were located at the flanking sequence of *hph* sequence. The synthesized cassette was cloned into the pUC19 derivative vector to yield pWSV-EYD1-loxP-hph-loxP (sequence 8 in Additional file 1), which was cut with *EcoR*I and *Not*I to release the 4.3 kb fragment to transform the strain HCY113 (*△Ku70△ura3△MDH2*). Identification of *EYD1* gene-deletion mutants was verified according to the above method using the primers P_EYD1- knockout –F_ and P_EYD1- knockout –R_ listed in Table 3. The transformation of plasmid pHB4-621XDH rescued the *hph* gene. The identified *EYD1* gene-deleted mutants were designated as HCY115 (quadruple mutant, *△Ku70△ura3△MDH2△EYD1*). The fermentation verification of the HCY115 strain was performed as the method used above.

### Overexpression of *S. cerevisiae RSP5* gene to improve thermoresistance of HCY115 strain

The ubiquitin ligase gene *RSP5* of *S. cerevisiae* [35] was overexpressed in HCY115 (*△Ku70△ura3△MDH2△EYD1*). The *S. cerevisiae RSP5* gene was cloned into the pINA1313 vector to give plasmid pINA1313-ScRSP5 (containing *ura3* marker), which was cut with *Not*I to linearize this plasmid, then transformed to HCY115 strain, and was plated on YPD and cultivated at 34 °C. PCR verified transformants with the pair of primers P_ScRSP5-F_ and P_ScRSP5-R_. The corrected strain was designated as HCY117 (*△Ku70△MDH2△EYD1:: ScRSP5*, no longer *ura3*-deficient due to revertant by *ura3* gene in pINA1313). The erythritol fermentation and growth of the HCY117 strain were evaluated in the medium YPD_300_ at 30 °C to 35 °C.

### Overexpression of genes *ZWF1* and *GND1* in HCY117

*ZWF1* (*YALI0E22649g*) and *GND1* (*YALI0B15598g*) genes from *Y. lipolytica* were overexpressed in the HCY117 strain using *Not*I treated *ZWF-GND* expression cassette (5′-*26S rDNA-hp4d-zwf1-TT-hph-hp4d-gnd1-TT-26S rDNA*-3′, (Sequence 9 in Additional file 1), transformed into HCY117 and plated onto YPDH medium (YPD + 400 μg/mL hygromycin). Transformants grown on this medium were identified by PCR to verify the extra *ZWF-GND* gene and to verify their expression level by qPCR. The true strain was designated as HCY 118 (*△Ku70△MDH2△EYD1*::*ScRSP5*:: *ZWF1-GND1*). The erythritol fermentation and growth of the HCY118 strain were evaluated in the medium YPD_300_ at 30–35 °C compared with the wild-type strain CGMCC7326 (ery929).

### RNA isolation and transcript-level quantification

Shake-flask cultures were grown in rich medium. Cells were collected at an OD_600_ of 2.0 and stored at − 80 °C in the Trizol solution. Total RNAs were extracted using liquid nitrogen and the TRIzol kit from Sangon Biotech (Shanghai, China). cDNA was obtained using HiScript® III-RT SuperMix for qPCR with gDNA wiper (Vazyme Biotech Co., Ltd). These cDNA samples were used as templates for real-time PCR analysis (qRT-PCR) with the specific primer sets listed in Table 1. The qRT-PCRs were performed using ChamQ™ Universal SYBR^®^qPCR Master Mix (Vazyme Biotech Co., Ltd) and ABI7500 Real-Time PCR system. The qPCRs proceeded as follows: initial denaturation at 94 °C for 1 min, followed by 40 cycles of denaturation at 94 °C for 10 s, annealing at 62 °C for 30 s, and elongation at 72 °C for 20 s. Specific amplification was confirmed by analysis of melting curves from 65 °C to 95 °C. Gene expressions were normalized to that of the β-actin gene (∆C_T_ method). The fold differences in gene expression between the transformants and the control strains CGMCC7326 were calculated by the 2^−ΔΔCT^ method. All samples were analyzed in triplicate.

### Analytical methods

Sugars and polyols were quantified by HPLC using a refractive index detector (Shodex RI101) and a Shodex SP0810 ion exclusion column (300 × 8 mm). Elution was performed at 70 °C using pure water at a flow rate of 1 mL/min. The mass yield of erythritol (*Y*_ery_) was expressed in g/g from glucose and was calculated from the equation *Y*_ery_ = *P*/*S*. The volumetric productivity (*Q*_ery_) was expressed in g/L·h and was calculated from *Q*_ery_ = *P*/*V* · *t*, where *P* is the amount of erythritol in the culture liquid at the end of fermentation (g); *S* is the total amount of glucose consumed (g); *V* is the initial volume of culture liquid (L), and *t* is the culture time (h). Glucose consumption rate (*R*_glu_) was calculated as the amount of glucose consumed per hour and per liter of culture medium.

## Supplementary information

**Additional file 1.**

## Data Availability

The authors promise the availability of data and material.
