# Correction to: Metabolic engineering of *Yarrowia lipolytica* for thermoresistance and enhanced erythritol productivity

**DOI:** 10.1186/s13068-020-01827-4

**Published:** 2020-11-17

**Authors:** Nan Wang, Ping Chi, Yawen Zou, Yirong Xu, Shuo Xu, Muhammad Bilal, Patrick Fickers, Hairong Cheng

**Affiliations:** 1 State Key Laboratory of Microbial Metabolism, and School of Life Sciences and Biotechnology, Shanghai Jiao Tong University, Shanghai, China; 2 School of Life Science and Food Engineering, Huaiyin Institute of Technology, Huaian, 223003 China; 3 Microbial Process and Interaction, TERRA Teaching and Research Centre, University of Liege – Gembloux Agro-Bio Tech, Gembloux, Belgium

## Correction to: Biotechnol Biofuels (2020) 13:176 https://doi.org/10.1186/s13068-020-01815-8

Following publication of the original article [1], the authors identified an error in the author name of Muhammad Bilal.

The incorrect author name is: M. Bilal.

The correct author name is: Muhammad Bilal.

The author group has been updated above and the original article [1] has been corrected.
