# Supplementary material for: Metabolic engineering of Yarrowia lipolytica for thermoresistance and enhanced erythritol productivity
Source: Biotechnol Biofuels. 2020 Oct 20;13:176. doi: 10.1186/s13068-020-01815-8 (PMC7576711; doi:10.1186/s13068-020-01815-8)

**Supplemental sequence data**

**Sequence 1: *Ku70* gene knockout cassette vector sequence**

1-167: pUC19 derived vector sequence

168-1613: upstream sequence of Ku70 gene

1614-1647: loxP sequence

1648-1759: minimal promoter of Leu2 gene

1760-2785: hph gene

2786-2990: terminator sequence

2991-3024: loxP sequence

3024-4518: downstream sequence of Ku70 gene

4519-6458: pUC19 derived vector sequence

**The *Ku70* gene knockout cassette fragment from 168 bp to 4518 bp was synthesized in vitro.**

1 AAAAATAAAC AAATAGGGGT TCCGCGCACA TTTCCCCGAA AAGTGCCACC TGACGTCTAA

61 GAAACCATTA TTATCATGAC ATTAACCTAT AAAAATAGGC GTATCACGAG GCCCTTTCGT

121 TGTAAAACGA CGGCCAGTCG TCTCGATCCG CAGTGTCTTG CGTCTCTGAA TTCATGCCGT

181 TTCTGTTGGT TCTACCGTCT ATGCCGTCCA GGTAAGCTAT GCTGGTTTGC GATACGCGAC

241 AGGTGGTAAG GTAGGAAATG AATGCATTTG CTTGAGTGGT GCAAGTGATA GATGAGATGG

301 AATGAATATA CCACTTGTAC TACTGTCACT GGTGTAGTTG CACGATATGC ACATTGGAGA

361 TGTTGGAGCA GATGGCTTGT CTATCTCTAC AAGTACAACT ACTTGTAGAT GCTGTAGAAG

421 ATTTAACAGA TATCAATGAA TTAAGTCTCC GTGTTACCAT CTTAGATATG AACACTAAAT

481 ATGCCAAGTT CTCTTTCCCC TACATGATTC ATAGTACTTG CCCAAAGACG AGGAGACCTT

541 CTTACGACAT ATATCTTACG ACATATACAT CAATAGACCA TTCATGACAA GAAAGAAGTA

601 CTCGAACATA GGCCCATAAA GTACGAGTAC AGTACACTAC ACTACACTAC TTGTACCATT

661 CTACCCGGGG TCTGCCGGCT TGTACACACC GACAGTACTC GTACTCTCCC ACGGATGCTC

721 CGGCTGCCGA CATCAACACG ATCTCAAAAG CGCATACTGA GCTTCCTTTC CTAGCTCTTC

781 CTTCCTTCAA CTCGATAAAT ACATTGGATA TATACATGTG TGGCGACTGT CGACTTGATG

841 TTTAGAGTGT CCAGATCCGC AAGATCGGCT CGCACTTGTG TTGTGTTGTT TCAAATCAGC

901 CTGTCGTTTT GTGTCGTTTG AGATCATTCT GTCTCACTCT TAGGCTCGCT TAGAACCGAC

961 AACGGAGAAT CCGGGCTCGG TTTTTCGGTC GGCCTTGATC TGGGCCTTGG ACTTGTACTG

1021 GTCGGCCATC TCCACGTTGA CCAGCTCCTT GACCTTGTAG AGCTGACCGG CGATACCAGG

1081 AGACACCTTG TAGTACTTCT GGGAGCCGAC CTTGCCCAGA CCGAGGGTCT TGAGCACGTC

1141 ACGTGTTCTC CACGGCATTC GCAGGATAGA TCGGACCTGT GTGACTTTGT AGAACATGGC

1201 GTTTCAGGTG GTTGCGTGAG TGTGTAAAAT CGTGTCTTTC AGAAGTTACA AATTTCACCG

1261 CATTTAGAGT TTATGCAGAT GGGCGGTGTG TGGTTGGGAG TTCGATTTCC GTGCGTGCAT

1321 TTGATCTTGA TGAATTGGAT TTGTACATGG GGAAGAGCAC GTCAAGAACC GCCTACTGCA

1381 AACTCGTGAA TATTGAGATT ATTGAGGAAA TTCAAGGAAA ATTCAGATCA GATTTGAGAG

1441 CAAAGTCCAA CAATACTACA CAATCCCTTT CCTGTATTCT TCCACCATCG TCATCGTCGT

1501 CTGTCTTCTC TTCAGCTTTT TAATTTCACT CCCCACAAAC CCAAATTTAG CTGCATCATT

1561 CATCAACCTC CAATTATAAC TATACATCGC GACACGAACA CGAAACACGA ACCATAACTT

1621 CGTATAATGT ATGCTATACG AAGTTATCAC GGGCAAAAGT GCGTATATAT ACAAGAGCGT

1681 TTGCCAGCCA CAGATTTTCA CTCCACACAC CACATCACAC ATACAACCAC ACACATCCAC

1741 AATGGAACCC GAAACTAAGA TGAAAAAGCC TGAACTCACC GCGACGTCTG TCGAGAAGTT

1801 TCTGATCGAA AAGTTCGACA GCGTCTCCGA CCTGATGCAG CTCTCGGAGG GCGAAGAATC

1861 TCGTGCTTTC AGCTTCGATG TAGGAGGGCG TGGATATGTC CTGCGGGTAA ATAGCTGCGC

1921 CGATGGTTTC TACAAAGATC GTTATGTTTA TCGGCACTTT GCATCGGCCG CGCTCCCGAT

1981 TCCGGAAGTG CTTGACATTG GGGAGTTCAG CGAGAGCCTG ACCTATTGCA TCTCCCGCCG

2041 TGCACAGGGT GTCACGTTGC AAGACCTGCC TGAAACCGAA CTGCCCGCTG TTCTACAGCC

2101 GGTCGCGGAG GCAATGGATG CGATCGCTGC GGCCGATCTT AGCCAGACGA GCGGGTTCGG

2161 CCCATTCGGA CCGCAAGGAA TCGGTCAATA CACTACATGG CGTGATTTCA TTTGCGCGAT

2221 TGCTGATCCC CATGTGTATC ACTGGCAAAC TGTGATGGAC GACACCGTCA GTGCGTCCGT

2281 CGCGCAGGCT CTCGATGAGC TGATGCTTTG GGCCGAGGAC TGCCCCGAAG TCCGGCACCT

2341 CGTGCACGCG GATTTCGGCT CCAACAATGT CCTGACGGAC AATGGCCGCA TAACAGCGGT

2401 CATTGACTGG AGCGAGGCGA TGTTCGGGGA TTCCCAATAC GAGGTCGCCA ACATCTTCTT

2461 CTGGAGGCCG TGGTTGGCTT GTATGGAGCA GCAGACGCGC TACTTCGAGC GGAGGCATCC

2521 GGAGCTTGCA GGATCGCCGC GACTCCGGGC GTATATGCTC CGCATTGGTC TTGACCAACT

2581 CTATCAGAGC TTGGTTGACG GCAATTTCGA TGATGCAGCT TGGGCGCAGG GTCGATGCGA

2641 CGCAATCGTC CGATCCGGAG CCGGGACTGT CGGGCGTACA CAAATCGCCC GCAGAAGCGC

2701 GGCCGTCTGG ACCGATGGCT GTGTAGAAGT ACTCGCCGAT AGTGGAAACC GACGCCCCAG

2761 CACTCGTCCG AGGGCAAAGG AATAGGACTC TATAAAAAGG GCCCTGCCCT GCTAATGAAA

2821 TGATGATTTA TAATTTACCG GTGTAGCAAC CTTGACTAGA AGAAGCAGAT TGGGTGTGTT

2881 TGTAGTGGAG GACAGTGGTA CGTTTTGGAA ACAGTCTTCT TGAAAGTGTC TTGTCTACAG

2941 TATATTCACT CATAACCTCA ATAGCCAAGG GTGTAGTCGG TTTATTAAAG ATAACTTCGT

3001 ATAATGTATG CTATACGAAG TTATCATGCA CTGGTATGAT CTGCAAGCAC TGTATAAATA

3061 ACAGTTGCTG CTTTGTCTCC AGACAGCAAT CCAGAGGAAA ATAGTGGAAA TAAAATCTCC

3121 CAATTACACA GGAAGATTCC ATAAATCAAT AAATATTACT ATTTAACCGC TTTTCCAAGA

3181 TATACGCAAA CTAAGCCTTC TCAGTCTTCT CGTCGGTGAC CTCAGTGATC TTATCCTTCT

3241 TCTCCTCGGC CTGCTTGGCC AGCTCCTGCT CCTTGAGATC GGCAAGGCGT CGGGCCTCAG

3301 CGTGAATATC GGACGCCTCC TTGGCGGCCT CAGAGTAGTA CTCCCGGATC TTTCGGCCGT

3361 AGGAAGTGTT AAGAGCATCC TCCCAGTACT TCTGGAGTCG GGCAGTGATG CCGTAGGCCT

3421 GGTCGGTGGC CTGGGCCTTG TCAACCACGT GGTACTTCTT GTCCAGGTCG GTCAGGTAGT

3481 TTGTGAACTT GGCAGTGATT CCGTGCTCCT TGTCGTAGTC AATGGCTCGC TCCAGAACCT

3541 TGTCGGACAG AACGTAACCG TTGGAGAGCA TCTCGGCGAG AATGGCGGCT CGAGGCTTGA

3601 GCTCCTGGGA GATGGACTCG GGGGTTCGAG CACCGTCGGC AGCGTCGTCA TTGGAGTCGG

3661 CAGTGGAGGG CTTCTTTTCG TCCTGGGTCT TGGAGTCGCT CTCAACAGTC TCAGTGGCTC

3721 GGGGCTCATC CAGCTTCTTG AGGGTGGCGT CGTCGACCTC AATGGACACC TTGGAGTCGC

3781 CAATCTGTCC GTCGGCGATC AGGAGCGCAG TTCGAACTGC CTCCAGGTTG GCAAAGGTGA

3841 CTGTTGCGGT GTGGGTCTCC TCGTCAATGG CCACGGACTT GACCTTGCCA CAGAAGGAAA

3901 AGTACTTCTT GATTTCGGCC TTGGAGGTGG TTGGCGAGAC GCCCTTGGCG GTGATGTTGT

3961 AGGACATGTT GTGTGTGATA GTGTCTGGCT AATGTGGATG GTAGGGGAAC TGAGAAGGGT

4021 GGTTTGCAAG GCTTATATAG ATAAGATAGA GTGGGAATAC TAGGTTTCAA ATTGACTTTG

4081 CTGTAATGTA AGGTTCTTTT TTAACATTCG CCGCCTGGCA TGTGCTATAG TCTGACTGGG

4141 ACTTGTTTTG GATAGCTGGA CTGGAACGAG AGGGGCTTGG AGGGTTCCAA ACGACTGTAA

4201 ATCCGCCCAA CGGCCGATAA GTTCAGGTTG AAACCCCTCC AAACCGATTT TTTCGCGTTT

4261 TTTCGCCGCT TTTTCACCCC TTCTGACTCC TCCTGCACAC CCTGTCTGGA TCAGCAGACG

4321 GAGATCGACA GGATACGATG GAGAGGGGTT CCTGTGTTGA GGAGAGCCAA ATTGAAACAA

4381 CAAAAACGCG ACACCTTTCA TTAGTGCCAC AAGATATGGA CAAACACGGG CAAAACCTGA

4441 CTCAGTAGCA CCGCCACTAA GAGCACGCAC AAACAACCAG TGGCTAAACA TCAGCAACTT

4501 TCACATGCTG CAGTTGTGCA GGTTGCGGCC GCAGAGACGG AGTCACTGCC AACCGAGACG

4561 GTCATAGCTG TTTCCTGTGT GCCGCTTCCT CGCTCACTGA CTCGCTGCGC TCGGTCGTTC

4621 GGCTGCGGCG AGCGGTATCA GCTCACTCAA AGGCGGTAAT ACGGTTACCC ACAGAATCAG

4681 GGGATAACGC AGGAAAGAAC ATGTGAGCAA AAGGCCAGCA AAAGGCCAGG AACCGTAAAA

4741 AGGCCGCGTT GCTGGCGTTT TTCCATAGGC TCCGCCCCCC TGACGAGCAT CACAAAAATC

4801 GACGCTCAAG TCAGAGGTGG CGAAACCCGA CAGGACTATA AAGATACCAG GCGTTTCCCC

4861 CTGGAAGCTC CCTCGTGCGC TCTCCTGTTC CGACCCTGCC GCTTACCGGA TACCTGTCCG

4921 CCTTTCTCCC TTCGGGAAGC GTGGCGCTTT CTCAATGCTC ACGCTGTAGG TATCTCAGTT

4981 CGGTGTAGGT CGTTCGCTCC AAGCTGGGCT GTGTGCACGA ACCCCCCGTT CAGCCCGACC

5041 GCTGCGCCTT ATCCGGTAAC TATCGTCTTG AGTCCAACCC GGTAAGACAC GACTTATCGC

5101 CACTGGCAGC AGCCACTGGT AACAGGATTA GCAGAGCGAG GTATGTAGGC GGTGCTACAG

5161 AGTTCTTGAA GTGGTGGCCT AACTACGGCT ACACTAGAAG GACAGTATTT GGTATCTGCG

5221 CTCTGCTGAA GCCAGTTACC TTCGGAAAAA GAGTTGGTAG CTCTTGATCC GGCAAACAAA

5281 CCACCGCTGG TAGCGGTGGT TTTTTTGTTT GCAAGCAGCA GATTACGCGC AGAAAAAAAG

5341 GATCTCAAGA AGATCCTTTG ATCTTTTCTA CGGGGTCTGA CGCTCAGTGG AACGAAAACT

5401 CACGTTAAGG GATTTTGGTC ATGAGATTAT CAAAAAGGAT CTTCACCTAG ATCCTTTTAA

5461 ATTAAAAATG AAGTTTTAAA TCAATCTAAA GTATATATGA GTAAACTTGG TCTGACAGTT

5521 ACCAATGCTT AATCAGTGAG GCACCTATCT CAGCGATCTG TCTATTTCGT TCATCCATAG

5581 TTGCCTGACT CCCCGTCGTG TAGATAACTA CGATACGGGA GGGCTTACCA TCTGGCCCCA

5641 GTGCTGCAAT AATACCGCGG GACCCACGCT CACCGGCTCC AGATTTATCA GCAATAAACC

5701 AGCCAGCCGG AAGGGCCGAG CGCAGAAGTG GTCCTGCAAC TTTATCCGCC TCCATCCAGT

5761 CTATTAATTG TTGCCGGGAA GCTAGAGTAA GTAGTTCGCC AGTTAATAGT TTGCGCAACG

5821 TTGTTGCCAT CGCTACAGGC ATCGTGGTGT CACGCTCGTC GTTTGGTATG GCTTCATTCA

5881 GCTCCGGTTC CCAACGATCA AGGCGAGTTA CATGATCCCC CATGTTGTGC AAAAAAGCGG

5941 TTAGCTCCTT CGGTCCTCCG ATCGTTGTCA GAAGTAAGTT GGCCGCCGTG TTATCACTCA

6001 TGGTTATGGC AGCACTACAT AATTCTCTTA CTGTCATGCC ATCCGTAAGA TGCTTTTCTG

6061 TGACTGGTGA GTACTCAACC AAGTCATTCT GAGAATAGTG TATGCGGCGA CCGAGTTGCT

6121 CTTGCCCGGC GTCAATACGG GATAATACCG CGCCACATAG CAGAACTTTA AAAGTGCTCA

6181 TCATTGGAAA ACGTTCTTCG GGGCGAAAAC TCTCAAGGAT CTTACCGCTG TTGAGATCCA

6241 GTTCGATGTA ACCCACTCGT GCACCCAACT GATCTTCAGC ATCTTTTACT TTCACCAGCG

6301 TTTCTGGGTG AGCAAAAACA GGAAGGCAAA ATGCCGCAAA AAAGGGAATA AGGGCGACAC

6361 GGAAATGTTG AATACTCATA CTCTTCCTTT TTCAATATTA TTGAAGCATT TATCAGGGTT

6421 ATTGTCTCAT GAGCGGATAC ATATTTGAAT GTATTTAG


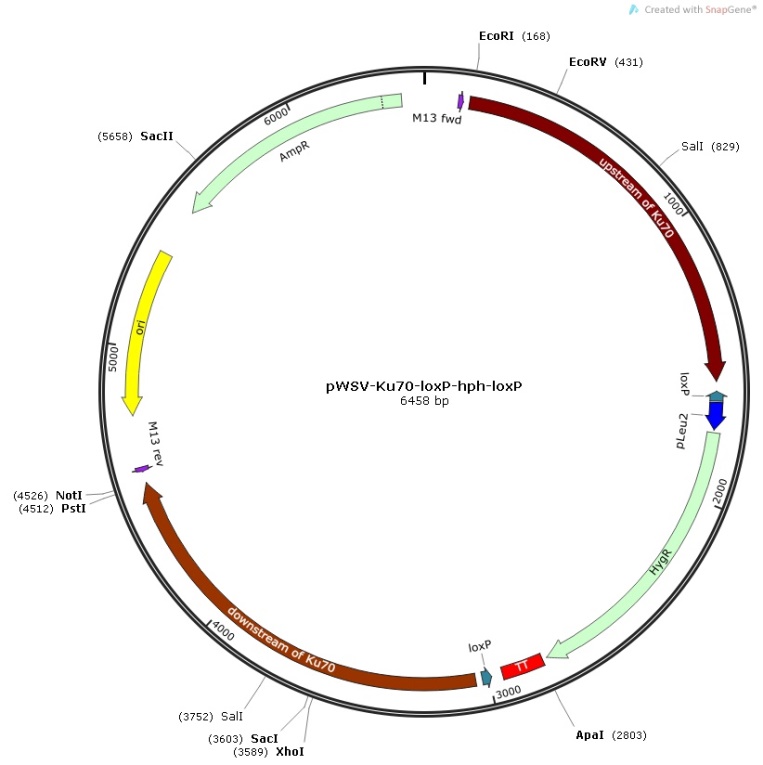


**Sequence 2: sequence of fragment *EcoR*I-hp4d-621XDH-TT-hp4d-Cre-*Sal*I in pUB4-XDH**

1-6： *EcoR*I

7-539：hp4d promoter

540-1329: 621XDH

1330-1558: terminator

1559-3556: Cre expression cassette

3557-3563: *Sal*I

1 GAATTCCTGA GGTGTCTCAC AAGTGCCGTG CAGTCCCGCC CCCACTTGCT TCTCTTTGTG

61 TGTAGTGTAC GTACATTATC GAGACCGTTG TTCCCGCCCA CCTCGATCCG GCTGAGGTGT

121 CTCACAAGTG CCGTGCAGTC CCGCCCCCAC TTGCTTCTCT TTGTGTGTAG TGTACGTACA

181 TTATCGAGAC CGTTGTTCCC GCCCACCTCG ATCCGGCTGA GGTGTCTCAC AAGTGCCGTG

241 CAGTCCCGCC CCCACTTGCT TCTCTTTGTG TGTAGTGTAC GTACATTATC GAGACCGTTG

301 TTCCCGCCCA CCTCGATCCG GCTGAGGTGT CTCACAAGTG CCGTGCAGTC CCGCCCCCAC

361 TTGCTTCTCT TTGTGTGTAG TGTACGTACA TTATCGAGAC CGTTGTTCCC GCCCACCTCG

421 ATCCGGCACG GGCAAAAGTG CGTATATATA CAAGAGCGTT TGCCAGCCAC AGATTTTCAC

481 TCCACACACC ACATCACACA TACAACCACA CACATCCACA ATGGAACCCG AAACTAAGAT

541 GTCGAAGAAG TTTAACGGTA AGGTCTGTCT GGTCACCGGC GCTGGTGGCA ACATCGGTCT

601 TGCTACCGCC CTCCGTCTGG CCGAAGAGGG CACGGCCATC GCCCTTCTGG ACATGAACCG

661 AGAGGCTCTG GAAAAGGCTG AAGCCTCCGT CCGTGAAAAG GGCGTCGAAG CCCGATCCTA

721 TGTCTGTGAC GTCACGTCCG AAGAGGCCGT GATCGGTACG GTGGATAGCG TGGTCCGGGA

781 CTTCGGTAAG ATCGACTTCC TGTTCAACAA CGCCGGCTAT CAGGGAGCCT TCGCCCCCGT

841 GCAGGACTAC CCGTCCGACG ATTTCGCCCG AGTGCTGACG ATCAACGTCA CTGGTGCCTT

901 CCACGTCCTC AAGGCCGTTT CGCGACAGAT GATCACGCAG AACTACGGTC GAATCGTCAA

961 CACCGCCAGC ATGGCCGGTG TGAAGGGACC GCCAAACATG GCCGCCTATG GTACGTCCAA

1021 GGGAGCCATC ATCGCCCTGA CCGAAACGGC CGCTCTTGAC CTTGCCCCCT ACAACATCCG

1081 TGTGAACGCC ATCAGCCCCG GTTACATGGG TCCCGGTTTC ATGTGGGAGC GTCAGGTCGA

1141 GCTTCAGGCC AAGGTCGGAA GCCAGTATTT CTCCACCGAT CCCAAGGTCG TGGCCCAGCA

1201 GATGATCGGC AGCGTTCCGA TGCGACGATA TGGCGACATC AACGAGATTC CGGGCGTGGT

1261 GGCTTTCCTG CTGGGTGATG ATTCCAGCTT CATGACGGGT GTGAACCTGC CGATTGCTGG

1321 CGGTTGAGCA ATTAACAGAT AGTTTGCCGG TGATAATTCT CTTAACCTCC CACACTCCTT

1381 TGACATAACG ATTTATGTAA CGAAACTGAA ATTTGACCAG ATATTGTTGT AAATAGAAAA

1441 TCTGGCTTGT AGGTGGCAAA ATCCCGTCTT TGTTCGTCGG TTCCCTCTGT GACTGCTCGT

1501 CGTCCCTTTG TGTTCGACTG TCGTGTTTTG TTTTCCGTGC GTGCGCAAGT GAGATGCCCG

1561 TGTTCGAATT GGGTAGTCGC ACGGAGCATG CTGAGGTGTC TCACAAGTGC CGTGCAGTCC

1621 CGCCCCCACT TGCTTCTCTT TGTGTGTAGT GTACGTACAT TATCGAGACC GTTGTTCCCG

1681 CCCACCTCGA TCCGGCATGC TGAGGTGTCT CACAAGTGCC GTGCAGTCCC GCCCCCACTT

1741 GCTTCTCTTT GTGTGTAGTG TACGTACATT ATCGAGACCG TTGTTCCCGC CCACCTCGAT

1801 CCGGCATGCT GAGGTGTCTC ACAAGTGCCG TGCAGTCCCG CCCCCACTTG CTTCTCTTTG

1861 TGTGTAGTGT ACGTACATTA TCGAGACCGT TGTTCCCGCC CACCTCGATC CGGCATGCTG

1921 AGGTGTCTCA CAAGTGCCGT GCAGTCCCGC CCCCACTTGC TTCTCTTTGT GTGTAGTGTA

1981 CGTACATTAT CGAGACCGTT GTTCCCGCCC ACCTCGATCC GGCATGCACT GATCACGGGC

2041 AAAAGTGCGT ATATATACAA GAGCGTTTGC CAGCCACAGA TTTTCACTCC ACACACCACA

2101 TCACACATAC AACCACACAC ATCCACGGGC TGCAGGAATT CGATATCAAG CTTATCGATA

2161 CCGTCGAGGG GCAGAGCCGA TCCTGTACAC TTTACTTAAA ACCATTATCT GAGTGTTAAA

2221 TGTCCAATTT ACTGACCGTA CACCAAAATT TGCCTGCATT ACCGGTCGAT GCAACGAGTG

2281 ATGAGGTTCG CAAGAACCTG ATGGACATGT TCAGGGATCG CCAGGCGTTT TCTGAGCATA

2341 CCTGGAAAAT GCTTCTGTCC GTTTGCCGGT CGTGGGCGGC ATGGTGCAAG TTGAATAACC

2401 GGAAATGGTT TCCCGCAGAA CCTGAAGATG TTCGCGATTA TCTTCTATAT CTTCAGGCGC

2461 GCGGTCTGGC AGTAAAAACT ATCCAGCAAC ATTTGGGCCA GCTAAACATG CTTCATCGTC

2521 GGTCCGGGCT GCCACGACCA AGTGACAGCA ATGCTGTTTC ACTGGTTATG CGGCGGATCC

2581 GAAAAGAAAA CGTTGATGCC GGTGAACGTG CAAAACAGGC TCTAGCGTTC GAACGCACTG

2641 ATTTCGACCA GGTTCGTTCA CTCATGGAAA ATAGCGATCG CTGCCAGGAT ATACGTAATC

2701 TGGCATTTCT GGGGATTGCT TATAACACCC TGTTACGTAT AGCCGAAATT GCCAGGATCA

2761 GGGTTAAAGA TATCTCACGT ACTGACGGTG GGAGAATGTT AATCCATATT GGCAGAACGA

2821 AAACGCTGGT TAGCACCGCA GGTGTAGAGA AGGCACTTAG CCTGGGGGTA ACTAAACTGG

2881 TCGAGCGATG GATTTCCGTC TCTGGTGTAG CTGATGATCC GAATAACTAC CTGTTTTGCC

2941 GGGTCAGAAA AAATGGTGTT GCCGCGCCAT CTGCCACCAG CCAGCTATCA ACTCGCGCCC

3001 TGGAAGGGAT TTTTGAAGCA ACTCATCGAT TGATTTACGG CGCTAAGGAT GACTCTGGTC

3061 AGAGATACCT GGCCTGGTCT GGACACAGTG CCCGTGTCGG AGCCGCGCGA GATATGGCCC

3121 GCGCTGGAGT TTCAATACCG GAGATCATGC AAGCTGGTGG CTGGACCAAT GTAAATATTG

3181 TCATGAACTA TATCCGTACC CTGGATAGTG AAACAGGGGC AATGGTGCGC CTGCTGGAAG

3241 ATGGCGATTA GCCATTAACG CGTAAATGAT TGCTATAATT ATTTGATATT TATGGTGACA

3301 TATGAGAAAG GATTTCAACA TCGACGGAAA ATATGTAGTG CTGTCTGTAA GCACTAATAT

3361 TCAGTCGCCA GCCGTCATTG TCACTGTAAA GCTGAGCGAT AGAATGCCTG ATATTGACTC

3421 AATATCCGTT GCGTTTCCTG TCAAAAGTAT GCGTAGTGCT GAACATTTCG TGATGAATGC

3481 CACCGAGGAA GAAGCACGGC GCGGTTTTGC NTAAAGTGAT GTCTGAGTTT GGCGAACTCT

3541 TGGGTAAGGT TGGAATTGTC GAC


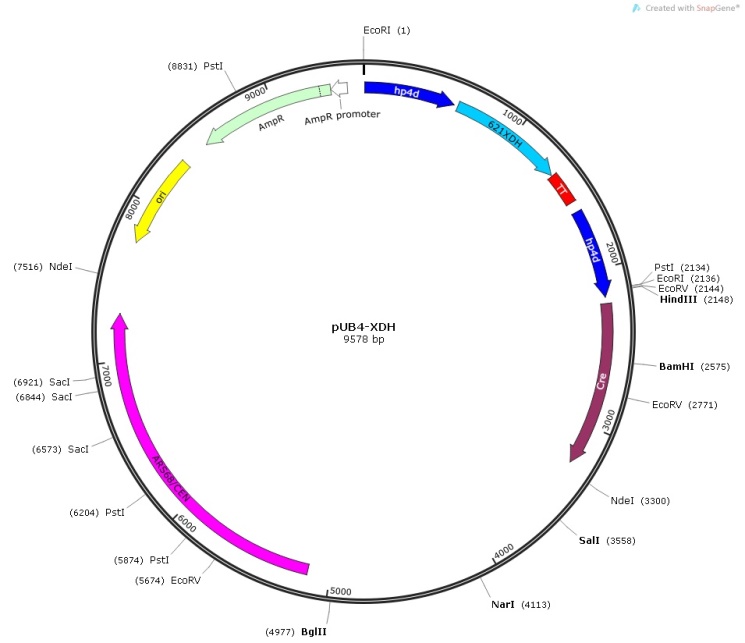


**Sequence 3: The *ArDH1* gene knockout vector**

1-167: pUC19 derived vector sequence

168-1641: upstream sequence of ArDH1 gene

1642-1675: loxP sequence

1676-1787: minimal promoter of Leu2 gene

1788-2813: hph gene

2814-3018: terminator sequence

3019-3052: loxP sequence

3053-4552: downstream sequence of ArDH1 gene

4553-6486: pUC19 derived vector sequence

**The *ArDH1* gene knockout cassette fragment from 168 bp to 4552 bp was synthesized in vitro.**

1 AAAAATAAAC AAATAGGGGT TCCGCGCACA TTTCCCCGAA AAGTGCCACC TGACGTCTAA

61 GAAACCATTA TTATCATGAC ATTAACCTAT AAAAATAGGC GTATCACGAG GCCCTTTCGT

121 TGTAAAACGA CGGCCAGTCG TCTCGATCCG CAGTGTCTTG CGTCTCTGAA TTCTATACAA

181 GGATCTGATC CCCCAGACCC CCGTTCTTGC CCGGTACTTT AATGGTTACC GAGTTCTTGA

241 CCGACTTGTC AACACCAACC CCAACCTTGC TGGAGCTATC CCCACCAAGG AGATGAACGA

301 CGAGTGTGCC GATGTCGGTG GAACTGGTGC CTGGGACGTT GGTGGAGTGT GGCGATGTCT

361 GTTCATGAAG AAGCAGGACC CCCAGCACAG CCAGAACGAG AAGATGTTCC TCAAGTACGA

421 CGACTTCATC GACTGGCGAA AGCAGATGAT TGACCAGATC CGAGAGAAGG AGGTGGCCTC

481 GGACAAGGAG CGACGCGACA AGCTGCGTGC CGCCCGAAGC AAGTGGGAGG AGGACCGAAA

541 GTCGAGAATG ATCACCGAGA AGGAGGCCAA GGATCAGGGT CTGACCGTCA CCCGACATTC

601 TCACCAGACT GAGATGTACT ACGACCACGC TAGCCGAGAG TGGGTCAAGC AGGACACCAT

661 TCGAAAGTTC TACAACAACG GCACCGAGTC CGAGATCGTC AAGGAGTCTC GGGATCCTAT

721 TGGCAAGTAG ACATTGAGGT CTTCTTTTAG TAAATAGTTC TGTCCATTTA CTGGACATTT

781 TAACATAGCA ATTTATTCTC ATACTGAATC ACCAGATTTT AGGTTCCCTT ATCGTTATCG

841 TATGTGGAGA GACAGCACCA TCCGTATAAA AATGAGGCCT TAAGAAGGAT GAATCTACTG

901 TAGTTTGGAA GATGTGGTCG CGTGATTGGT GTCTTTTTGC CCCTTGTGTG TTTCGTCATA

961 TAGGTACTTG TACGTCACTC TTCAGAGAGA ACTCTCCACG GAGTCTTATA TCCATCCTAA

1021 TTACATAATG TAAATATGCA CATTTCTCCA CAAGACGCGT TTAAAAAGGG TGGCGAATCG

1081 CGAAACAATC GCTTTTACGT ATCCAGTATT CAATTTCGTT AAATATAATG GTCTTATATA

1141 TATACATATT CCGAGGAAAA CAGCGATATC AGGTGACGTT ATCCATCACT CATCGTCTTC

1201 GTCGTCCTCC TGCTCGGCAA GCTTTCGTGC AGCCGCCAAG TCAATCTTCA TGTTGTAGTG

1261 CTGGTTTCGT TTCTTGAGAA ACTCTTCATG TTGCTGTTGA TCCTCTTCTC TCAGCTGTTC

1321 CCGCTCGTCA ATCTCGTGGT CGTCGTCGTC TTCCTCCACC GAGGAGTCCT TGATGATTCG

1381 TTCCTTCTCC AGTGTTCCGT CTTCGTCAAA CTCGGGCTCT CCAAGAGAAA ATCCCGTCGC

1441 CTCTTCATCG TCCTCAGCGT AGTACTCGTT GTCTCCCAGA GTGCCGGCAT ACGGAGTCTT

1501 GGGCTCGTCA ATCTTCATGT ATGTTCCCTT ATCCTGCTCA TGGAGGTAGA TTCCAGCCTC

1561 GTCCCACGAA ATGTCGCTGG GCTGACCTCC GTTGGGGCCA TTGACGTTGG AAGGGAACTC

1621 CGTGGTGCCC GACGACGCCT CATAACTTCG TATAATGTAT GCTATACGAA GTTATCACGG

1681 GCAAAAGTGC GTATATATAC AAGAGCGTTT GCCAGCCACA GATTTTCACT CCACACACCA

1741 CATCACACAT ACAACCACAC ACATCCACAA TGGAACCCGA AACTAAGATG AAAAAGCCTG

1801 AACTCACCGC GACGTCTGTC GAGAAGTTTC TGATCGAAAA GTTCGACAGC GTCTCCGACC

1861 TGATGCAGCT CTCGGAGGGC GAAGAATCTC GTGCTTTCAG CTTCGATGTA GGAGGGCGTG

1921 GATATGTCCT GCGGGTAAAT AGCTGCGCCG ATGGTTTCTA CAAAGATCGT TATGTTTATC

1981 GGCACTTTGC ATCGGCCGCG CTCCCGATTC CGGAAGTGCT TGACATTGGG GAGTTCAGCG

2041 AGAGCCTGAC CTATTGCATC TCCCGCCGTG CACAGGGTGT CACGTTGCAA GACCTGCCTG

2101 AAACCGAACT GCCCGCTGTT CTACAGCCGG TCGCGGAGGC AATGGATGCG ATCGCTGCGG

2161 CCGATCTTAG CCAGACGAGC GGGTTCGGCC CATTCGGACC GCAAGGAATC GGTCAATACA

2221 CTACATGGCG TGATTTCATT TGCGCGATTG CTGATCCCCA TGTGTATCAC TGGCAAACTG

2281 TGATGGACGA CACCGTCAGT GCGTCCGTCG CGCAGGCTCT CGATGAGCTG ATGCTTTGGG

2341 CCGAGGACTG CCCCGAAGTC CGGCACCTCG TGCACGCGGA TTTCGGCTCC AACAATGTCC

2401 TGACGGACAA TGGCCGCATA ACAGCGGTCA TTGACTGGAG CGAGGCGATG TTCGGGGATT

2461 CCCAATACGA GGTCGCCAAC ATCTTCTTCT GGAGGCCGTG GTTGGCTTGT ATGGAGCAGC

2521 AGACGCGCTA CTTCGAGCGG AGGCATCCGG AGCTTGCAGG ATCGCCGCGA CTCCGGGCGT

2581 ATATGCTCCG CATTGGTCTT GACCAACTCT ATCAGAGCTT GGTTGACGGC AATTTCGATG

2641 ATGCAGCTTG GGCGCAGGGT CGATGCGACG CAATCGTCCG ATCCGGAGCC GGGACTGTCG

2701 GGCGTACACA AATCGCCCGC AGAAGCGCGG CCGTCTGGAC CGATGGCTGT GTAGAAGTAC

2761 TCGCCGATAG TGGAAACCGA CGCCCCAGCA CTCGTCCGAG GGCAAAGGAA TAGGACTCTA

2821 TAAAAAGGGC CCTGCCCTGC TAATGAAATG ATGATTTATA ATTTACCGGT GTAGCAACCT

2881 TGACTAGAAG AAGCAGATTG GGTGTGTTTG TAGTGGAGGA CAGTGGTACG TTTTGGAAAC

2941 AGTCTTCTTG AAAGTGTCTT GTCTACAGTA TATTCACTCA TAACCTCAAT AGCCAAGGGT

3001 GTAGTCGGTT TATTAAAGAT AACTTCGTAT AATGTATGCT ATACGAAGTT ATAGGATGAA

3061 GGAGGTGAAC ATGACAACCA GGAGGATTCG GCCTGCCAGG AGCACGTACT TGGACCGGTC

3121 CTTGTCGTCA ATAGAGGGGA GACCAGGGAG ACCTCGCTTG GACTTGTCCT TGACAAAGGC

3181 GTCGTTAAGA GCGATGAAAA GACCGCCAAT GACGGACAGG TTTCGCAGAA TGAAGCCAAA

3241 GTCAAAGATG AGGCCGTAGG CCAGAGCCTG GGTGACAATG ACTCCGACCA GCAGACCGCA

3301 ACCGACCTCA ATTCGCTTCT TGGCGGTGAC CATGAAGGAT CCGGCGATCA TGGCAACCAC

3361 GTTGAGCAGC AGAAAGATGA CGGTGATAAA CTTGGGGATG TGCTTGAAGT TGGTGATGTA

3421 GTAGACCTGG TCAGACCACT GTGTGAGGAT TCGGAGCGCG TCCTCCAGGA AAGTGACGAC

3481 AATGAGGAAC CGGCCGAGGG TGGGAAGATG GGGCTTGACC GGGCCCGAGT ACTTGTCCAG

3541 GAAGACCTCA ATCTTCTGGG TCACGGCCTT GACCTTGTCG GCAAAGTCGC CCTCTGTGAT

3601 TGTGGATTTG AGGTTGAAGT CGCGGTTCTG GGAGTAGGAC GAAAACTGTT GACCCTGGGG

3661 GGCTGCGAAT TGCGGCGCGT CGCTCTGGAC GGGTATCTGG TGGTATTTGG CGGTGCCTCG

3721 GATCGACATT GTTGTATGGT GTGGTGTTAA CACAGGAAGA TTGTGAAAGG TAGAATATGT

3781 AATCTTGTGT GTGCCAGGTG GTCTTTCGAG AGTTTGTTCA GATCAGCATG CCCACTTCGC

3841 TGCAGTAGCC AAAAACAGAT GTGTGGAATG GGAAGGGTGG GAAGCTGGGA CTAGGCAGGG

3901 TAGTTGTAAG TGGACGTTGG AGGGCTAATA TATCTCCGTT ATTCCGCCAT TGCGTGTCCT

3961 CTTATACAGA TTGGCCACTT TTACGGCGAT GACATCTCCC ATCTTGCTGT TCTCTTCGGT

4021 TAATTAATTA GGTCATCTCA AACTACAGTA TTAGCTACAG TCCACGTCAT GCGGCGGCAA

4081 GCTACTGTAT TTGGTGGGGT TGGGGGGGAA AGGATGTTTA TTACTGTAGA GCAGGCCTGG

4141 CGTAAGCTAC TGTATAATGG CTGAGGGACG CGAGCATGGG TCTACGACTC ATATGTAGGG

4201 CTAGATTGAC TTACGGGCTC AAGTGATAGG AAAGGGCTTG TATACGCAGT AAAACACACG

4261 TTAATAAATA ATTCAATTGA ATCAAACAAT ATAAGTACAG TACGGGTACC CCAGATCGTC

4321 TACTCTATCG GTTGTGTCTC TCGCAATACA GCGTCCCTTT TTCCACCTCA CGGTTTCACT

4381 TTTCACGCAC GTCCCCAAAC CTTTTTTAGC CGGGTCAAAC AATGACGTGG CATCTGAAGG

4441 CCAGCTGGCT GCACTGCGTG CACACGGGAG GTGTGAAAGA GAATGGCTTC GAATGGGGTA

4501 AAGTGAGGCC ATATGGGGCA GATGGGCATG TGCTACGAGA GCATGAGGCC GGGCGGCCGC

4561 AGAGACGGAG TCACTGCCAA CCGAGACGGT CATAGCTGTT TCCTGTGTGC CGCTTCCTCG

4621 CTCACTGACT CGCTGCGCTC GGTCGTTCGG CTGCGGCGAG CGGTATCAGC TCACTCAAAG

4681 GCGGTAATAC GGTTACCCAC AGAATCAGGG GATAACGCAG GAAAGAACAT GTGAGCAAAA

4741 GGCCAGCAAA AGGCCAGGAA CCGTAAAAAG GCCGCGTTGC TGGCGTTTTT CCATAGGCTC

4801 CGCCCCCCTG ACGAGCATCA CAAAAATCGA CGCTCAAGTC AGAGGTGGCG AAACCCGACA

4861 GGACTATAAA GATACCAGGC GTTTCCCCCT GGAAGCTCCC TCGTGCGCTC TCCTGTTCCG

4921 ACCCTGCCGC TTACCGGATA CCTGTCCGCC TTTCTCCCTT CGGGAAGCGT GGCGCTTTCT

4981 CAATGCTCAC GCTGTAGGTA TCTCAGTTCG GTGTAGGTCG TTCGCTCCAA GCTGGGCTGT

5041 GTGCACGAAC CCCCCGTTCA GCCCGACCGC TGCGCCTTAT CCGGTAACTA TCGTCTTGAG

5101 TCCAACCCGG TAAGACACGA CTTATCGCCA CTGGCAGCAG CCACTGGTAA CAGGATTAGC

5161 AGAGCGAGGT ATGTAGGCGG TGCTACAGAG TTCTTGAAGT GGTGGCCTAA CTACGGCTAC

5221 ACTAGAAGGA CAGTATTTGG TATCTGCGCT CTGCTGAAGC CAGTTACCTT CGGAAAAAGA

5281 GTTGGTAGCT CTTGATCCGG CAAACAAACC ACCGCTGGTA GCGGTGGTTT TTTTGTTTGC

5341 AAGCAGCAGA TTACGCGCAG AAAAAAAGGA TCTCAAGAAG ATCCTTTGAT CTTTTCTACG

5401 GGGTCTGACG CTCAGTGGAA CGAAAACTCA CGTTAAGGGA TTTTGGTCAT GAGATTATCA

5461 AAAAGGATCT TCACCTAGAT CCTTTTAAAT TAAAAATGAA GTTTTAAATC AATCTAAAGT

5521 ATATATGAGT AAACTTGGTC TGACAGTTAC CAATGCTTAA TCAGTGAGGC ACCTATCTCA

5581 GCGATCTGTC TATTTCGTTC ATCCATAGTT GCCTGACTCC CCGTCGTGTA GATAACTACG

5641 ATACGGGAGG GCTTACCATC TGGCCCCAGT GCTGCAATAA TACCGCGGGA CCCACGCTCA

5701 CCGGCTCCAG ATTTATCAGC AATAAACCAG CCAGCCGGAA GGGCCGAGCG CAGAAGTGGT

5761 CCTGCAACTT TATCCGCCTC CATCCAGTCT ATTAATTGTT GCCGGGAAGC TAGAGTAAGT

5821 AGTTCGCCAG TTAATAGTTT GCGCAACGTT GTTGCCATCG CTACAGGCAT CGTGGTGTCA

5881 CGCTCGTCGT TTGGTATGGC TTCATTCAGC TCCGGTTCCC AACGATCAAG GCGAGTTACA

5941 TGATCCCCCA TGTTGTGCAA AAAAGCGGTT AGCTCCTTCG GTCCTCCGAT CGTTGTCAGA

6001 AGTAAGTTGG CCGCCGTGTT ATCACTCATG GTTATGGCAG CACTACATAA TTCTCTTACT

6061 GTCATGCCAT CCGTAAGATG CTTTTCTGTG ACTGGTGAGT ACTCAACCAA GTCATTCTGA

6121 GAATAGTGTA TGCGGCGACC GAGTTGCTCT TGCCCGGCGT CAATACGGGA TAATACCGCG

6181 CCACATAGCA GAACTTTAAA AGTGCTCATC ATTGGAAAAC GTTCTTCGGG GCGAAAACTC

6241 TCAAGGATCT TACCGCTGTT GAGATCCAGT TCGATGTAAC CCACTCGTGC ACCCAACTGA

6301 TCTTCAGCAT CTTTTACTTT CACCAGCGTT TCTGGGTGAG CAAAAACAGG AAGGCAAAAT

6361 GCCGCAAAAA AGGGAATAAG GGCGACACGG AAATGTTGAA TACTCATACT CTTCCTTTTT

6421 CAATATTATT GAAGCATTTA TCAGGGTTAT TGTCTCATGA GCGGATACAT ATTTGAATGT

6481 ATTTAG


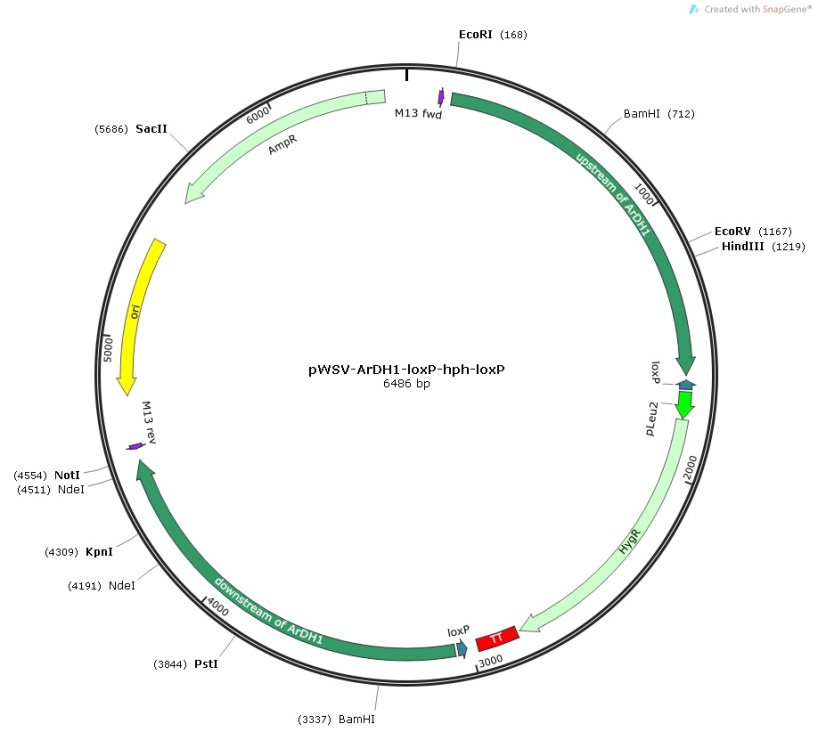


**Sequence 4: The *ArDH2* gene knockout vector**

1-167: pUC19 derived vector sequence

168-1733: upstream sequence of ArDH2 gene

1734-1768: loxP sequence

1769-1881: minimal promoter of Leu2 gene

1879-2905: hph gene

2906-3110: terminator sequence

3111-3144: loxP sequence

3145-4712: downstream sequence of ArDH2 gene

4713-6638: pUC19 derived vector sequence

**The *ArDH2* gene knockout cassette fragment from 168 bp to 4712 bp was synthesized in vitro.**

1 AAAAATAAAC AAATAGGGGT TCCGCGCACA TTTCCCCGAA AAGTGCCACC TGACGTCTAA

61 GAAACCATTA TTATCATGAC ATTAACCTAT AAAAATAGGC GTATCACGAG GCCCTTTCGT

121 TGTAAAACGA CGGCCAGTCG TCTCGATCCG CAGTGTCTTG CGTCTCTGAA TTCAATACCT

181 CCTCAGGTTT GGTCCCTTGA AAATGGGTGT TTAAGCATAA TGATTGTGGT TAACGATTCT

241 CGTGAAGGCA TCTTCGAGAA GTAGTGGGTG AATCCAAGCT TTCTCAAATT ATTCTTAGCC

301 TTACTGACAG GTCTACTGCT GGCGATCCGG TTTACTTCAA TCACCAACTA ATTATAGTTA

361 GCTCTTCCAC TTGGGACAAA AATACATTTT CAAATCAAAG TTGAGGTTGA AAGATCGGCA

421 GGTCGAGATT GGTGAGCGAT TATGAGGTGT GTTCCACTGA CGTTCCCAGA GCAAGTTCTG

481 TGGGCCGCCA TAAAACTGGC CTTTGTACAG TGCTACGTGT CTTCATCTCT AGGACCTGCA

541 GATTAGCACC ATTTTTAGCG TATCTTCTGG GGTGGCGTCG GGGCAGTGTT TGAGTCTTAA

601 CTCCGATAAT TCCGTGCCGG ATACATGATA CATGATGCGT TCTTTCTTCA ACTTTATGCG

661 AGTGAGTGAC GTCATGGAAA GACGGGCATT TCAGCTTAGT AAGCACGTTC GGGGGAAATG

721 CGGAGTAGAA AATGGAAGAA ACAAGAAATT GATGAAAACC ACTTTTGAGG ATGATTCCCA

781 ACGATCCCGG AAAAACGGAA AATCTGCAAA CTTTTCAAAA AACACCGTTA AAACTTTCCA

841 ATGGCTCCAA TGGCTTCCCA AATAAAGACA GAGGTAAATC CACCGTATTA GTCACCTTAC

901 CCTCGTTCTA TCGAGCCCAA ACACGGCCCA TTTAGCTTAG GATCATGGCC GATTGTGCGG

961 TACAGGGGAA AATATTTTCC ATACTCACAT CCCTACAACT CTAATGACCC GGCATGACGA

1021 ACTTGGTAAT CGATCCGGAT AATAAAGAGG AAAGATCAGG AGAATGAGGA CAGTGAAATT

1081 AGTGGTATGT GCGGGGTACA AGGGTCATTT GCGGATGCTA GAGGAGCGTG GGGTATTCCG

1141 ATTGGCTCGA TTGACCCGTT TCACGCCCCG ATCAAGCACT TGTTCCACAT GTTTCTATAC

1201 TCTGCGTCTG CGGAGACGTC ATTTTGCATG TCTGAAGTGG GTTTGGGGTT TGTATGGCTG

1261 TCGAGTAAAG AGCGCATGGA GATTTTTGTA AAATTCTAGA AAAAATACTT GTAGTTTCCT

1321 CGCTCATCAT AGTTATCATC CTGGACTCTC ACGTTCCACC CCCCCACGCT CACTACTAGC

1381 ACTTGCTGTA GTCCCCTCTC TCGTCTATCT ACCGCCCTAA TGCCGACTGC ACTATGCGAT

1441 GTCCAGATAA GGTAGCAATC CACACTTCTA GGTTTGTGAG GTGGTGTTGA GGTACCGACT

1501 TGATTGAGAC GACTGAAAGG ACCTCCAGCT ACATCTAAAA TCCCAGAGGA CGTGCGAAGA

1561 AGGTCTTTAA GCCCCTTTGT CTTCTAGCGA CCAAACCCAA CCCGTCAAGG CGACATACAT

1621 CACGTCGACC TCGTCCCATT GCTGAACCTA AACCCACGCA CTCCTTGTAT AACAGGACGT

1681 GGTCAACTCA ACTCTCTCTT GTACACATAC ACCACAACAC ACACAAAATC AATATAACTT

1741 CGTATAATGT ATGCTATACG AAGTTATCAC GGGCAAAAGT GCGTATATAT ACAAGAGCGT

1801 TTGCCAGCCA CAGATTTTCA CTCCACACAC CACATCACAC ATACAACCAC ACACATCCAC

1861 AATGGAACCC GAAACTAAGA TGAAAAAGCC TGAACTCACC GCGACGTCTG TCGAGAAGTT

1921 TCTGATCGAA AAGTTCGACA GCGTCTCCGA CCTGATGCAG CTCTCGGAGG GCGAAGAATC

1981 TCGTGCTTTC AGCTTCGATG TAGGAGGGCG TGGATATGTC CTGCGGGTAA ATAGCTGCGC

2041 CGATGGTTTC TACAAAGATC GTTATGTTTA TCGGCACTTT GCATCGGCCG CGCTCCCGAT

2101 TCCGGAAGTG CTTGACATTG GGGAGTTCAG CGAGAGCCTG ACCTATTGCA TCTCCCGCCG

2161 TGCACAGGGT GTCACGTTGC AAGACCTGCC TGAAACCGAA CTGCCCGCTG TTCTACAGCC

2221 GGTCGCGGAG GCAATGGATG CGATCGCTGC GGCCGATCTT AGCCAGACGA GCGGGTTCGG

2281 CCCATTCGGA CCGCAAGGAA TCGGTCAATA CACTACATGG CGTGATTTCA TTTGCGCGAT

2341 TGCTGATCCC CATGTGTATC ACTGGCAAAC TGTGATGGAC GACACCGTCA GTGCGTCCGT

2401 CGCGCAGGCT CTCGATGAGC TGATGCTTTG GGCCGAGGAC TGCCCCGAAG TCCGGCACCT

2461 CGTGCACGCG GATTTCGGCT CCAACAATGT CCTGACGGAC AATGGCCGCA TAACAGCGGT

2521 CATTGACTGG AGCGAGGCGA TGTTCGGGGA TTCCCAATAC GAGGTCGCCA ACATCTTCTT

2581 CTGGAGGCCG TGGTTGGCTT GTATGGAGCA GCAGACGCGC TACTTCGAGC GGAGGCATCC

2641 GGAGCTTGCA GGATCGCCGC GACTCCGGGC GTATATGCTC CGCATTGGTC TTGACCAACT

2701 CTATCAGAGC TTGGTTGACG GCAATTTCGA TGATGCAGCT TGGGCGCAGG GTCGATGCGA

2761 CGCAATCGTC CGATCCGGAG CCGGGACTGT CGGGCGTACA CAAATCGCCC GCAGAAGCGC

2821 GGCCGTCTGG ACCGATGGCT GTGTAGAAGT ACTCGCCGAT AGTGGAAACC GACGCCCCAG

2881 CACTCGTCCG AGGGCAAAGG AATAGGACTC TATAAAAAGG GCCCTGCCCT GCTAATGAAA

2941 TGATGATTTA TAATTTACCG GTGTAGCAAC CTTGACTAGA AGAAGCAGAT TGGGTGTGTT

3001 TGTAGTGGAG GACAGTGGTA CGTTTTGGAA ACAGTCTTCT TGAAAGTGTC TTGTCTACAG

3061 TATATTCACT CATAACCTCA ATAGCCAAGG GTGTAGTCGG TTTATTAAAG ATAACTTCGT

3121 ATAATGTATG CTATACGAAG TTATTTGTAG ACTCGTTGAA GCTGACGCGA TCCGACGCGA

3181 TTGTCTCAGA GGAATGGGTA AAAGACGAAT AAATTAAATC TATTGTATTT TACTGAACGC

3241 GACATGTACC GTATATGCTA AGTAGTAGCT GCTGTGTAAT GGAACCGTAC GCTAAAGTAT

3301 CGATGCGCCA AGCTGGTACA CGTGCACCGG TTGCGATCTG AGTGTGTGTT GTTTCCGTGG

3361 TAACGGGTGT GAAAAGGGAG CGTGGTTGGA AATGGGAATG GGGTTTGATA ATTTGTTTGT

3421 GACTGTTGTG TGGATTTGTA TCGAGTGTTT ATGATCGATG ACGTTGGTAA GGGGTATCGA

3481 GAGGAGCCAG AATTGGACGT GGCAAACTAT TTGACAAACG GACGCATGGG AACGGAACAG

3541 AGTGTTGAGT GTCGAGTACA ATAATTGAGA TGTAGAGCTG AAAGTGTTTC AGCTTATCAG

3601 CATCTGTTGT CGTGGTAGTT TCCTGATTAC AATGTATGTA CGGTAGGTGA TCCCCAGTCG

3661 TGCTACAGCG TCACGATCGA TGGGGCGCAA CACCACACAC TAGATCCTAA AGCTCGTGAG

3721 CATGGATGTA GTTTCTGGAG TTGGAATTTC GCAATCCCTT TTGCCCAAAC AGATCTCAAC

3781 TACAGTCGCA GCTAGTGAGT GTGTGCTATT GGCCTGCTGA GTGGCGTAAG GAGACGTCAT

3841 GAAAAGTCCT CATATTGAAG CAGTTTAGGG GTGATCAGGG CAGTCGAGAA GATAAATGTG

3901 ACAAGCTTGG ATGTATCAAC TTCATGATTA ACTGTTGGGG ACAGCCACTC GAATGTGGAA

3961 GTTGCTAATG GACTCACTCG ACTCAGGCAG ATGACATTAT GATAGAAAGT GGGGTAGTTT

4021 CAGTTGGATG CACTACCAAG AGAGCATTAT GTTCTATAAG TGGTGCTTGC GAAACATTCC

4081 GTAAACTCCA CGGAAGTTCT CAGACTTTCA ATGAGTCTAT ATCTCAACTT CTCCGGTCCC

4141 GATATCTTGT TTTTGTATTG AGGTACAGGT ATCGCACAAA GGCGGTTCCT CGGCAATACG

4201 GGGAACGTCA TTGACGCAAC CATGGGTCCC ATGCAATGCA TTAGCAGCAA TAACGCGCCG

4261 GTGGGTGTCG GTTTATGAGC ACAGGGCGGT TCACGTTTAC TGCATGATGA TCTATGGGGT

4321 GACGGTGAAT CTGTATCTGC TCTGAACGAA CGTCGGTGAT AGTCTCAGCC GCCCTGTACT

4381 ACAACCAGTG CTGTGAACTT CCAATTATCT ATAGAAAATG CAGATTGTTC ATTTCGCATA

4441 AGAACGAATA CTTGTACTTG TCTGACTCAT TATGTAAAAA ACAAAGGATC CTTCCTTTCT

4501 GGGATGTCCC CAAGGGAGGA GGGGGGGTGG CTGGTGACCC TGGTGAGGGG CTGGCATTGC

4561 CACAGGGGAC CTGGATCATC TCTGGAACGT TTATTCGACT TTCAGAGCAG CTCCAAATCG

4621 TAGCACAGGA CCTTTCTCCT TCTATTTTAT TCGGGATTTT GTCCCGTGCC CCTGCATAGT

4681 TGAATAGACG GCACTACTTT CAGCGCGGCC GCAGAGACGG AGTCACTGCC AACCGAGACG

4741 GTCATAGCTG TTTCCTGTGT GCCGCTTCCT CGCTCACTGA CTCGCTGCGC TCGGTCGTTC

4801 GGCTGCGGCG AGCGGTATCA GCTCACTCAA AGGCGGTAAT ACGGTTACCC ACAGAATCAG

4861 GGGATAACGC AGGAAAGAAC ATGTGAGCAA AAGGCCAGCA AAAGGCCAGG AACCGTAAAA

4921 AGGCCGCGTT GCTGGCGTTT TTCCATAGGC TCCGCCCCCC TGACGAGCAT CACAAAAATC

4981 GACGCTCAAG TCAGAGGTGG CGAAACCCGA CAGGACTATA AAGATACCAG GCGTTTCCCC

5041 CTGGAAGCTC CCTCGTGCGC TCTCCTGTTC CGACCCTGCC GCTTACCGGA TACCTGTCCG

5101 CCTTTCTCCC TTCGGGAAGC GTGGCGCTTT CTCAATGCTC ACGCTGTAGG TATCTCAGTT

5161 CGGTGTAGGT CGTTCGCTCC AAGCTGGGCT GTGTGCACGA ACCCCCCGTT CAGCCCGACC

5221 GCTGCGCCTT ATCCGGTAAC TATCGTCTTG AGTCCAACCC GGTAAGACAC GACTTATCGC

5281 CACTGGCAGC AGCCACTGGT AACAGGATTA GCAGAGCGAG GTATGTAGGC GGTGCTACAG

5341 AGTTCTTGAA GTGGTGGCCT AACTACGGCT ACACTAGAAG GACAGTATTT GGTATCTGCG

5401 CTCTGCTGAA GCCAGTTACC TTCGGAAAAA GAGTTGGTAG CTCTTGATCC GGCAAACAAA

5461 CCACCGCTGG TAGCGGTGGT TTTTTTGTTT GCAAGCAGCA GATTACGCGC AGAAAAAAAG

5521 GATCTCAAGA AGATCCTTTG ATCTTTTCTA CGGGGTCTGA CGCTCAGTGG AACGAAAACT

5581 CACGTTAAGG GATTTTGGTC ATGAGATTAT CAAAAAGGAT CTTCACCTAG ATCCTTTTAA

5641 ATTAAAAATG AAGTTTTAAA TCAATCTAAA GTATATATGA GTAAACTTGG TCTGACAGTT

5701 ACCAATGCTT AATCAGTGAG GCACCTATCT CAGCGATCTG TCTATTTCGT TCATCCATAG

5761 TTGCCTGACT CCCCGTCGTG TAGATAACTA CGATACGGGA GGGCTTACCA TCTGGCCCCA

5821 GTGCTGCAAT AATACCGCGG GACCCACGCT CACCGGCTCC AGATTTATCA GCAATAAACC

5881 AGCCAGCCGG AAGGGCCGAG CGCAGAAGTG GTCCTGCAAC TTTATCCGCC TCCATCCAGT

5941 CTATTAATTG TTGCCGGGAA GCTAGAGTAA GTAGTTCGCC AGTTAATAGT TTGCGCAACG

6001 TTGTTGCCAT CGCTACAGGC ATCGTGGTGT CACGCTCGTC GTTTGGTATG GCTTCATTCA

6061 GCTCCGGTTC CCAACGATCA AGGCGAGTTA CATGATCCCC CATGTTGTGC AAAAAAGCGG

6121 TTAGCTCCTT CGGTCCTCCG ATCGTTGTCA GAAGTAAGTT GGCCGCCGTG TTATCACTCA

6181 TGGTTATGGC AGCACTACAT AATTCTCTTA CTGTCATGCC ATCCGTAAGA TGCTTTTCTG

6241 TGACTGGTGA GTACTCAACC AAGTCATTCT GAGAATAGTG TATGCGGCGA CCGAGTTGCT

6301 CTTGCCCGGC GTCAATACGG GATAATACCG CGCCACATAG CAGAACTTTA AAAGTGCTCA

6361 TCATTGGAAA ACGTTCTTCG GGGCGAAAAC TCTCAAGGAT CTTACCGCTG TTGAGATCCA

6421 GTTCGATGTA ACCCACTCGT GCACCCAACT GATCTTCAGC ATCTTTTACT TTCACCAGCG

6481 TTTCTGGGTG AGCAAAAACA GGAAGGCAAA ATGCCGCAAA AAAGGGAATA AGGGCGACAC

6541 GGAAATGTTG AATACTCATA CTCTTCCTTT TTCAATATTA TTGAAGCATT TATCAGGGTT

6601 ATTGTCTCAT GAGCGGATAC ATATTTGAAT GTATTTAG


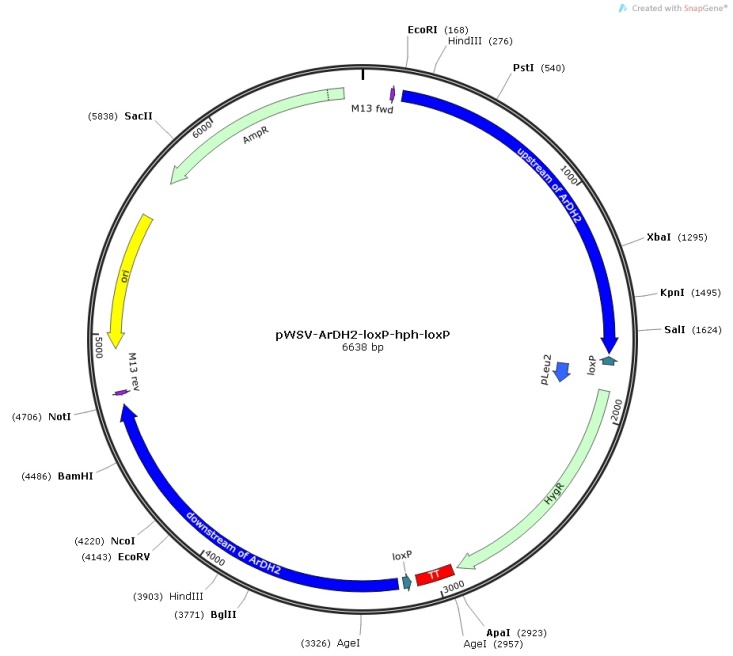


**Sequence 5: The *MDH1* gene knockout vector**

1-167: pUC19 derived vector sequence

168-1617: upstream sequence of MDH1 gene

1618-1652: loxP sequence

1653-1765: minimal promoter of Leu2 gene

1769-2795: hph gene

2796-3001: terminator sequence

3002-3036: loxP sequence

3037-4537: downstream sequence of MDH1 gene

4538-6468: pUC19 derived vector sequence

**The *MDH1* gene knockout cassette fragment from 168 bp to 4537 bp was synthesized in vitro.**

1 AAAAATAAAC AAATAGGGGT TCCGCGCACA TTTCCCCGAA AAGTGCCACC TGACGTCTAA

61 GAAACCATTA TTATCATGAC ATTAACCTAT AAAAATAGGC GTATCACGAG GCCCTTTCGT

121 TGTAAAACGA CGGCCAGTCG TCTCGATCCG CAGTGTCTTG CGTCTCTGAA TTCGGAGGAG

181 CGCCAGGCGG AGATTGATAT CAGTCAATAT GTGAATCTTA CTGAACAGCG AGTTCTGCTT

241 TATGTTTCTT TCCGAAATGT CTTTCTGGTC TCAAAGGTGT CCTCTGACGA CGTCAAGACG

301 TTTATGGTGA GCAACCATGA GCTGAAACTC ATCAACACCC AGAAGAGAAG ATACATTCCA

361 ACAGGCATAG TTTGTTACGA TGGAATATTG TCTGAAATTC ATTTTTGTGC ACTTCTTGGG

421 GGGATTGATG TTGTGGAAAT GGATTCGGCC GAAGCTGACC CCAGCACTCC ATTCAATCGA

481 ATGAAGACTG TAATTCAGGA TTCGGTTCAC AGCCGCTACG CTGTTTTGTA TAAACAAACT

541 GGCCTAATCT GTGGCATTCT AGATCTTGAA AAGAAGCAAA AAATGGACTT CTTCCTACGA

601 TCGAGCCTCG AAGAAGATTG GGCACATGGA CATCTGGTTA TGGCTGGTGT GTCCAAGGGC

661 GAGGTCGGAC TATGGGCCTT TTCCCATGCG TATATCAAAA GAGAGCTGGA GAAGGTCTCT

721 TTAGAGAAGG TATAAATCTG GAACTGTTTA CTAAGAACAA CACGTGAGTG TACCAAGCCT

781 GGCTGCTGGA CAGCGTGTGG CATCCTATAG GAATCAGCTG GTATGTATCT CTCCTACCCG

841 GCCTCCACCC TCGAGACTTG CTCATGGCTG ACTTACGCAT GCTGCAACTT ACTCGCAGGG

901 GCTGCTATTG ATAGACCAGA GAATCAACTA TTTTGGAAAG ATAACTACCT GTGTGGTAAT

961 GAGTAATGCT GTACTAATTC AGTAATACCG CATGCAACAA GCAACGCCCC ACATGTTGAT

1021 ACTTGTGTGC AGGTGTCAAG ATTCGATCCA TGAGATTAAC TTTAGAAACA TTATCCTAAG

1081 CACACATTTT AAGAGCAGCA AAAAAATATT TTACGGTATA GACATGTTGA GCTCAGGTTA

1141 TTTTTATCTA CGGATCAATT GCAACTACTG AAACCTATGC TGCACAATTC ATCGCGATCT

1201 CAAACTCCAT CTTTTGTCTG TCTATCTCAT CTTGGTTATG CAGCCACTTG CGCTTCTGAA

1261 AAGTTTAATA CCTTGACTAA AATATGGGGG CGATGGCTGT AAACTGTCAA TAGTGAGCCT

1321 ATGGATAGAA TAGTAATACC TGTATTCCCA AAAAAAACCT AACAAACACC TCTAGTTTAT

1381 CAATTTTGGT AATCCTTCAT ACGTACCGTA GCACCGAAGC ACGCTGTACC AAAACTGCCT

1441 TACACCACAG GCAAACTCAA TTCCGAGCTT GTCTCACACG TCTTGCTCCC TATCCTTTTA

1501 CTAACGTTTG TGTCAGCAAT CATTTCCAGT GGGTGGCAAT CGAAAACTCA CAAAAAACAG

1561 GTATATATAT CTCTTGATGA ACCCCATGTA CCCCTATCAA GCACTACAGC TATCTCCACA

1621 ACAATAACTT CGTATAATGT ATGCTATACG AAGTTATCAC GGGCAAAAGT GCGTATATAT

1681 ACAAGAGCGT TTGCCAGCCA CAGATTTTCA CTCCACACAC CACATCACAC ATACAACCAC

1741 ACACATCCAC AATGGAACCC GAAACTAAGA TGAAAAAGCC TGAACTCACC GCGACGTCTG

1801 TCGAGAAGTT TCTGATCGAA AAGTTCGACA GCGTCTCCGA CCTGATGCAG CTCTCGGAGG

1861 GCGAAGAATC TCGTGCTTTC AGCTTCGATG TAGGAGGGCG TGGATATGTC CTGCGGGTAA

1921 ATAGCTGCGC CGATGGTTTC TACAAAGATC GTTATGTTTA TCGGCACTTT GCATCGGCCG

1981 CGCTCCCGAT TCCGGAAGTG CTTGACATTG GGGAGTTCAG CGAGAGCCTG ACCTATTGCA

2041 TCTCCCGCCG TGCACAGGGT GTCACGTTGC AAGACCTGCC TGAAACCGAA CTGCCCGCTG

2101 TTCTACAGCC GGTCGCGGAG GCAATGGATG CGATCGCTGC GGCCGATCTT AGCCAGACGA

2161 GCGGGTTCGG CCCATTCGGA CCGCAAGGAA TCGGTCAATA CACTACATGG CGTGATTTCA

2221 TTTGCGCGAT TGCTGATCCC CATGTGTATC ACTGGCAAAC TGTGATGGAC GACACCGTCA

2281 GTGCGTCCGT CGCGCAGGCT CTCGATGAGC TGATGCTTTG GGCCGAGGAC TGCCCCGAAG

2341 TCCGGCACCT CGTGCACGCG GATTTCGGCT CCAACAATGT CCTGACGGAC AATGGCCGCA

2401 TAACAGCGGT CATTGACTGG AGCGAGGCGA TGTTCGGGGA TTCCCAATAC GAGGTCGCCA

2461 ACATCTTCTT CTGGAGGCCG TGGTTGGCTT GTATGGAGCA GCAGACGCGC TACTTCGAGC

2521 GGAGGCATCC GGAGCTTGCA GGATCGCCGC GACTCCGGGC GTATATGCTC CGCATTGGTC

2581 TTGACCAACT CTATCAGAGC TTGGTTGACG GCAATTTCGA TGATGCAGCT TGGGCGCAGG

2641 GTCGATGCGA CGCAATCGTC CGATCCGGAG CCGGGACTGT CGGGCGTACA CAAATCGCCC

2701 GCAGAAGCGC GGCCGTCTGG ACCGATGGCT GTGTAGAAGT ACTCGCCGAT AGTGGAAACC

2761 GACGCCCCAG CACTCGTCCG AGGGCAAAGG AATAGGACTC TATAAAAAGG GCCCTGCCCT

2821 GCTAATGAAA TGATGATTTA TAATTTACCG GTGTAGCAAC CTTGACTAGA AGAAGCAGAT

2881 TGGGTGTGTT TGTAGTGGAG GACAGTGGTA CGTTTTGGAA ACAGTCTTCT TGAAAGTGTC

2941 TTGTCTACAG TATATTCACT CATAACCTCA ATAGCCAAGG GTGTAGTCGG TTTATTAAAG

3001 ATAACTTCGT ATAATGTATG CTATACGAAG TTATGTAACA GAGTCCTATT GAAGTAGGGT

3061 TTCCTACAGT CATGTGTAAC CGGCAATATA TTGAGGATCA CTATATAATC TATAAATAAG

3121 GTGTTAGTTT CTCGCTTCCT TGTTCTTCTA GAAGTCCAAC TCAAACTTAT CAGCATACTC

3181 TCGGAGCTGC GCAAAGGTGA CGTTAGTGCC TCCGCAGACA ATCACAATAA CTGCGTCTTC

3241 GGGTCCGAGA CCAGGCACAA TATCTTTTAG TGTACCGTTG TAGATTGGAG CCAGCGCAGT

3301 TCCACAAGCA GCCTCAACAA GCACCTTGTG GTCGGCTACG AACTCGAGAC AGGATGCCGC

3361 TGCCTCCCGG TCAGTCACCT TGACAGCTGT AGTGGGATGT TTCTTGGCCC ATTCGATTGT

3421 CTCTCGAGTC ACGTTCTGAG TAGCCAGCGA AGAAGCAATG GTGTGAGGTC GATCAAGGCA

3481 GACCTGGGAG CCTGTTGCCA GAGAGTCAAA CAGGGTAGAG CAACCTTCAG TCTCGACTGC

3541 CACCAAAGGA ATTTTCTGCC ACTGCTTCTT TCCCGAGTTC TCAAAGCCGG TGCAGATTCC

3601 ATTAAACAGC CCACCGCCGC CAACAGAACA AACCACAGCA GCGGGGTTGT AGTCAGGAAA

3661 CAATGTCTCA GTCTGGACCA CAATTTCATC AGCCACGGTA GCATTTCCAG TCCACACGAG

3721 GGGATCGTTG TAGGGATGGC AGTATACGGG CTTTTCGAGA GAAAGGTCGC ATGCCGGAAT

3781 AAGAATCTCC TTGAGGTACT TGTCTGCGTC AGCAATGTAT TGGCCATAAG GAATAACTCG

3841 AGCGCCAGTC TTTTCAATTC GCTGGATCAT TCCGGGGGGG GTCGAGAGAG GAACACAGAC

3901 GGTGCAGAGC TGGTTGTATT TTTTGGCGGC GTATGCAGTG GCACAGCCAG CGTTTCCTCC

3961 AGAAGAAGAA AAGAAGTGCA GCTTGGTTGT GTTTGTGTCG GTGGGGGCAT CCTCGGCGTT

4021 TCCAAAGTGA GTCTGGACGT GTTTGTAGAC CAGGTGTCCA AGACCTCGAG ACTTGAAGGA

4081 GCCAGAGGGC TGTGTAGTTT CGTATTTGAG AAGCACCTTA CATCCTGCTA GCTGTGAGAG

4141 GTACAGAGAC TCGATCAAAG GCGTCTGGAT GTATGGAAGG GTGGTTTTAG GGATGGGGGA

4201 GGCTGCCGGA GAGGGTGGAG ATGGGGGAGT AGAGGTTGTC ATTGCACACG GAAGAACTCG

4261 TGGATTCAGG AGTTTTTAGG TGGAGTGTTT TGTTTGTGCT GTACAGTATC TGACAAGCCA

4321 GGAGTCGTTT GCTTGTATAT AAAGGGTCTT CAGTTAGGTC AGCTGAGATA ACAGAAGATG

4381 AGATAAGGAT ATGAGAACTG ATGATGACGT ACTTCTATGT TGGATGCACT GTATTGTAAT

4441 GCAACAGCGT TGAGGCAGAA GAAAAGTCAG AGTCGGTGTG TTATTAATAT TCCTTTTGGG

4501 GTCGAGACGT TATCTTATCG TAAGAAATTT AGGCGCGGCC GCAGAGACGG AGTCACTGCC

4561 AACCGAGACG GTCATAGCTG TTTCCTGTGT GCCGCTTCCT CGCTCACTGA CTCGCTGCGC

4621 TCGGTCGTTC GGCTGCGGCG AGCGGTATCA GCTCACTCAA AGGCGGTAAT ACGGTTACCC

4681 ACAGAATCAG GGGATAACGC AGGAAAGAAC ATGTGAGCAA AAGGCCAGCA AAAGGCCAGG

4741 AACCGTAAAA AGGCCGCGTT GCTGGCGTTT TTCCATAGGC TCCGCCCCCC TGACGAGCAT

4801 CACAAAAATC GACGCTCAAG TCAGAGGTGG CGAAACCCGA CAGGACTATA AAGATACCAG

4861 GCGTTTCCCC CTGGAAGCTC CCTCGTGCGC TCTCCTGTTC CGACCCTGCC GCTTACCGGA

4921 TACCTGTCCG CCTTTCTCCC TTCGGGAAGC GTGGCGCTTT CTCAATGCTC ACGCTGTAGG

4981 TATCTCAGTT CGGTGTAGGT CGTTCGCTCC AAGCTGGGCT GTGTGCACGA ACCCCCCGTT

5041 CAGCCCGACC GCTGCGCCTT ATCCGGTAAC TATCGTCTTG AGTCCAACCC GGTAAGACAC

5101 GACTTATCGC CACTGGCAGC AGCCACTGGT AACAGGATTA GCAGAGCGAG GTATGTAGGC

5161 GGTGCTACAG AGTTCTTGAA GTGGTGGCCT AACTACGGCT ACACTAGAAG GACAGTATTT

5221 GGTATCTGCG CTCTGCTGAA GCCAGTTACC TTCGGAAAAA GAGTTGGTAG CTCTTGATCC

5281 GGCAAACAAA CCACCGCTGG TAGCGGTGGT TTTTTTGTTT GCAAGCAGCA GATTACGCGC

5341 AGAAAAAAAG GATCTCAAGA AGATCCTTTG ATCTTTTCTA CGGGGTCTGA CGCTCAGTGG

5401 AACGAAAACT CACGTTAAGG GATTTTGGTC ATGAGATTAT CAAAAAGGAT CTTCACCTAG

5461 ATCCTTTTAA ATTAAAAATG AAGTTTTAAA TCAATCTAAA GTATATATGA GTAAACTTGG

5521 TCTGACAGTT ACCAATGCTT AATCAGTGAG GCACCTATCT CAGCGATCTG TCTATTTCGT

5581 TCATCCATAG TTGCCTGACT CCCCGTCGTG TAGATAACTA CGATACGGGA GGGCTTACCA

5641 TCTGGCCCCA GTGCTGCAAT AATACCGCGG GACCCACGCT CACCGGCTCC AGATTTATCA

5701 GCAATAAACC AGCCAGCCGG AAGGGCCGAG CGCAGAAGTG GTCCTGCAAC TTTATCCGCC

5761 TCCATCCAGT CTATTAATTG TTGCCGGGAA GCTAGAGTAA GTAGTTCGCC AGTTAATAGT

5821 TTGCGCAACG TTGTTGCCAT CGCTACAGGC ATCGTGGTGT CACGCTCGTC GTTTGGTATG

5881 GCTTCATTCA GCTCCGGTTC CCAACGATCA AGGCGAGTTA CATGATCCCC CATGTTGTGC

5941 AAAAAAGCGG TTAGCTCCTT CGGTCCTCCG ATCGTTGTCA GAAGTAAGTT GGCCGCCGTG

6001 TTATCACTCA TGGTTATGGC AGCACTACAT AATTCTCTTA CTGTCATGCC ATCCGTAAGA

6061 TGCTTTTCTG TGACTGGTGA GTACTCAACC AAGTCATTCT GAGAATAGTG TATGCGGCGA

6121 CCGAGTTGCT CTTGCCCGGC GTCAATACGG GATAATACCG CGCCACATAG CAGAACTTTA

6181 AAAGTGCTCA TCATTGGAAA ACGTTCTTCG GGGCGAAAAC TCTCAAGGAT CTTACCGCTG

6241 TTGAGATCCA GTTCGATGTA ACCCACTCGT GCACCCAACT GATCTTCAGC ATCTTTTACT

6301 TTCACCAGCG TTTCTGGGTG AGCAAAAACA GGAAGGCAAA ATGCCGCAAA AAAGGGAATA

6361 AGGGCGACAC GGAAATGTTG AATACTCATA CTCTTCCTTT TTCAATATTA TTGAAGCATT

6421 TATCAGGGTT ATTGTCTCAT GAGCGGATAC ATATTTGAAT GTATTTAG


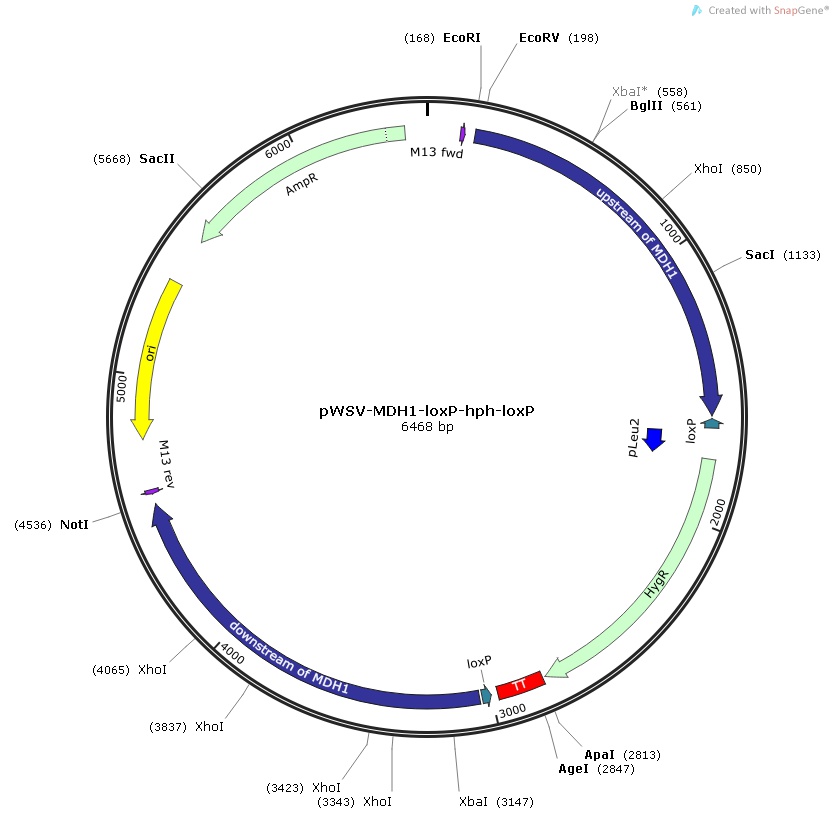


**Sequence 6: The *MDH2* gene knockout vector**

1-167: pUC19 derived vector sequence

168-1673: upstream sequence of MDH2 gene

1674-1708: loxP sequence

1709-1820: minimal promoter of Leu2 gene

1821-2846: hph gene

2847-3051: terminator sequence

3052-3085: loxP sequence

3086-4585: downstream sequence of MDH2 gene

4586-6518: pUC19 derived vector sequence

**The *MDH2* gene knockout cassette fragment from 168 bp to 4585 bp was synthesized in vitro.**

1 AAAAATAAAC AAATAGGGGT TCCGCGCACA TTTCCCCGAA AAGTGCCACC TGACGTCTAA

61 GAAACCATTA TTATCATGAC ATTAACCTAT AAAAATAGGC GTATCACGAG GCCCTTTCGT

121 TGTAAAACGA CGGCCAGTCG TCTCGATCCG CAGTGTCTTG CGTCTCTGAA TTCGTACTGA

181 GGACGAGGGG ATTCTGCCAA TGGTGTTAGT TTTCCAATTG CTCTTGTATT GGCCAAGTTG

241 AAGACGTTCT TGTTGCCACT TCCACCCAAA TGATCCCCAA ACGAACTACT GCATGGCGTC

301 CGGGTAACCG CCGAGGGAGG GTCTTTGTCT GTTAATTAGT ACTTTTGAGA AATACACCGG

361 AGTATTATTA TGGTGGATTT CCGGGCTCCT CTGTGACCCG AGTGTTGCTG GACGTTCGAT

421 GTTCGATGCT CGGCACATGC CGGTTCGAAC AGGAATTATA GCGTTCATCT GGAGTTGGAC

481 GCAAGCAAAA AAGCAAATGA GGGAGTTATG GGAGGGTTCC GAGAAGTGAA AAATCGGTCA

541 ATGGGTTAGT TTGAAGTCTC GTTTTGTTCG TGTTGGCGAG ACAAGAAGAA TGGTATAATT

601 TTCGCACCAA AAAGAGACCG TTTATCGTGG ATTATGGGGG TGTGATGTGG GGGGAGGGGG

661 GAGATGCCCC ATCTCTGGCA ACCCTATTTG ACGATAGTTG CTGGAGGCTT GACAGGACTT

721 GGTGACGAGG GGTGTTTGGG CGCTGGAAGC GTAATTTTCG TCTTGAATGG GCCGTCGAGA

781 CTTGGGGTTC GACCCCGACT AAATGGCGCA CCGCTAGATT CTCTTTTGGC GACTTTCTCG

841 GGATTCTAGT CACCCCCGCA ATGTTCCAGC TTACGGTTTG AGACAGTACA CGACTGGCTA

901 GGCGAGTTGT TGAAGTCGTA GCGTAGAGTG GGAGGCATGA CGTCACGGGA CAGCTGCGTG

961 CACCACGCGA GCAGGTCAAT TGACCTCATT TGAGTGGTGT GGCTTGGCGT TCTAGCGGTG

1021 GCGGCGTTGT CGAGCTCCCT CTACTTGTAG TGAGATTATG TCGACGAGCG GGGGGGACTT

1081 CCATTGTGCT TGCCACTGCT AGTGCAGTAC AACTGAAAGC TAAACCGCAA TCAATCCCAA

1141 ACTGCATGTC CGCCTTAACT CTGATATGTT ATCAAGAGAG TGGTGTGGTG AGGTGAGGTG

1201 AGGTGACGTG GACAAGTTGA TGGGGAGTTG GGGCATTGAC AAAAGGGAAA TTGCAGGGGG

1261 ATTCCGCCGG CTATATATAT CTTATGTCTG CTCAATTCCC AGACGGCTCC ACACAAAACC

1321 AAGATACCAC ACCATCATGG TCACACCGGG TACATAACTC CCATCCATCT CATCCCACTT

1381 GCATGGCGAC CGGAGAGAGA AAGCCCGGGG AGAGCACGTC GGCGCGGTCC CCAGGGCGAC

1441 AACCAAAACA AAATCACCGA GTGACTCCGA AAGCCGCGTT CCAACACCCC CCCAAAATCC

1501 CCCCCTCAAA CACGTCAGCC ACCTGTCCCC CGAAAATTAA CTTCACTGAC ATGGCGCAGC

1561 TATTAAGGCT AAAGTGAATG CATGGCTCAT CTTTGTTTGC TGGTTGCTAC TGTGACTGAG

1621 GTAAAAACCC TCGCTCCCAA GTCTATATAT ACCTGGGTGT GCTCCCTCGA ACAATAACTT

1681 CGTATAATGT ATGCTATACG AAGTTATCAC GGGCAAAAGT GCGTATATAT ACAAGAGCGT

1741 TTGCCAGCCA CAGATTTTCA CTCCACACAC CACATCACAC ATACAACCAC ACACATCCAC

1801 AATGGAACCC GAAACTAAGA TGAAAAAGCC TGAACTCACC GCGACGTCTG TCGAGAAGTT

1861 TCTGATCGAA AAGTTCGACA GCGTCTCCGA CCTGATGCAG CTCTCGGAGG GCGAAGAATC

1921 TCGTGCTTTC AGCTTCGATG TAGGAGGGCG TGGATATGTC CTGCGGGTAA ATAGCTGCGC

1981 CGATGGTTTC TACAAAGATC GTTATGTTTA TCGGCACTTT GCATCGGCCG CGCTCCCGAT

2041 TCCGGAAGTG CTTGACATTG GGGAGTTCAG CGAGAGCCTG ACCTATTGCA TCTCCCGCCG

2101 TGCACAGGGT GTCACGTTGC AAGACCTGCC TGAAACCGAA CTGCCCGCTG TTCTACAGCC

2161 GGTCGCGGAG GCAATGGATG CGATCGCTGC GGCCGATCTT AGCCAGACGA GCGGGTTCGG

2221 CCCATTCGGA CCGCAAGGAA TCGGTCAATA CACTACATGG CGTGATTTCA TTTGCGCGAT

2281 TGCTGATCCC CATGTGTATC ACTGGCAAAC TGTGATGGAC GACACCGTCA GTGCGTCCGT

2341 CGCGCAGGCT CTCGATGAGC TGATGCTTTG GGCCGAGGAC TGCCCCGAAG TCCGGCACCT

2401 CGTGCACGCG GATTTCGGCT CCAACAATGT CCTGACGGAC AATGGCCGCA TAACAGCGGT

2461 CATTGACTGG AGCGAGGCGA TGTTCGGGGA TTCCCAATAC GAGGTCGCCA ACATCTTCTT

2521 CTGGAGGCCG TGGTTGGCTT GTATGGAGCA GCAGACGCGC TACTTCGAGC GGAGGCATCC

2581 GGAGCTTGCA GGATCGCCGC GACTCCGGGC GTATATGCTC CGCATTGGTC TTGACCAACT

2641 CTATCAGAGC TTGGTTGACG GCAATTTCGA TGATGCAGCT TGGGCGCAGG GTCGATGCGA

2701 CGCAATCGTC CGATCCGGAG CCGGGACTGT CGGGCGTACA CAAATCGCCC GCAGAAGCGC

2761 GGCCGTCTGG ACCGATGGCT GTGTAGAAGT ACTCGCCGAT AGTGGAAACC GACGCCCCAG

2821 CACTCGTCCG AGGGCAAAGG AATAGGACTC TATAAAAAGG GCCCTGCCCT GCTAATGAAA

2881 TGATGATTTA TAATTTACCG GTGTAGCAAC CTTGACTAGA AGAAGCAGAT TGGGTGTGTT

2941 TGTAGTGGAG GACAGTGGTA CGTTTTGGAA ACAGTCTTCT TGAAAGTGTC TTGTCTACAG

3001 TATATTCACT CATAACCTCA ATAGCCAAGG GTGTAGTCGG TTTATTAAAG ATAACTTCGT

3061 ATAATGTATG CTATACGAAG TTATTAATGA TTGTTTATGA TTAGACATTG ATTGAGTGTA

3121 GTTGGACATT AGCAGTCAGA TAGGCAACGA AGATCATCCA AGTCTGAATA CATACCCATA

3181 CAAATCATAC AAGTAAATGA TGGAATTACT CATATAAGTA TGTACTTACT TGTACCGAAT

3241 TGCCAATGAA TGTCAATCAG AACGCAGTAT GTACAAGTAC TCGCACAATA TCATAAGGCA

3301 CTCGAATGTT CAAGAAGTCA TCATTTTGGT GATTCGGGGA AATACTTGAC ACCTTTGTTG

3361 ATGCAACTTG ACTCCATAAG TAGGAAACCC ATAGTATATC TTTTTGTCGC TTTATATTCA

3421 CCTGTTCCCT TCTTTCTATG GACTATAAGT TAATTTAGTT GACCTTGTCA AGTAATACCT

3481 CAACAAACTG TAAAGAAATA GGAGATTATT GCTTTTGGTT TTTGAGAAGA GATCTGGATA

3541 GCACCACACA AATAATGCGT CAAAATCAGT TCAAAATCCA ACCCACAGAA CAACCAACAC

3601 TCCCCGAACC GCTCATAACC TGTATAGGAT GCTGCTCCAC TCACACCTCT TGTCCAGCCC

3661 TAACCTGCGA TCTAGGTTGG GGAAATTTTG TATGCAGGAA AAATATATGC AAGATTTGGG

3721 ATTTTATTTG TGTTCATCAA CCACGTCGAT TTACACAGCT ATCATGGTCC GAGAACGATC

3781 TGGAACGGTG GCGTACACGC CCAAAAAGCA GCAGAAGCCT CTGGTGGACG ACGCCCACTT

3841 GTCCTCTGCT GAGGAGGACG ATTTCAAGAC CCCAGAGAGT GCAAAGAGCA AGAAGCCTGA

3901 AGCGTCACAA CAGGAGCCGG CGGTGTCAGA AACGCCAGTC AAGGGCAAGG CCAAGAAGAT

3961 CACCTTTGAC GAGGACGGAA TGAGTGCCGA GCCCATCGTT GAGAAGAAAA AGGTGGTTGT

4021 GGAGGAGTCT GAAGACGACA GTGACGATGC CCCCGAGGAG GAAACACTGG AGGATGGACA

4081 GGAAAAGACC CTGGCCAAGC AAAAGGAACA GCAGCGGCTG GCAGCATTAG AGAAGGCGGA

4141 AGAGAAGAAG AAGCGGCGGG AGGCCAACGA GGTGCTGGCT AAACAGGCCA AGGCCAAGAA

4201 GGACAAGCTG GAGGAGCTCA GAAGACAGGC TCGAGAGGAT GAGGAGGAGG ACGAGGAAGA

4261 AGAAGAGGAT GAAGACGACG ATGAGGATCA AGAACAGCTG CCTCTGGAGG TTCTAGAGGC

4321 ACTGGAGCGG TCCAAGAACA TGCCTGTGGA GGAGCCCAAG AAGCCCAAGA AGCGGGTGTT

4381 TGAGGAGGTT GAGGAGGAGA TCAAGAAGGG TCCTGTTTCC GTGCGGGTAC TCAAAAAGAA

4441 CAAGTCTAAG CTGCCCCCTA AGGCCGAGGG CGCTACTACA GTCGGACGAA TGGCTTTTTT

4501 GAACAGACCT TCGATCGACC GACGACAGGT CAAGAGATGA TGGTGAGGAT GATGCACTTC

4561 ACATAGATAA TGGATGGTAA TTATGCGGCC GCAGAGACGG AGTCACTGCC AACCGAGACG

4621 GTCATAGCTG TTTCCTGTGT GCCGCTTCCT CGCTCACTGA CTCGCTGCGC TCGGTCGTTC

4681 GGCTGCGGCG AGCGGTATCA GCTCACTCAA AGGCGGTAAT ACGGTTACCC ACAGAATCAG

4741 GGGATAACGC AGGAAAGAAC ATGTGAGCAA AAGGCCAGCA AAAGGCCAGG AACCGTAAAA

4801 AGGCCGCGTT GCTGGCGTTT TTCCATAGGC TCCGCCCCCC TGACGAGCAT CACAAAAATC

4861 GACGCTCAAG TCAGAGGTGG CGAAACCCGA CAGGACTATA AAGATACCAG GCGTTTCCCC

4921 CTGGAAGCTC CCTCGTGCGC TCTCCTGTTC CGACCCTGCC GCTTACCGGA TACCTGTCCG

4981 CCTTTCTCCC TTCGGGAAGC GTGGCGCTTT CTCAATGCTC ACGCTGTAGG TATCTCAGTT

5041 CGGTGTAGGT CGTTCGCTCC AAGCTGGGCT GTGTGCACGA ACCCCCCGTT CAGCCCGACC

5101 GCTGCGCCTT ATCCGGTAAC TATCGTCTTG AGTCCAACCC GGTAAGACAC GACTTATCGC

5161 CACTGGCAGC AGCCACTGGT AACAGGATTA GCAGAGCGAG GTATGTAGGC GGTGCTACAG

5221 AGTTCTTGAA GTGGTGGCCT AACTACGGCT ACACTAGAAG GACAGTATTT GGTATCTGCG

5281 CTCTGCTGAA GCCAGTTACC TTCGGAAAAA GAGTTGGTAG CTCTTGATCC GGCAAACAAA

5341 CCACCGCTGG TAGCGGTGGT TTTTTTGTTT GCAAGCAGCA GATTACGCGC AGAAAAAAAG

5401 GATCTCAAGA AGATCCTTTG ATCTTTTCTA CGGGGTCTGA CGCTCAGTGG AACGAAAACT

5461 CACGTTAAGG GATTTTGGTC ATGAGATTAT CAAAAAGGAT CTTCACCTAG ATCCTTTTAA

5521 ATTAAAAATG AAGTTTTAAA TCAATCTAAA GTATATATGA GTAAACTTGG TCTGACAGTT

5581 ACCAATGCTT AATCAGTGAG GCACCTATCT CAGCGATCTG TCTATTTCGT TCATCCATAG

5641 TTGCCTGACT CCCCGTCGTG TAGATAACTA CGATACGGGA GGGCTTACCA TCTGGCCCCA

5701 GTGCTGCAAT AATACCGCGG GACCCACGCT CACCGGCTCC AGATTTATCA GCAATAAACC

5761 AGCCAGCCGG AAGGGCCGAG CGCAGAAGTG GTCCTGCAAC TTTATCCGCC TCCATCCAGT

5821 CTATTAATTG TTGCCGGGAA GCTAGAGTAA GTAGTTCGCC AGTTAATAGT TTGCGCAACG

5881 TTGTTGCCAT CGCTACAGGC ATCGTGGTGT CACGCTCGTC GTTTGGTATG GCTTCATTCA

5941 GCTCCGGTTC CCAACGATCA AGGCGAGTTA CATGATCCCC CATGTTGTGC AAAAAAGCGG

6001 TTAGCTCCTT CGGTCCTCCG ATCGTTGTCA GAAGTAAGTT GGCCGCCGTG TTATCACTCA

6061 TGGTTATGGC AGCACTACAT AATTCTCTTA CTGTCATGCC ATCCGTAAGA TGCTTTTCTG

6121 TGACTGGTGA GTACTCAACC AAGTCATTCT GAGAATAGTG TATGCGGCGA CCGAGTTGCT

6181 CTTGCCCGGC GTCAATACGG GATAATACCG CGCCACATAG CAGAACTTTA AAAGTGCTCA

6241 TCATTGGAAA ACGTTCTTCG GGGCGAAAAC TCTCAAGGAT CTTACCGCTG TTGAGATCCA

6301 GTTCGATGTA ACCCACTCGT GCACCCAACT GATCTTCAGC ATCTTTTACT TTCACCAGCG

6361 TTTCTGGGTG AGCAAAAACA GGAAGGCAAA ATGCCGCAAA AAAGGGAATA AGGGCGACAC

6421 GGAAATGTTG AATACTCATA CTCTTCCTTT TTCAATATTA TTGAAGCATT TATCAGGGTT

6481 ATTGTCTCAT GAGCGGATAC ATATTTGAAT GTATTTAG


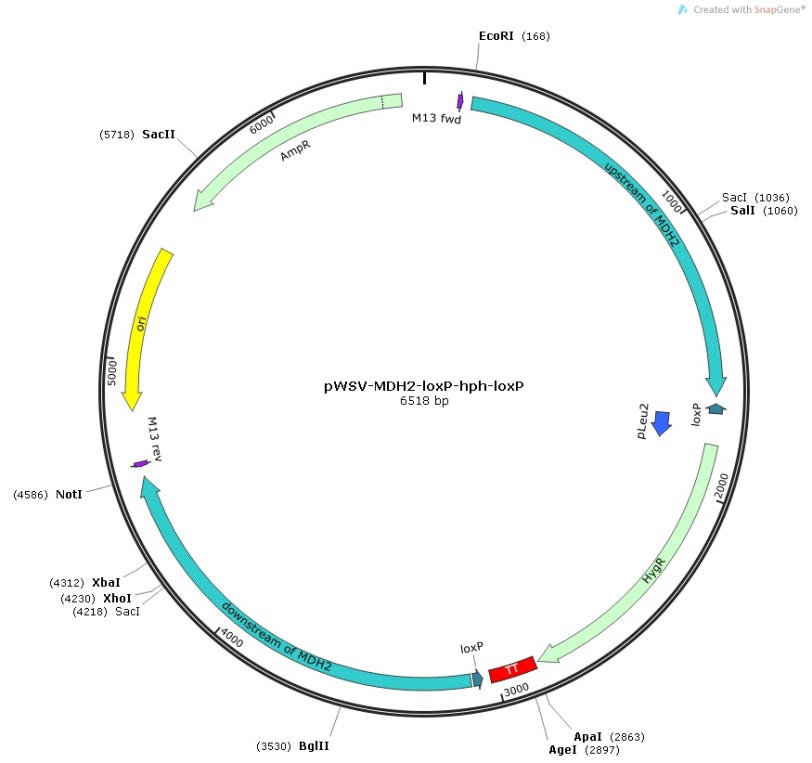


**Sequence 7: The *XDH1* gene knockout vector**

1-167: pUC19 derived vector sequence

168-1673: upstream sequence of XDH1 gene

1674-1708: loxP sequence

1709-1820: minimal promoter of Leu2 gene

1821-2846: hph gene

2847-3051: terminator sequence

3052-3085: loxP sequence

3086-4585: downstream sequence of XDH1 gene

4586-6518: pUC19 derived vector sequence

**The *XDH1* gene knockout cassette fragment from 168 bp to 4585 bp was synthesized in vitro.**

1 AAAAATAAAC AAATAGGGGT TCCGCGCACA TTTCCCCGAA AAGTGCCACC TGACGTCTAA

61 GAAACCATTA TTATCATGAC ATTAACCTAT AAAAATAGGC GTATCACGAG GCCCTTTCGT

121 TGTAAAACGA CGGCCAGTCG TCTCGATCCG CAGTGTCTTG CGTCTCTGAA TTCTGTAGTC

181 AGAACCGCAA AGGTTCTCAG GTCCAGCAAT TAGCGGGCGG GGGGCGTGTT GGAGGAGATG

241 TATTGGTTTC TTCAGTTTAT TTCTGTCCAT TCTGAACTGT TTCGTTGCTT TTGTGTCGTC

301 AACTTATCGT CTACAGTACC TTCCTAGTCT CCTATGTCGA TGATCAGTCA GCATCATAGT

361 TGCAGCACGA AATGTTCTCA TACGAGTCAG CGAAAGAATC GGTTCTCCTG CACTTATTCT

421 GCTCATGTGT GTACTGTAAC CTCCTTTATG CCAATCACAG ACGGGCAATA TATAACGCAG

481 GTATTGGACA ATTAAAAAAG TATGGGAAGG GTCTTTTGAA AGAAGTGGAG TGAGCGTGCC

541 TCAGGAATTG AGAGAAGATG GCCATCAACT TTCGGTCATT GCAAGTCATT GTTTTTTTCA

601 ACACAAGCAT TCAGAGTTGC CCAAGTAAGC ATAGCCAACC ATTGTCTTTA GTCAGCTTAT

661 TTTAGCCCAA GACCTCTCCC TTGTTCCAAC TCATGTTCCA ACTCATCTCC ACAGTCCATT

721 TAACCACCTA CTTCGCACCA CTGGGTATTG TACAGTACAA GTAATACCGA GTACAAAACA

781 GAGTCACTGA AAACACGCCA GTTCAATTGG CACGCGGAAC AGCACCAATA TCACTGTTTA

841 CTCAAACACT TCCCTGTAAC CGTGATGATT CCAACACTTG ACTCTACGGC CTTCTTCAGA

901 CAACCACTCA CCGGTAGGTA CAGCACGAGC AAACACTCTC TACTTGTACA ATAGTAGAAC

961 AACGCCCTTG AACACTCCAT TCGAGACCAA CAACCACGCC ATAGTCAACA CCAGACCCGC

1021 ACCTCTAATT ATAAGACCAG CCATTCCCAG CACTTTCCAA AACCACATGT CTCAAGGTCT

1081 GTTTTCTGAA TACGAGTTTG ACAGGTGACA TTGGACAGAC CTTTCAGCCA ATGGAGCATT

1141 GCGCCACCAG GAAGAAAACA AAACAATTGT TAACCACCAG AAAGTGCCAC ATCATGAACA

1201 TCTTGTCAGA CATGTTGACC CGCCGCATTC TACTATTGCC CCCAAGCTGC TCCTACAATA

1261 CTGGTACAAT ACTTGCTCTC AAGTCCCATG TGGCCATCAG ACCATACGAA GCTTATCAAC

1321 ACCTTAAACC GGTACCCAAT GCTCCCAACG AAACTCTGGA CAATGATCAT CCATGGAATA

1381 GAGAAGAAGG AGGAGAAGGA CGAGACGCAC AAACCAACAC GGCATTCGGC AGTGCCTTTC

1441 TTTCACAACA CCGCTTACAT AATCAACATC ATCCAAGGGG CCGCGGCACG CCTAATCAAG

1501 CAGCTACCTG TGCTCGCATG CGCGCTATCT AAGGCACCAC TCGGTGTCAT CCCCACTTGC

1561 AAAGGATCTA AACCCAAACC CTAACATCGC AGACTGCAGA TGCGCACACA TACTACATAA

1621 GTGGGTTCTT CCGGACAACT TTAAAAGCTC ACACTCTTGT ATTGAATCTC CAAATAACTT

1681 CGTATAATGT ATGCTATACG AAGTTATCAC GGGCAAAAGT GCGTATATAT ACAAGAGCGT

1741 TTGCCAGCCA CAGATTTTCA CTCCACACAC CACATCACAC ATACAACCAC ACACATCCAC

1801 AATGGAACCC GAAACTAAGA TGAAAAAGCC TGAACTCACC GCGACGTCTG TCGAGAAGTT

1861 TCTGATCGAA AAGTTCGACA GCGTCTCCGA CCTGATGCAG CTCTCGGAGG GCGAAGAATC

1921 TCGTGCTTTC AGCTTCGATG TAGGAGGGCG TGGATATGTC CTGCGGGTAA ATAGCTGCGC

1981 CGATGGTTTC TACAAAGATC GTTATGTTTA TCGGCACTTT GCATCGGCCG CGCTCCCGAT

2041 TCCGGAAGTG CTTGACATTG GGGAGTTCAG CGAGAGCCTG ACCTATTGCA TCTCCCGCCG

2101 TGCACAGGGT GTCACGTTGC AAGACCTGCC TGAAACCGAA CTGCCCGCTG TTCTACAGCC

2161 GGTCGCGGAG GCAATGGATG CGATCGCTGC GGCCGATCTT AGCCAGACGA GCGGGTTCGG

2221 CCCATTCGGA CCGCAAGGAA TCGGTCAATA CACTACATGG CGTGATTTCA TTTGCGCGAT

2281 TGCTGATCCC CATGTGTATC ACTGGCAAAC TGTGATGGAC GACACCGTCA GTGCGTCCGT

2341 CGCGCAGGCT CTCGATGAGC TGATGCTTTG GGCCGAGGAC TGCCCCGAAG TCCGGCACCT

2401 CGTGCACGCG GATTTCGGCT CCAACAATGT CCTGACGGAC AATGGCCGCA TAACAGCGGT

2461 CATTGACTGG AGCGAGGCGA TGTTCGGGGA TTCCCAATAC GAGGTCGCCA ACATCTTCTT

2521 CTGGAGGCCG TGGTTGGCTT GTATGGAGCA GCAGACGCGC TACTTCGAGC GGAGGCATCC

2581 GGAGCTTGCA GGATCGCCGC GACTCCGGGC GTATATGCTC CGCATTGGTC TTGACCAACT

2641 CTATCAGAGC TTGGTTGACG GCAATTTCGA TGATGCAGCT TGGGCGCAGG GTCGATGCGA

2701 CGCAATCGTC CGATCCGGAG CCGGGACTGT CGGGCGTACA CAAATCGCCC GCAGAAGCGC

2761 GGCCGTCTGG ACCGATGGCT GTGTAGAAGT ACTCGCCGAT AGTGGAAACC GACGCCCCAG

2821 CACTCGTCCG AGGGCAAAGG AATAGGACTC TATAAAAAGG GCCCTGCCCT GCTAATGAAA

2881 TGATGATTTA TAATTTACCG GTGTAGCAAC CTTGACTAGA AGAAGCAGAT TGGGTGTGTT

2941 TGTAGTGGAG GACAGTGGTA CGTTTTGGAA ACAGTCTTCT TGAAAGTGTC TTGTCTACAG

3001 TATATTCACT CATAACCTCA ATAGCCAAGG GTGTAGTCGG TTTATTAAAG ATAACTTCGT

3061 ATAATGTATG CTATACGAAG TTATAACATC TATACTGTAA GAAAATATCG ATTTTTGTAA

3121 TAGCGAGTTG AATCTATTAT GTTGAAATAA GTTATGCTAT AAATAGTTGA GTTGTGAGCG

3181 GGTTGTTGAA GGGCCGCTTG GCGTAGGCCG TTTTCTGTTG CTTATCAGTA AATACCTAAT

3241 AGAGGCATTT TACTTCTGCT TGGCAGCGAG CTCCCGGTCT CGCTTAGCGT AGATCTCACC

3301 GGTGAAGGGT TCCTCGAACT TGAGAGCAAT GGAGTACATG AGCGACACAA AGGCGGTGAT

3361 GAGCAGGCCT GCGGGCTTGG CGTACCACAG AGCGGAAGCC AGGAAACACA GAGTGGAGCC

3421 GTGGTACATG GGGTTGTTGG ACACATTGAA GGGGAAGCCG GTGACTCGCT CGTTCATGAG

3481 AATGCCAAAG TAGTCGCCGA GGTAGGTTCC GGTGACGCCC AGGGCGTACA TGGAGGTAAG

3541 GACCAGCACG TTGCCAACAG CAAACAGCAC AATGGCCAGA GGCTTGACAA TGTCAACGTA

3601 GGTGACCGAC AGCAGAATGG AGGTGGGCTG CTGCTCAAGA GCCTGGTGGT AAAGGTAGTC

3661 TCGGAAGATA CCGAGCGAGA AAATGGTGAT GGCCAGCAGA TAGCATCCGG TCTTAGCTCC

3721 TCCAGCCAGC TTAGTCAGAA AGTGGGTGTT GTACTCGGCT CGAGCAACAA TGTTCCAAAA

3781 GATGGGGTTG AAGGCGATGG AGCCCAGGAC GACTGTGTTA GTTATGTTTA AGGGAGGGTT

3841 GTGGCCCTGG CGTGGGGTGC TAGATCGGTG GCTAGGGGAT TAGTTCCATC AGCTTCTTAC

3901 ACCGTGTACA CATGATTGTG GGGTACAAGT GTGTGTAACA GGGGGCCGAG GGACGCCAAT

3961 GGTTGCGGTA CTCACTCCAG AGGCTCTTCT GGTCGTAATC CACGTTGAAG TCGGCAACAA

4021 TCTTGTCAGG AAAGGACATT GTAATTTCTG GTCTTCGGAA ATCTGATTCT AAGAGAGTTT

4081 TCCAGAGAGC CAACCAGCTC GGACAGCTTC ACATGCGCGG TTAGGGTTGA CATTTCACAT

4141 GACGCGTCTG AGCTGTGCGT GGGCTGAAGT ACACACTTGT TGGGTGAAGG GGGAGAGGCC

4201 ACTGGTTGTG TATTAATATT GTTAGAAGTT GTACTGTAGC AGCCATTCAA TTAGTGTCCG

4261 TTTCTGAATC AAGTCACTTT CGAAAGAAAT AAAAAAGCCA AAGAAACTCG GCCACACTAA

4321 GGGAGAGTTT CGAGATTTCA TCGAACTAAC ATAAATGAAT GTATATGCAG AAAAAAAAAA

4381 ACAGAAGTTT GCGAGAAGAC GGATTTGGCC AATGAGCGGT ATTTGCGGGA GTTGTAATAT

4441 CCGTCTGAAT CAGCTCAACG AGAGGGGTTT AATGGTGTTA TAATAACTTA TCTTTTTCTC

4501 GCGGATCGAA TCATTGATTG ACCGTTCAGT CTTGCCAGAT CATGTATCTG ATGCGGCTAA

4561 CAGAAGATAA TTCTGCAAGC TCAAGCGGCC GCAGAGACGG AGTCACTGCC AACCGAGACG

4621 GTCATAGCTG TTTCCTGTGT GCCGCTTCCT CGCTCACTGA CTCGCTGCGC TCGGTCGTTC

4681 GGCTGCGGCG AGCGGTATCA GCTCACTCAA AGGCGGTAAT ACGGTTACCC ACAGAATCAG

4741 GGGATAACGC AGGAAAGAAC ATGTGAGCAA AAGGCCAGCA AAAGGCCAGG AACCGTAAAA

4801 AGGCCGCGTT GCTGGCGTTT TTCCATAGGC TCCGCCCCCC TGACGAGCAT CACAAAAATC

4861 GACGCTCAAG TCAGAGGTGG CGAAACCCGA CAGGACTATA AAGATACCAG GCGTTTCCCC

4921 CTGGAAGCTC CCTCGTGCGC TCTCCTGTTC CGACCCTGCC GCTTACCGGA TACCTGTCCG

4981 CCTTTCTCCC TTCGGGAAGC GTGGCGCTTT CTCAATGCTC ACGCTGTAGG TATCTCAGTT

5041 CGGTGTAGGT CGTTCGCTCC AAGCTGGGCT GTGTGCACGA ACCCCCCGTT CAGCCCGACC

5101 GCTGCGCCTT ATCCGGTAAC TATCGTCTTG AGTCCAACCC GGTAAGACAC GACTTATCGC

5161 CACTGGCAGC AGCCACTGGT AACAGGATTA GCAGAGCGAG GTATGTAGGC GGTGCTACAG

5221 AGTTCTTGAA GTGGTGGCCT AACTACGGCT ACACTAGAAG GACAGTATTT GGTATCTGCG

5281 CTCTGCTGAA GCCAGTTACC TTCGGAAAAA GAGTTGGTAG CTCTTGATCC GGCAAACAAA

5341 CCACCGCTGG TAGCGGTGGT TTTTTTGTTT GCAAGCAGCA GATTACGCGC AGAAAAAAAG

5401 GATCTCAAGA AGATCCTTTG ATCTTTTCTA CGGGGTCTGA CGCTCAGTGG AACGAAAACT

5461 CACGTTAAGG GATTTTGGTC ATGAGATTAT CAAAAAGGAT CTTCACCTAG ATCCTTTTAA

5521 ATTAAAAATG AAGTTTTAAA TCAATCTAAA GTATATATGA GTAAACTTGG TCTGACAGTT

5581 ACCAATGCTT AATCAGTGAG GCACCTATCT CAGCGATCTG TCTATTTCGT TCATCCATAG

5641 TTGCCTGACT CCCCGTCGTG TAGATAACTA CGATACGGGA GGGCTTACCA TCTGGCCCCA

5701 GTGCTGCAAT AATACCGCGG GACCCACGCT CACCGGCTCC AGATTTATCA GCAATAAACC

5761 AGCCAGCCGG AAGGGCCGAG CGCAGAAGTG GTCCTGCAAC TTTATCCGCC TCCATCCAGT

5821 CTATTAATTG TTGCCGGGAA GCTAGAGTAA GTAGTTCGCC AGTTAATAGT TTGCGCAACG

5881 TTGTTGCCAT CGCTACAGGC ATCGTGGTGT CACGCTCGTC GTTTGGTATG GCTTCATTCA

5941 GCTCCGGTTC CCAACGATCA AGGCGAGTTA CATGATCCCC CATGTTGTGC AAAAAAGCGG

6001 TTAGCTCCTT CGGTCCTCCG ATCGTTGTCA GAAGTAAGTT GGCCGCCGTG TTATCACTCA

6061 TGGTTATGGC AGCACTACAT AATTCTCTTA CTGTCATGCC ATCCGTAAGA TGCTTTTCTG

6121 TGACTGGTGA GTACTCAACC AAGTCATTCT GAGAATAGTG TATGCGGCGA CCGAGTTGCT

6181 CTTGCCCGGC GTCAATACGG GATAATACCG CGCCACATAG CAGAACTTTA AAAGTGCTCA

6241 TCATTGGAAA ACGTTCTTCG GGGCGAAAAC TCTCAAGGAT CTTACCGCTG TTGAGATCCA

6301 GTTCGATGTA ACCCACTCGT GCACCCAACT GATCTTCAGC ATCTTTTACT TTCACCAGCG

6361 TTTCTGGGTG AGCAAAAACA GGAAGGCAAA ATGCCGCAAA AAAGGGAATA AGGGCGACAC

6421 GGAAATGTTG AATACTCATA CTCTTCCTTT TTCAATATTA TTGAAGCATT TATCAGGGTT

6481 ATTGTCTCAT GAGCGGATAC ATATTTGAAT GTATTTAG


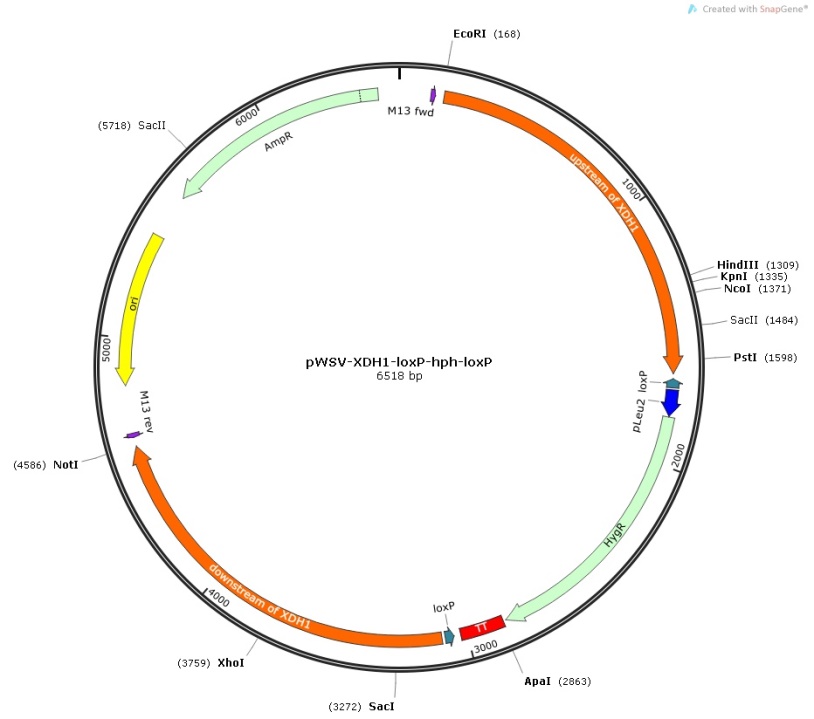


**Sequence 8: The *EYD1* gene knockout vector**

1-167: pUC19 derived vector sequence

168-1673: upstream sequence of EYD1 gene

1674-1708: loxP sequence

1709-1820: minimal promoter of Leu2 gene

1821-2846: hph gene

2847-3051: terminator sequence

3052-3085: loxP sequence

3086-4585: downstream sequence of EYD1 gene

4586-6518: pUC19 derived vector sequence

**The *EYD1* gene knockout cassette fragment from 168 bp to 4585 bp was synthesized in vitro.**

1 AAAAATAAAC AAATAGGGGT TCCGCGCACA TTTCCCCGAA AAGTGCCACC TGACGTCTAA

61 GAAACCATTA TTATCATGAC ATTAACCTAT AAAAATAGGC GTATCACGAG GCCCTTTCGT

121 TGTAAAACGA CGGCCAGTCG TCTCGATCCG CAGTGTCTTG CGTCTCTGAA TTCCACCTTT

181 CTGCCCCAAA GTACTTGTAC AAAAATAGAC CCCAAATTGC ACCTTTCAAA CCTCCAACAT

241 CTTCACCCAC ATCTCTCTGA CCCCAACTCA CCGCACCATC GCTGATGCGT CTCAACGGCA

301 CACTCTAGGT TTCGTTTGTG GGGCGATGAG TGTCCCCTCA TCATCTGCAC GCTCTTTCAA

361 GTGTCTCCCC TCTTGGCGGC TGGCGTTCCG GGCTGTGGAA GATTCCAGAA GTGCGGAAGT

421 GCAGTAAGCG GGACGAGGGA CTTTCTCGGA GCTCTGTCAC GTGAATCTCT CGGCCGAAGC

481 GTCACGTGGC AGAATTTATG CGTGTCCTCT TCTTCCTGTG ACGGATCGAC ATTGCCACGA

541 TGCTACAAAA AGAGTAGCGG CAAAAGGAGT AAGTGGTCAA CAGCCATGTA GCTTAGCTCA

601 CAAAAAGAAG AGAGCAAAGA ATCGAGGAAC GGAATCGAAG GATGAAGAGC AGAGCAACGT

661 AGTGTAGAGA GATGACTTCC GTATCACGAG CAAGCAAACA TCTTCACTCT CCAAGAGCTG

721 CCACTGTAGC ATCAACATGA GACATGGCAA GTATTATGCA TGGTGCACTT GTAACATAGC

781 CCCCAGATCA GGGATATTCT GAAACTAGAG CCATCTCAAC ACAACAGTCT CTTTGTGTAG

841 CTACTTGTAC CCTTTTTCTC TTCCTCTCTC CAGCCAGACA TCTTTGCTAG CGCCTATAAT

901 GTAACCCATC AAGACATGCA CAGGAGATGC TTAATCGGAG TGTGTGGTCT GTAGGGGAGA

961 TCGAGAGAGA CTGCAATTGA CAGAGAGATC GAAGTTGGAA TGAGAGAGAC TGAAAATTAA

1021 GCGAGCTTGG GTGTTTGCCC CTCCCCTCAC ACCCTCGGAT ACTGTACCTA CATATCCAGG

1081 CCGGTTTGGC ACGGCATCAA AAGCCTCCTA CAAGAATGTA TATGCAACTC TTCTACAAGT

1141 AGATTTCCGC GCTTGCACCA ACGGCTACGC CCAAGACGGG GCTCGTACCC GTCCGTCTAT

1201 GGTTCAGCCG CCAACGAAAA AAAAAAAAAA GGATGGCTGT AATTTTATTA TGCTTCTGTG

1261 TTTGTGTTTG TCGGTCCGTT TTTGCTTTTT TCACCCCCAG GCTGTTATTC CGGGGAATAA

1321 GGCTGGTCAT GATGGGGTTG GAAAGTCTAA ATTTTTGTGG GACAAAGAAA GCAGGTATCG

1381 TGCCACTAAG AAAATAGACT TTTAGGCACC CCAGATTTTT GGAAACCTTA ATAGGAGACT

1441 ACTTCCGTTT CCTAATTAGG ACTTCCGCGA CCCCAGACAA AGCGGCTTGG AGTAGGCCTC

1501 GTGTCCGGCC TAGGGCAGAA ACAGCTCCGG AACTCGATTG AGAAGCCGTA CTCTGGAAAG

1561 TCTAGAGGAA GTTCCAAGGT CGAGTCTCTT CGATATAAAA GGACGCCATG GAAGCTCTGT

1621 AGTTCGATAT CAAATACTGA CAACAGTTTC CAAACACACA AACACACACA CACATAACTT

1681 CGTATAATGT ATGCTATACG AAGTTATCAC GGGCAAAAGT GCGTATATAT ACAAGAGCGT

1741 TTGCCAGCCA CAGATTTTCA CTCCACACAC CACATCACAC ATACAACCAC ACACATCCAC

1801 AATGGAACCC GAAACTAAGA TGAAAAAGCC TGAACTCACC GCGACGTCTG TCGAGAAGTT

1861 TCTGATCGAA AAGTTCGACA GCGTCTCCGA CCTGATGCAG CTCTCGGAGG GCGAAGAATC

1921 TCGTGCTTTC AGCTTCGATG TAGGAGGGCG TGGATATGTC CTGCGGGTAA ATAGCTGCGC

1981 CGATGGTTTC TACAAAGATC GTTATGTTTA TCGGCACTTT GCATCGGCCG CGCTCCCGAT

2041 TCCGGAAGTG CTTGACATTG GGGAGTTCAG CGAGAGCCTG ACCTATTGCA TCTCCCGCCG

2101 TGCACAGGGT GTCACGTTGC AAGACCTGCC TGAAACCGAA CTGCCCGCTG TTCTACAGCC

2161 GGTCGCGGAG GCAATGGATG CGATCGCTGC GGCCGATCTT AGCCAGACGA GCGGGTTCGG

2221 CCCATTCGGA CCGCAAGGAA TCGGTCAATA CACTACATGG CGTGATTTCA TTTGCGCGAT

2281 TGCTGATCCC CATGTGTATC ACTGGCAAAC TGTGATGGAC GACACCGTCA GTGCGTCCGT

2341 CGCGCAGGCT CTCGATGAGC TGATGCTTTG GGCCGAGGAC TGCCCCGAAG TCCGGCACCT

2401 CGTGCACGCG GATTTCGGCT CCAACAATGT CCTGACGGAC AATGGCCGCA TAACAGCGGT

2461 CATTGACTGG AGCGAGGCGA TGTTCGGGGA TTCCCAATAC GAGGTCGCCA ACATCTTCTT

2521 CTGGAGGCCG TGGTTGGCTT GTATGGAGCA GCAGACGCGC TACTTCGAGC GGAGGCATCC

2581 GGAGCTTGCA GGATCGCCGC GACTCCGGGC GTATATGCTC CGCATTGGTC TTGACCAACT

2641 CTATCAGAGC TTGGTTGACG GCAATTTCGA TGATGCAGCT TGGGCGCAGG GTCGATGCGA

2701 CGCAATCGTC CGATCCGGAG CCGGGACTGT CGGGCGTACA CAAATCGCCC GCAGAAGCGC

2761 GGCCGTCTGG ACCGATGGCT GTGTAGAAGT ACTCGCCGAT AGTGGAAACC GACGCCCCAG

2821 CACTCGTCCG AGGGCAAAGG AATAGGACTC TATAAAAAGG GCCCTGCCCT GCTAATGAAA

2881 TGATGATTTA TAATTTACCG GTGTAGCAAC CTTGACTAGA AGAAGCAGAT TGGGTGTGTT

2941 TGTAGTGGAG GACAGTGGTA CGTTTTGGAA ACAGTCTTCT TGAAAGTGTC TTGTCTACAG

3001 TATATTCACT CATAACCTCA ATAGCCAAGG GTGTAGTCGG TTTATTAAAG ATAACTTCGT

3061 ATAATGTATG CTATACGAAG TTATGTTAAC TTATTGCATT TAATATTATG ATTATGATTC

3121 GAAAACACTA GGAACGTTGT ATTTGTAGAA CTTCACCAGT AATCAATGTA GGAGTACGAG

3181 TAATAGAATA ATCTCGGGAC AGCAATGTAA GAGACTTACA CATTTCAGAA TTGATGAAAT

3241 TTGCACTTGG AACGAAGTCC CTCGAACTGC AACCGCTATT TGCACGAACT CGCCGCTTCC

3301 TGAAAGACGC TGAAGACTCA ACTTGCTAAA CTGAGAATTA ATACCGTTTC TTGAAATCTA

3361 CTTGTAGTTA CTCTTTATTG CACTCTGTAG CAGGAAAGGA TTAATACAAC TACTTGTAGT

3421 CCTCTGAACG CGAATCACCT CACACCATCT AAGACAAGTG TGCAGATAAT TGCATCAAGT

3481 AAGCCCTACA CAACGGTGGA GGAAAAAGCA ACCCGACCAG GCAGAGGGCC AGGAACACTC

3541 CGACAGTCTC GGACGCTTCT GGGAGTCTCT TACACATGAA AAATCTGATT CACCGGGAGT

3601 GCACGTTGCC CCAGATTTTT GTTTTTCATT TCACTGCCTT ACCATGTCCG GAGACGAGAA

3661 ACTTTTGCGG AAGACCCTTT CGGGAAGTCT CTATGCACCA CACACCGCTT TAGAGGTCAC

3721 GATATTTGAA AATGTGTTTC GGATCTACTG TATGTACATC TTAGAAACTC GCTCGGGTGA

3781 ACAAATTTCA GCCCCGGATA TTACCGACCC GTACCCGAGA TTGTACTGTT GTCCAACATA

3841 GGTTACATCG GGATGTTCCG TACAGCCCGC GCACTTTGAA AGCACATGCT ACGTTAGGGG

3901 GGATTTATGG AGCAAATTGC GGTGTTCTGT TACTGTTGTA CAACTCGTGA GCGGAATCTC

3961 TCTATTGAGA GTCGTGCTCA TACCGAATAA TCGATTTGCT TCCAATTTCA CTTACAGAGC

4021 ACATGAATCT TTGCCACCAG GAGATCTGCA TCTTTAGTAA GAAAAAGACA GTATCGTCGT

4081 CACACGTCAC ATGACCTCAA ACCTGTTGTC GACACAGCTG TCGATCAGCA CGTGGTCTCT

4141 CCAGCTTCCC GCTGAAGATT AGTTTCCCCG TTGGGAAATC TTTTTGATTC CATCTTGTTC

4201 GTCTCCTCTC TCTTTTTCTA AATTAAGAAG GTTCCGGAAC TGAGACGGGG TCGTACCATC

4261 TCTGAGACAC TAAAGGCTGA AGAAGTGGGA CTAGAACTGC TCGAAAGCAT CACGGAGCGC

4321 GCTGGAATCT AATTTCCATG TCTTTAGATA CCGCCCATTC CATAATTTGC ATGACGACAG

4381 CTCATTTAGT ACCCCACCTC TCCGCATTGT TATAGCCGTA TGTTTTTCCG ACATACGAAC

4441 AAATAAGCTC TTAGAGTTAC GGTTCGCTTC TTTGGGTGTC CGTTGACAAA GACACCATGA

4501 GGCGGGGAAT AAAAACTTAG ATACGATAGA GTTCATATGC ACCATGCGTT AAATCGAACC

4561 TTACGCGTAG TAAACTGTGG AGAAGCGGCC GCAGAGACGG AGTCACTGCC AACCGAGACG

4621 GTCATAGCTG TTTCCTGTGT GCCGCTTCCT CGCTCACTGA CTCGCTGCGC TCGGTCGTTC

4681 GGCTGCGGCG AGCGGTATCA GCTCACTCAA AGGCGGTAAT ACGGTTACCC ACAGAATCAG

4741 GGGATAACGC AGGAAAGAAC ATGTGAGCAA AAGGCCAGCA AAAGGCCAGG AACCGTAAAA

4801 AGGCCGCGTT GCTGGCGTTT TTCCATAGGC TCCGCCCCCC TGACGAGCAT CACAAAAATC

4861 GACGCTCAAG TCAGAGGTGG CGAAACCCGA CAGGACTATA AAGATACCAG GCGTTTCCCC

4921 CTGGAAGCTC CCTCGTGCGC TCTCCTGTTC CGACCCTGCC GCTTACCGGA TACCTGTCCG

4981 CCTTTCTCCC TTCGGGAAGC GTGGCGCTTT CTCAATGCTC ACGCTGTAGG TATCTCAGTT

5041 CGGTGTAGGT CGTTCGCTCC AAGCTGGGCT GTGTGCACGA ACCCCCCGTT CAGCCCGACC

5101 GCTGCGCCTT ATCCGGTAAC TATCGTCTTG AGTCCAACCC GGTAAGACAC GACTTATCGC

5161 CACTGGCAGC AGCCACTGGT AACAGGATTA GCAGAGCGAG GTATGTAGGC GGTGCTACAG

5221 AGTTCTTGAA GTGGTGGCCT AACTACGGCT ACACTAGAAG GACAGTATTT GGTATCTGCG

5281 CTCTGCTGAA GCCAGTTACC TTCGGAAAAA GAGTTGGTAG CTCTTGATCC GGCAAACAAA

5341 CCACCGCTGG TAGCGGTGGT TTTTTTGTTT GCAAGCAGCA GATTACGCGC AGAAAAAAAG

5401 GATCTCAAGA AGATCCTTTG ATCTTTTCTA CGGGGTCTGA CGCTCAGTGG AACGAAAACT

5461 CACGTTAAGG GATTTTGGTC ATGAGATTAT CAAAAAGGAT CTTCACCTAG ATCCTTTTAA

5521 ATTAAAAATG AAGTTTTAAA TCAATCTAAA GTATATATGA GTAAACTTGG TCTGACAGTT

5581 ACCAATGCTT AATCAGTGAG GCACCTATCT CAGCGATCTG TCTATTTCGT TCATCCATAG

5641 TTGCCTGACT CCCCGTCGTG TAGATAACTA CGATACGGGA GGGCTTACCA TCTGGCCCCA

5701 GTGCTGCAAT AATACCGCGG GACCCACGCT CACCGGCTCC AGATTTATCA GCAATAAACC

5761 AGCCAGCCGG AAGGGCCGAG CGCAGAAGTG GTCCTGCAAC TTTATCCGCC TCCATCCAGT

5821 CTATTAATTG TTGCCGGGAA GCTAGAGTAA GTAGTTCGCC AGTTAATAGT TTGCGCAACG

5881 TTGTTGCCAT CGCTACAGGC ATCGTGGTGT CACGCTCGTC GTTTGGTATG GCTTCATTCA

5941 GCTCCGGTTC CCAACGATCA AGGCGAGTTA CATGATCCCC CATGTTGTGC AAAAAAGCGG

6001 TTAGCTCCTT CGGTCCTCCG ATCGTTGTCA GAAGTAAGTT GGCCGCCGTG TTATCACTCA

6061 TGGTTATGGC AGCACTACAT AATTCTCTTA CTGTCATGCC ATCCGTAAGA TGCTTTTCTG

6121 TGACTGGTGA GTACTCAACC AAGTCATTCT GAGAATAGTG TATGCGGCGA CCGAGTTGCT

6181 CTTGCCCGGC GTCAATACGG GATAATACCG CGCCACATAG CAGAACTTTA AAAGTGCTCA

6241 TCATTGGAAA ACGTTCTTCG GGGCGAAAAC TCTCAAGGAT CTTACCGCTG TTGAGATCCA

6301 GTTCGATGTA ACCCACTCGT GCACCCAACT GATCTTCAGC ATCTTTTACT TTCACCAGCG

6361 TTTCTGGGTG AGCAAAAACA GGAAGGCAAA ATGCCGCAAA AAAGGGAATA AGGGCGACAC

6421 GGAAATGTTG AATACTCATA CTCTTCCTTT TTCAATATTA TTGAAGCATT TATCAGGGTT

6481 ATTGTCTCAT GAGCGGATAC ATATTTGAAT GTATTTAG


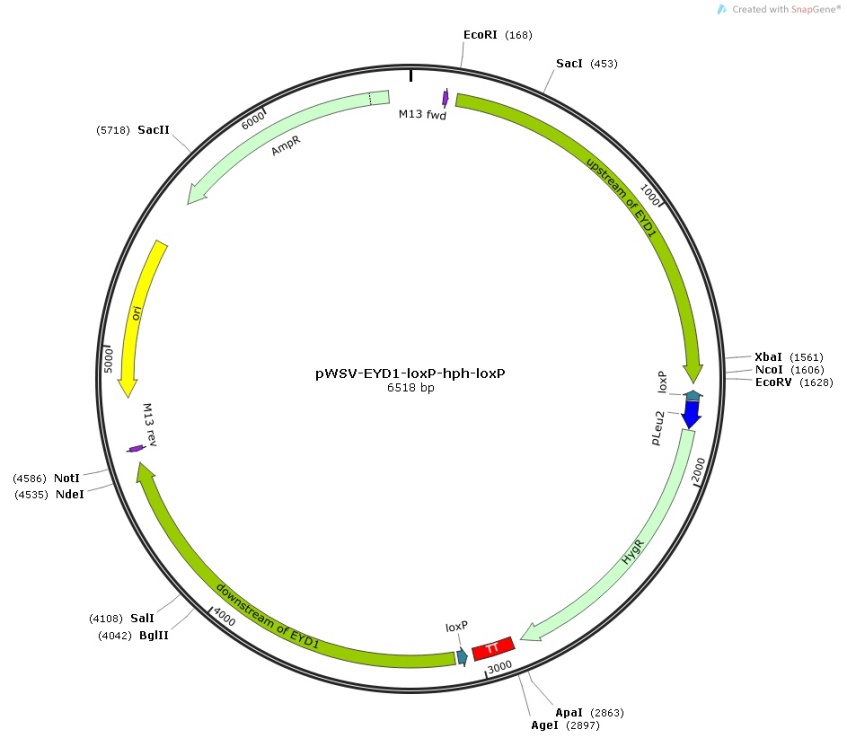


**Sequence 9: zwf1-gnd1 expression cassette**

1-8: NotI

9-586: 26S rDNA

587-1118：hp4d promoter

1119-2615:Y. lipolytica zwf1 gene

2616-2873: terminator

2874-2907: loxP

2908-4250: hph marker cassette

4251-4284:loxP

4285-4816:hp4d promoter

4817-6286:Y. lipolytica gnd1 gene

6287-6601:terminator

6602-7204:26S rDNA

7205-7212:NotI

1 GCGGCCGCCA GATCTTGGTG GTAGTAGCAA ATATTCAAAT GAGAACTTTG AAGACTGAAG

61 TGGGGAAAGG TTCCGTGTGA ACAGCAGTTG GACACGGGTA AGTCGATCCT AAGGGGTGGC

121 ATAACTGTCG CGTACGGCCC GATAAGGGCC TTCTCCAAAA GGGAAGCCGG TTGAAATTCC

181 GGCACTTGGA TGTGGATTCT CCACGGCAAC GTAACTGAAT GTGGGGACGG TGGCACAAGT

241 CTTGGAAGGA GTTATCTTTT CTTTTTAACG GAGTCAACAC CCTGGAATTA GTTTGTCTAG

301 AGATAGGGTA TCGTTCCGGA AGAGGGGGGC AGCTTTGTCC CCTCCGATGC ACTTGTGACG

361 CCCCTTGAAA ACCCGCAGGA AGGAATAGTT TTCACGCCAA GTCGTACTGA TAACCGCAGC

421 AGGTCTCCAA GGTGAACAGC CTCTAGTTGA TAGAATAATG TAGATAAGGG AAGTCGGCAA

481 AATAGATCCG TAACTTCGGG ATAAGGATTG GCTCTGGGGG TTGGTGGATG GAAGCGTGGG

541 AGACCCCAAG GGACTGGCAG CTGGGCAACT GGCAGCCGGA CCCGCCTGAG GTGTCTCACA

601 AGTGCCGTGC AGTCCCGCCC CCACTTGCTT CTCTTTGTGT GTAGTGTACG TACATTATCG

661 AGACCGTTGT TCCCGCCCAC CTCGATCCGG CTGAGGTGTC TCACAAGTGC CGTGCAGTCC

721 CGCCCCCACT TGCTTCTCTT TGTGTGTAGT GTACGTACAT TATCGAGACC GTTGTTCCCG

781 CCCACCTCGA TCCGGCTGAG GTGTCTCACA AGTGCCGTGC AGTCCCGCCC CCACTTGCTT

841 CTCTTTGTGT GTAGTGTACG TACATTATCG AGACCGTTGT TCCCGCCCAC CTCGATCCGG

901 CTGAGGTGTC TCACAAGTGC CGTGCAGTCC CGCCCCCACT TGCTTCTCTT TGTGTGTAGT

961 GTACGTACAT TATCGAGACC GTTGTTCCCG CCCACCTCGA TCCGGCACGG GCAAAAGTGC

1021 GTATATATAC AAGAGCGTTT GCCAGCCACA GATTTTCACT CCACACACCA CATCACACAT

1081 ACAACCACAC ACATCCACAA TGGAACCCGA AACTAAGHPD ATGACTGGCA CCTTACCCAA

1141 GTTCGGCGAC GGAACCACCA TTGTGGTTCT TGGAGCCTCC GGCGACCTCG CTAAGAAGAA

1201 GACCTTCCCC GCCCTCTTCG GCCTTTACCG AAACGGCCTG CTGCCCAAAA ATGTTGAAAT

1261 CATCGGCTAC GCACGGTCGA AAATGACTCA GGAGGAGTAC CACGAGCGAA TCAGCCACTA

1321 CTTCAAGACC CCCGACGACC AGTCCAAGGA GCAGGCCAAG AAGTTCCTTG AGAACACCTG

1381 CTACGTCCAG GGACCTTACG ACGGTGCCGA GGGCTACCAG CGACTGAATG AAAAGATTGA

1441 GGAGTTTGAG AAGAAGAAGC CCGAGCCCCA CTACCGTCTT TTCTACCTGG CTCTGCCCCC

1501 CAGCGTCTTC CTTGAGGCTG CCAACGGTCT GAAGAAGTAT GTCTACCCCG GCGAGGGCAA

1561 GGCCCGAATC ATCATCGAGA AGCCCTTTGG CCACGACCTG GCCTCGTCAC GAGAGCTGCA

1621 GGACGGCCTT GCTCCTCTCT GGAAGGAGTC TGAGATCTTC CGAATCGACC ACTACCTCGG

1681 AAAGGAGATG GTCAAGAACC TCAACATTCT GCGATTTGGC AACCAGTTCC TGTCCGCCGT

1741 GTGGGACAAG AACACCATTT CCAACGTCCA GATCTCCTTC AAGGAGCCCT TTGGCACTGA

1801 GGGCCGAGGT GGATACTTCA ACGACATTGG AATCATCCGA GACGTTATTC AGAACCATCT

1861 GTTGCAGGTT CTGTCCATTC TAGCCATGGA GCGACCCGTC ACTTTCGGAG CCGAGGACAT

1921 TCGAGATGAG AAGGTCAAGG TGCTCCGATG TGTGGACATT CTCAACATTG ACGACGTCAT

1981 TCTCGGCCAG TACGGCCCCT CTGAAGACGG AAAGAAGCCC GGATACACCG ATGACGATGG

2041 CGTTCCCGAT GACTCCCGAG CTGTGACCTT TGCTGCTCTC CATCTCCAGA TCCACAACGA

2101 CAGATGGGAG GGTGTTCCTT TCATCCTCCG AGCCGGTAAG GCTCTGGACG AGGGCAAGGT

2161 CGAGATCCGA GTGCAGTTCC GAGACGTGAC CAAGGGCGTT GTGGACCATC TGCCTCGAAA

2221 TGAGCTGGTC ATCCGAATCC AGCCCTCCGA GTCCATCTAC ATGAAGATGA ACTCCAAGCT

2281 GCCTGGCCTT ACTGCCAAGA ACATTGTCAC CGACCTGGAT CTGACCTACA ACCGACGATA

2341 CTCGGACGTG CGAATCCCTG AGGCTTACGA GTCTCTCATT CTGGACTGCC TCAAGGGTGA

2401 CCACACCAAC TTTGTGCGAA ACGACGAGCT GGACATTTCC TGGAAGATTT TCACCGATCT

2461 GCTGCACAAG ATTGACGAGG ACAAGAGCAT TGTGCCCGAG AAGTACGCCT ACGGCTCTCG

2521 TGGCCCCGAG CGACTCAAGC AGTGGCTCCG AGACCGAGGC TACGTGCGAA ACGGCACCGA

2581 GCTGTACCAA TGGCCTGTCA CCAAGGGCTC CTCGTGAGCA ATTAACAGAT AGTTTGCCGG

2641 TGATAATTCT CTTAACCTCC CACACTCCTT TGACATAACG ATTTATGTAA CGAAACTGAA

2701 ATTTGACCAG ATATTGTTGT AAATAGAAAA TCTGGCTTGT AGGTGGCAAA ATCCCGTCTT

2761 TGTTCGTCGG TTCCCTCTGT GACTGCTCGT CGTCCCTTTG TGTTCGACTG TCGTGTTTTG

2821 TTTTCCGTGC GTGCGCAAGT GAGATGCCCG TGTTCGAATT GGGTAGTCGC ACGGAATAAC

2881 TTCGTATAAT GTATGCTATA CGAAGTTATC ACGGGCAAAA GTGCGTATAT ATACAAGAGC

2941 GTTTGCCAGC CACAGATTTT CACTCCACAC ACCACATCAC ACATACAACC ACACACATCC

3001 ACAATGGAAC CCGAAACTAA GATGAAAAAG CCTGAACTCA CCGCGACGTC TGTCGAGAAG

3061 TTTCTGATCG AAAAGTTCGA CAGCGTCTCC GACCTGATGC AGCTCTCGGA GGGCGAAGAA

3121 TCTCGTGCTT TCAGCTTCGA TGTAGGAGGG CGTGGATATG TCCTGCGGGT AAATAGCTGC

3181 GCCGATGGTT TCTACAAAGA TCGTTATGTT TATCGGCACT TTGCATCGGC CGCGCTCCCG

3241 ATTCCGGAAG TGCTTGACAT TGGGGAGTTC AGCGAGAGCC TGACCTATTG CATCTCCCGC

3301 CGTGCACAGG GTGTCACGTT GCAAGACCTG CCTGAAACCG AACTGCCCGC TGTTCTACAG

3361 CCGGTCGCGG AGGCAATGGA TGCGATCGCT GCGGCCGATC TTAGCCAGAC GAGCGGGTTC

3421 GGCCCATTCG GACCGCAAGG AATCGGTCAA TACACTACAT GGCGTGATTT CATTTGCGCG

3481 ATTGCTGATC CCCATGTGTA TCACTGGCAA ACTGTGATGG ACGACACCGT CAGTGCGTCC

3541 GTCGCGCAGG CTCTCGATGA GCTGATGCTT TGGGCCGAGG ACTGCCCCGA AGTCCGGCAC

3601 CTCGTGCACG CGGATTTCGG CTCCAACAAT GTCCTGACGG ACAATGGCCG CATAACAGCG

3661 GTCATTGACT GGAGCGAGGC GATGTTCGGG GATTCCCAAT ACGAGGTCGC CAACATCTTC

3721 TTCTGGAGGC CGTGGTTGGC TTGTATGGAG CAGCAGACGC GCTACTTCGA GCGGAGGCAT

3781 CCGGAGCTTG CAGGATCGCC GCGACTCCGG GCGTATATGC TCCGCATTGG TCTTGACCAA

3841 CTCTATCAGA GCTTGGTTGA CGGCAATTTC GATGATGCAG CTTGGGCGCA GGGTCGATGC

3901 GACGCAATCG TCCGATCCGG AGCCGGGACT GTCGGGCGTA CACAAATCGC CCGCAGAAGC

3961 GCGGCCGTCT GGACCGATGG CTGTGTAGAA GTACTCGCCG ATAGTGGAAA CCGACGCCCC

4021 AGCACTCGTC CGAGGGCAAA GGAATAGGAC TCTATAAAAA GGGCCCTGCC CTGCTAATGA

4081 AATGATGATT TATAATTTAC CGGTGTAGCA ACCTTGACTA GAAGAAGCAG ATTGGGTGTG

4141 TTTGTAGTGG AGGACAGTGG TACGTTTTGG AAACAGTCTT CTTGAAAGTG TCTTGTCTAC

4201 AGTATATTCA CTCATAACCT CAATAGCCAA GGGTGTAGTC GGTTTATTAA AGATAACTTC

4261 GTATAATGTA TGCTATACGA AGTTATCTGA GGTGTCTCAC AAGTGCCGTG CAGTCCCGCC

4321 CCCACTTGCT TCTCTTTGTG TGTAGTGTAC GTACATTATC GAGACCGTTG TTCCCGCCCA

4381 CCTCGATCCG GCTGAGGTGT CTCACAAGTG CCGTGCAGTC CCGCCCCCAC TTGCTTCTCT

4441 TTGTGTGTAG TGTACGTACA TTATCGAGAC CGTTGTTCCC GCCCACCTCG ATCCGGCTGA

4501 GGTGTCTCAC AAGTGCCGTG CAGTCCCGCC CCCACTTGCT TCTCTTTGTG TGTAGTGTAC

4561 GTACATTATC GAGACCGTTG TTCCCGCCCA CCTCGATCCG GCTGAGGTGT CTCACAAGTG

4621 CCGTGCAGTC CCGCCCCCAC TTGCTTCTCT TTGTGTGTAG TGTACGTACA TTATCGAGAC

4681 CGTTGTTCCC GCCCACCTCG ATCCGGCACG GGCAAAAGTG CGTATATATA CAAGAGCGTT

4741 TGCCAGCCAC AGATTTTCAC TCCACACACC ACATCACACA TACAACCACA CACATCCACA

4801 ATGGAACCCG AAACTAAGAT GACTGACACT TCAAACATCA AGCCTGTCGC TGACATTGCC

4861 CTCATCGGTC TCGCCGTCAT GGGCCAGAAC CTGATCCTCA ACATGGCCGA CCACGGCTAC

4921 GAGGTTGTTG CCTACAACCG AACCACCTCC AAGGTGGACC ACTTCCTGGA GAACGAGGCC

4981 AAGGGAAAGT CCATTATTGG TGCTCACTCT ATCAAGGAGC TGTGTGCTCT GCTGAAGCGA

5041 CCCCGACGAA TCATTCTGCT CGTTAAGGCC GGTGCTGCTG TCGATTCTTT CATCGAACAG

5101 CTCCTGCCCT ATCTCGATAA GGGTGATATC ATCATTGACG GTGGTAACTC CCACTTCCCC

5161 GACTCCAACC GACGATACGA GGAGCTTAAC GAGAAGGGAA TCCTCTTTGT TGGTTCCGGT

5221 GTTTCCGGCG GTGAGGAGGG TGCCCGATAC GGTCCCTCCA TCATGCCCGG TGGAAACAAG

5281 GAGGCCTGGC CCCACATTAA GAAGATTTTC CAGGACATCT CTGCTAAGGC TGATGGTGAG

5341 CCCTGCTGTG ACTGGGTCGG TGACGCTGGT GCCGGCCACT TTGTCAAGAT GGTTCACAAC

5401 GGTATTGAGT ATGGTGACAT GCAGCTTATC TGCGAGGCTT ACGACCTCAT GAAGCGAGGT

5461 GCTGGTTTCA CCAATGAGGA GATTGGAGAC GTTTTCGCCA AGTGGAACAA CGGTATCCTC

5521 GACTCCTTCC TCATTGAGAT CACCCGAGAC ATCTTCAAGT ACGACGACGG CTCTGGAACT

5581 CCTCTCGTTG AGAAGATCTC CGACACTGCT GGCCAGAAGG GTACTGGAAA GTGGACCGCT

5641 ATCAACGCTC TTGACCTTGG TATGCCCGTC ACCCTGATCG GTGAGGCCGT CTTCGCTCGA

5701 TGCCTTTCTG CCCTCAAGCA GGAGCGTGTC CGAGCTTCCA AGGTTCTTGA TGGCCCCGAG

5761 CCCGTCAAGT TCACTGGTGA CAAGAAGGAG TTTGTGGACC AGCTGGAGCA GGCCCTTTAC

5821 GCCTCCAAGA TCATCTCTTA CGCCCAGGGT TTCATGCTTA TCCGAGAGGC CGCCAAGACC

5881 TACGGCTGGG AGCTGAACAA CGCCGGTATT GCCCTCATGT GGCGAGGTGG TTGCATCATC

5941 CGATCCGTCT TCCTTGCTGA CATCACCAAG GCTTACCGAC AGGACCCCAA CCTGGAGAAC

6001 CTGCTGTTCA ACGACTTCTT CAAGAACGCC ATCTCCAAGG CCAACCCCTC TTGGCGAGCT

6061 ACCGTGGCCA AGGCTGTCAC CTGGGGTGTT CCCACTCCCG CCTTTGCCTC GGCTCTGGCT

6121 TTCTACGACG GTTACCGATC TGCCAAGCTC CCCGCTAACC TGCTCCAGGC CCAGCGAGAC

6181 TACTTCGGAG CCCACACCTA CCAGCTCCTC GATGGTGATG GAAAGTGGAT TCACACCAAC

6241 TGGACCGGCC GAGGTGGTGA GGTTTCTTCT TCCACTTACG ATGCTTAAAG GTTAGACTAT

6301 GGATATGTCA TTTAACTGTG TATATAGAGA GCGTGCAAGT ATGGAGCGCT TGTTCAGCTT

6361 GTATGATGGT CAGACGACCT GTCTGATCGA GTATGTATGA TACTGCACAA CCTGTGTATC

6421 CGCATGATCT GTCCAATGGG GCATGTTGTT GTGTTTCTCG ATACGGAGAT GCTGGGTACA

6481 AGTAGCTAAT ACGATTGAAC TACTTATACT TATATGAGGC TTGAAGAAAG CTGACTTGTG

6541 TATGACTTAT TCTCAACTAC ATCCCCAGTC ACAATACCAC CACTGCACTA CCACTACACC

6601 AAAGGCAGAC ACTGCGTCGC TCCGTCCACA TCATCAACCG CCCCAGAACT GGTACGGACA

6661 AGGGGAATCT GACTGTCTAA TTAAAACATA GCTTTGCGAT GGTTGTAAAA CAATGTTGAC

6721 GCAAAGTGAT TTCTGCCCAG TGCTCTGAAT GTCAAAGTGA AGAAATTCAA CCAAGCGCGC

6781 GGGTAAACGG CGGGAGTAAC TATGCTCTCT TAAGGTAGCC AAATGCCTCG TCATCTAATT

6841 AGTGACGCGC ATGAATGGAT TAACGAGATT CCCACTGTCC CTATCTACTA TGTAGCGAAA

6901 CCACAGCCAA GGGAACGGGC TTGGCAGAAT CAGCGGGGAA AGAAGACCCT GTTGAGCTTG

6961 ACTCTAGTTT GACATTGTGA AGAGACATAG GGGGTGTAGA ATAAGTGGGA GCTTCGGCGC

7021 CGGTGAAATA CCACTACCCT TATCGTTTCT TTACTTATTT AGTAAGTGGA AGTGGTTTAA

7081 CAACCATTTT CTAGCATTCC TTTCCAGGCT GAAGACATTG TCAGGTGGGG AGTTTGGCTG

7141 GGGCGGCACA TCTGTTAAAA GATAACGCAG ATGTCCTAAG GGGGACTCAA TGAGAACAGA

7201 AAATGCGGCC GC


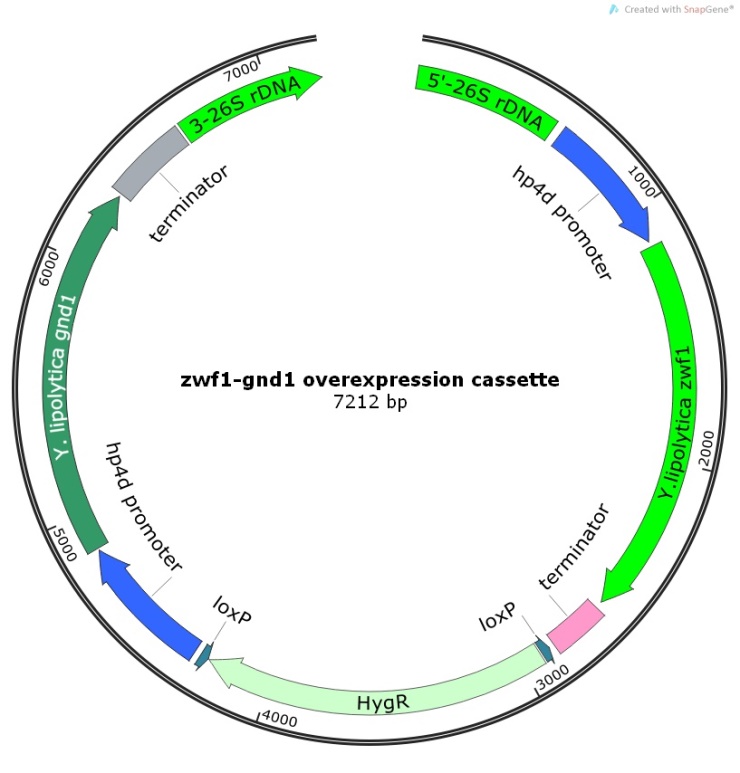

Supplement: Supplementary file 1 — Additional file 1. [file 13068_2020_1815_MOESM1_ESM.docx]
